# Maternal Aldehyde Elimination during Pregnancy Preserves the Fetal Genome

**DOI:** 10.1016/j.molcel.2014.07.010

**Published:** 2014-09-18

**Authors:** Nina Oberbeck, Frédéric Langevin, Gareth King, Niels de Wind, Gerry P. Crossan, Ketan J. Patel

**Affiliations:** 1 MRC Laboratory of Molecular Biology, Francis Crick Avenue, Cambridge CB2 0QH, UK; 2 Department of Toxicogenetics, Leiden University Medical Center, P.O. Box 9600, 2300 RC Leiden, the Netherlands; 3 Department of Medicine, Level 5, Addenbrooke’s Hospital, University of Cambridge, Cambridge CB2 0QQ, UK

## Abstract

Maternal metabolism provides essential nutrients to enable embryonic development. However, both mother and embryo produce reactive metabolites that can damage DNA. Here we discover how the embryo is protected from these genotoxins. Pregnant mice lacking Aldh2, a key enzyme that detoxifies reactive aldehydes, cannot support the development of embryos lacking the Fanconi anemia DNA repair pathway gene *Fanca*. Remarkably, transferring *Aldh2*^−/−^*Fanca*^−/−^ embryos into wild-type mothers suppresses developmental defects and rescues embryonic lethality. These rescued neonates have severely depleted hematopoietic stem and progenitor cells, indicating that despite intact maternal aldehyde catabolism, fetal Aldh2 is essential for hematopoiesis. Hence, maternal and fetal aldehyde detoxification protects the developing embryo from DNA damage. Failure of this genome preservation mechanism might explain why birth defects and bone marrow failure occur in Fanconi anemia, and may have implications for fetal well-being in the many women in Southeast Asia that are genetically deficient in *ALDH2*.

## Highlights

• Maternal aldehydes must be catabolized to prevent fetal DNA damage
• Fetal DNA repair counteracts DNA damage caused by maternal aldehydes
• *Aldh2*^−/−^*Rev1*^−/−^ mice do not phenocopy *Aldh2*^−/−^*Fanca*^−/−^ mice
• Maternal and fetal endogenous aldehydes corrupt development and hematopoiesis

## Graphical Abstract

**Figure:**
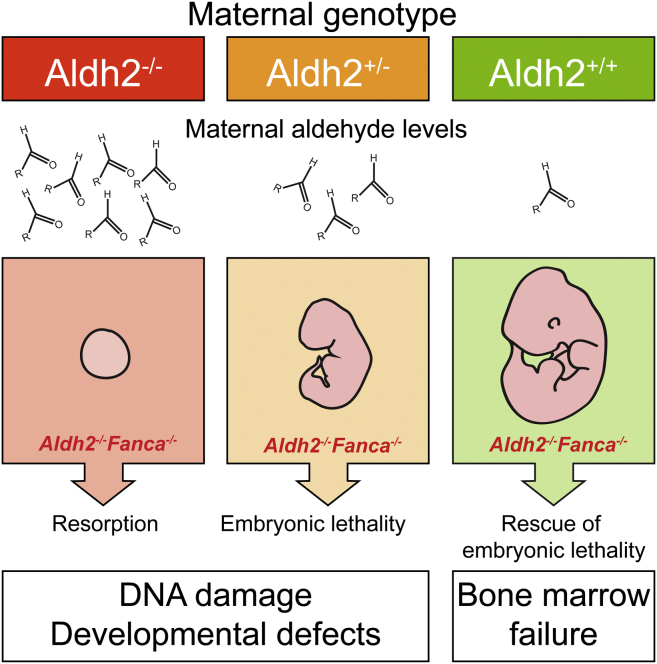

## Teaser

During pregnancy, both mother and embryo produce reactive metabolites that can damage DNA. Oberbeck et al. find that there are distinct spatial and temporal roles for maternal and fetal aldehyde detoxification in protecting the developing embryo from DNA damage, and thus from accumulating developmental defects.

## Introduction

Birth defects are common and a substantial burden to human health, but their etiology is complex and often due to many factors. Maternal exposure to X-rays and chemotherapeutic agents give rise to birth defects, mainly because these agents cause direct damage to the fetal genome (Arnon et al., 2001, Hall, 1991, Streffer et al., 2003, Toledo et al., 1971). The developing embryo limits this damage through DNA repair, thus attenuating the potential of these mutagens to corrupt development. This is further underscored by striking developmental phenotypes associated with humans that are genetically defective in certain DNA repair pathways.

Children afflicted with Fanconi anemia (FA) have a genetic deficiency in DNA repair and are often born with a multitude of birth defects affecting many organs, combined with intrauterine growth retardation (Alter and Rosenberg, 2013). Provided these defects are not so severe as to cause early death, most FA patients develop bone marrow failure and are cancer prone (Alter, 2003, Kutler et al., 2003). FA results from an inability to repair DNA crosslinks, a specific form of DNA damage where the two complementary strands of DNA are covalently linked. This disease is genetically heterogeneous with germline mutations in any one of sixteen genes (*FANCA-Q*) resulting in the disease (Garaycoechea and Patel, 2014). Despite the significant progress made in identifying the genes responsible for FA and defining how their gene products cooperate to repair DNA crosslinked by chemotherapeutic agents (such as cisplatin), our understanding of how this DNA repair defect leads to congenital abnormalities and bone marrow failure is limited (Hodskinson et al., 2014, Klein Douwel et al., 2014, Knipscheer et al., 2009, Räschle et al., 2008). In addition, whilst the developmental defects associated with FA clearly relate to a fundamental role for DNA crosslink repair during embryogenesis, it is unclear how the intrauterine environment contributes to the eventual loss of bone marrow function.

We recently showed that a major physiological function of the FA DNA repair pathway is to protect the genome from damage caused by endogenous aldehydes (Garaycoechea et al., 2012, Langevin et al., 2011, Rosado et al., 2011). These highly reactive molecules are byproducts of many metabolic pathways, such as lipid peroxidation and the breakdown of alcohols. Mice that lack both the aldehyde-detoxifying enzyme Aldh2 and the key FA protein Fancd2 are cancer prone and develop bone marrow failure (Garaycoechea et al., 2012, Langevin et al., 2011). Here we address whether metabolically derived aldehydes cause DNA damage to the developing embryo. Our results reveal how maternal and fetal aldehyde catabolism cooperate with fetal FA DNA repair to preserve development.

## Results

### A Role for *Aldh2* and Only Certain DNA Crosslink Repair Genes in Development

*Aldh2*^−/−^*Fancd2*^−/−^ mice generated in a hybrid genetic background (C57BL6/Jo1a × 129S6/SvTac) succumb to leukemia and possess very few hematopoietic stem cells (HSCs). These double-deficient mice cannot be born to *Aldh2*^−/−^ mothers but can be born to mothers that are heterozygous for *Aldh2* (*Aldh2*^+/−^), intimating a crucial developmental role for maternal aldehyde catabolism (Langevin et al., 2011). In order to understand this essential maternal requirement for *Aldh2* in the development of DNA repair-deficient embryos, we initially set out to establish three key points: (1) what contribution the mouse genetic background might have on this phenomenon, (2) whether the genetic requirement for DNA repair was generalizable to a FA repair gene upstream of *Fancd2*, and (3) if this interaction was a feature of DNA crosslink repair generally or restricted only to the FA repair genes.

**Figure 1:**
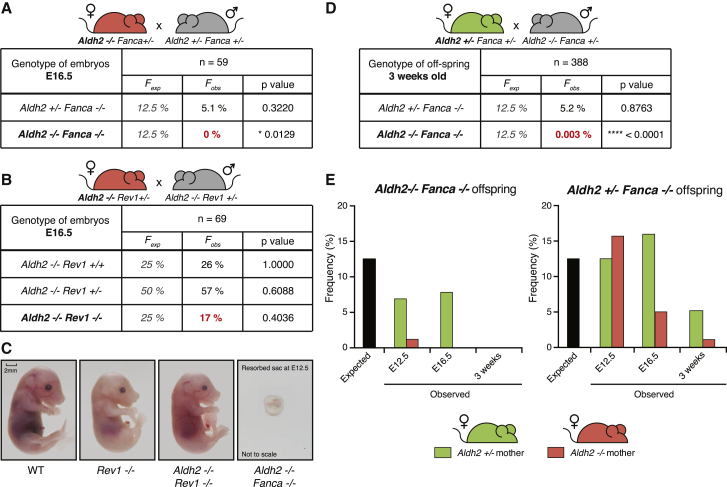
*Aldh2* Is Essential for the Development of *Fanca*^−/−^, but Not *Rev1*^−/−^, Embryos (A) Observed and expected frequencies of *Aldh2*^+/−^*Fanca*^−/−^ and *Aldh2*^−/−^*Fanca*^−/−^ fetuses at E16.5, obtained from *Aldh2*^−/−^*Fanca*^+/−^ females (red) intercrossed with *Aldh2*^+/−^*Fanca*^+/−^ males (gray). Genotyped resorptions were not included in the data. Fisher’s exact test, 5% confidence interval. (B) Observed and expected frequencies of *Aldh2*^−/−^*Rev1*^+/+^, *Aldh2*^−/−^*Rev1*^+/−^, and *Aldh2*^−/−^*Rev1*^−/−^ fetuses at E16.5, obtained from intercrossing *Aldh2*^−/−^*Rev1*^+/−^ females (red) and males (gray). Genotyped resorptions were not included in the data. Fisher’s exact test, 5% confidence interval. (C) Representative images of E16.5 fetuses of various genotypes referred to in the table in (B). The *Aldh2*^−/−^*Rev1*^−/−^ and *Aldh2*^−/−^*Fanca*^−/−^ embryos were both generated from *Aldh2*^−/−^ females. The *Aldh2*^−/−^*Fanca*^−/−^ resorbed sac was at E12.5. (D) Observed and expected frequencies of *Aldh2*^+/−^*Fanca*^−/−^ and *Aldh2*^−/−^*Fanca*^−/−^ mice at 3 weeks of age, obtained from *Aldh2*^+/−^*Fanca*^+/−^ females (green) intercrossed with *Aldh2*^−/−^*Fanca*^+/−^ males (gray). Fisher’s exact test, 5% confidence interval. (E) Bar graphs show frequencies of *Aldh2*^−/−^*Fanca*^−/−^ and *Aldh2*^+/−^*Fanca*^−/−^ offspring at E10.5–E12.5, E16.5, and 3 weeks of age, generated from either *Aldh2*^+/−^*Fanca*^+/−^ females (green bars) or *Aldh2*^−/−^*Fanca*^+/−^ females (red bars). These females were intercrossed with *Aldh2*^−/−^*Fanca*^+/−^ and *Aldh2*^+/−^*Fanca*^+/−^ males, respectively. The expected frequency is represented by the black bar (12.5%). Genotyped resorptions are not included in the data. Refer to Figure S1 for complete data and statistics.

Autosomal recessive mutations in any one of sixteen genes (*FANCA-Q*) can be mutated in FA, but almost 60% of the cases are due to mutations in the *FANCA* gene (Neveling et al., 2009). We therefore set out to generate mice that lack *Aldh2* and *Fanca* (*Aldh2*^−/−^*Fanca*^−/−^) in a pure C57BL6/Jo1a background. In the first instance, we mated *Aldh2*^−/−^*Fanca*^+/−^ females with *Aldh2*^+/−^*Fanca*^+/−^ males and found that no *Aldh2*^−/−^*Fanca*^−/−^ mice were weaned (Figure S1A, left table, available online). This result indicates that *Aldh2* deficiency is synthetically lethal with *Fanca* deficiency, similar to what has already been reported for *Aldh2* and *Fancd2* in the hybrid background. To learn more about the timing of this synthetic lethal interaction, we looked earlier in development, at embryonic day 16.5 (E16.5), and found that *Aldh2*^−/−^*Fanca*^−/−^ fetuses were already absent in late gestation (Figure 1A).

Genetic and biochemical evidence has shown that DNA translesion synthesis (TLS) mediated by Rev1 is essential for DNA crosslink repair. Moreover, Rev1 and the FA proteins function in a common pathway to maintain resistance to DNA interstrand crosslinking agents (Niedzwiedz et al., 2004, Ross et al., 2005). Mice lacking *Rev1* are born at lower than expected Mendelian frequency, and their postnatal life is very similar to, or even slightly more severe than that of FA knockout mice (Jansen et al., 2006). We therefore asked if *Aldh2*^*−/−*^*Rev1*^*−/−*^ embryos were viable in late gestation when conceived by *Aldh2*^−/−^ mothers. Surprisingly, *Aldh2*^−/−^*Rev1*^−/−^ E16.5 fetuses are readily detected and appear developmentally intact and similar to *Rev1*^−/−^ or wild-type controls (Figures 1B and 1C). This is in stark contrast to *Aldh2*^−/−^*Fanca*^−/−^ embryos, which are completely resorbed by E12.5. We finally asked if a single maternal allele of *Aldh2* would enable *Aldh2*^−/−^*Fanca*^−/−^ mice to be born. This was particularly important since *Aldh2*^−/−^*Fancd2*^−/−^ mice bred in the C57BL6/Jo1a × 129S6/SvTac background are viable only when conceived by *Aldh2*^+/−^ mothers. Surprisingly, *Aldh2*^−/−^*Fanca*^−/−^ mice in the pure C57BL6/Jo1a background are not viable, even when generated from the same cross that allowed the birth of *Aldh2*^−/−^*Fancd2*^−/−^ mice (Figure 1D). We also noted that *Aldh2*^+/−^*Fanca*^−/−^ mice are not born at the expected ratio. When *Aldh2*^+/−^*Fanca*^−/−^ pups are conceived by *Aldh2*^−/−^*Fanca*^+/−^ mothers, they are observed at a reduced frequency of 1.1% by 3 weeks (expected 12.5%, ^∗^p = 0.0114). This is mirrored in *Aldh2*^+/−^*Fanca*^−/−^ mothers, where there is a tendency toward reduced numbers of *Aldh2*^+/−^*Fanca*^−/−^ pups (ns, p = 0.8763). These data demonstrate a fetal Aldh2 haploinsufficient effect by which one fetal allele of *Aldh2* is insufficient to fully rescue the embryonic lethality of *Fanca*^−/−^ embryos (Figures S1A and 1D).

We then determined the point at which *Aldh2*^−/−^*Fanca*^−/−^ and *Aldh2*^+/−^*Fanca*^−/−^ embryos died during gestation, from either *Aldh2*^−/−^ or *Aldh2*^+/−^ mothers (Figures 1E, S1A, S1B, and S1C). This revealed that *Aldh2*^−/−^ mothers resorb *Aldh2*^−/−^*Fanca*^−/−^ embryos early in gestation, prior to E12.5, but these embryos survive longer in *Aldh2*^+/−^ mothers, with fetuses being evident at E16.5. We also noted that a single allele of fetal *Aldh2* enables *Fanca*^−/−^ embryos to proceed further in gestation, but their viability is also impacted by maternal aldehyde catabolism. This indicates that although the maternal *Aldh2* status has a profound influence on development, fetal *Aldh2* also provides protection. In summary, maternal and fetal Aldh2 is essential for the viability of *Fanca*^−/−^ embryos, but surprisingly this is not the case for *Rev1*^−/−^ embryos, in a directly comparable pure C57BL6/Jo1a genetic background.

### Maternal *Aldh2* Is Critical for the Development of *Fanca*-Deficient Embryos and in Their Protection against Exogenous Ethanol

**Figure 2:**
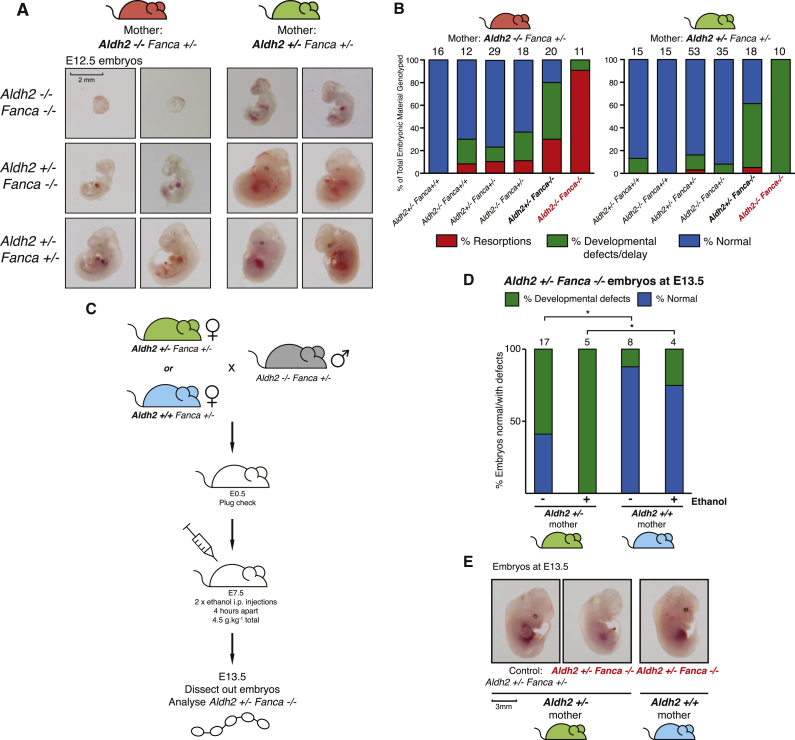
Nature of Spontaneous and Ethanol-Induced Developmental Defects in *Fanca*^−/−^ Embryos in Relation to Maternal *Aldh2* (A) Representative images of *Aldh2*^−/−^*Fanca*^−/−^ and *Aldh2*^+/−^*Fanca*^−/−^ embryos, in comparison to *Aldh2*^+/−^*Fanca*^+/−^ controls, at E12.5, generated from either *Aldh2*^−/−^*Fanca*^+/−^ (red mouse, left) or *Aldh2*^+/−^*Fanca*^+/−^ (green mouse, right) females. (B) Bar graph displaying the proportion of E10.5–E12.5 embryos that are normal (blue), have developmental defects/delay (green), and are resorptions (red) out of the total embryonic material genotyped. Embryos were generated from either *Aldh2*^−/−^*Fanca*^+/−^ (red mouse, left graph) or *Aldh2*^+/−^*Fanca*^+/−^ (green mouse, right graph) females. The total number of embryos of each genotype is shown above each column. (C) Schematic representation of the experiment to expose pregnant *Aldh2*^+/+^*Fanca*^+/−^ or *Aldh2*^+/−^*Fanca*^+/−^ females to ethanol. (D) Bar graph displaying the proportion of *Aldh2*^+/−^*Fanca*^−/−^ E13.5 embryos that are normal (blue) and have developmental defects (green). These embryos were generated from either *Aldh2*^+/−^*Fanca*^+/−^ females (green mouse) or *Aldh2*^+/+^*Fanca*^+/−^ females (blue mouse) who had either been exposed to ethanol at E7.5 of pregnancy or not. Genotyped resorptions were not included in the data. Fisher’s exact test was used to compare the proportion of embryos with developmental defects between *Aldh2*^+/−^ and *Aldh2*^+/+^ mothers; ^∗^p < 0.05, 5% confidence interval. The total number of embryos of each genotype is shown above each column. (E) Representative images of control and *Aldh2*^+/−^*Fanca*^−/−^ E13.5 embryos, generated from either an *Aldh2*^+/−^*Fanca*^+/−^ female (green mouse) or an *Aldh2*^+/+^*Fanca*^+/−^ female (blue mouse), following exposure to ethanol during pregnancy.

We next sought to comprehensively analyze the manner in which the development of *Aldh2*^−/−^*Fanca*^−/−^ and *Aldh2*^+/−^*Fanca*^−/−^ embryos is impacted when they are conceived by either *Aldh2*^−/−^ (red) or *Aldh2*^+/−^ (green) mothers (Figure 2A). We quantified the data in Figure 2A by genotyping embryos conceived by the two maternal genotypes and scoring them into three groups (normal, blue; embryos that were delayed in development or carried anatomical defects, green; embryos that were resorbed, red; Figure 2B). Representative images of the embryos of the three main genotypes in Figure 2A show first that *Aldh2*^−/−^*Fanca*^−/−^ embryos are dying from a widespread failure in development. When conceived by *Aldh2*^−/−^ mothers, double mutants are mostly resorbed by E12.5. However, when conceived by *Aldh2*^+/−^ mothers, double mutants are still intact at E12.5, but all have developmental defects including eye, limb, and craniofacial abnormalities (Figures 2B, S2A, and S2B). Similarly, *Aldh2*^+/−^*Fanca*^−/−^ embryos, when conceived by *Aldh2*^−/−^ mothers, are resorbed and show developmental defects, and a single maternal allele of *Aldh2* suppresses these resorptions (Figures 2B, S2A, and S2B). There is a wide spectrum of developmental abnormalities affecting multiple organ systems, which may reflect the stochastic nature of DNA damage (Figures S2C and S2D). *Aldh2*^+/−^*Fanca*^−/−^ mice born to *Aldh2*^−/−^ mothers are rare; however, they can be born to *Aldh2*^+/−^ mothers (albeit at a lower Mendelian frequency than expected), and they have a similar survival to *Fanca*^−/−^ mice. The Kaplan-Meier curve demonstrates that *Aldh2*^+/−^*Fanca*^−/−^ mice born to *Aldh2*^−/−^ mothers have a severely reduced survival compared to the same mice born to Aldh2-proficient mothers (Figure S3). In summary, these data highlight that maternal Aldh2 deficiency has a profound impact on FA-deficient embryos. This is best illustrated by the fact that the presence of just one maternal allele of *Aldh2* almost completely suppresses resorption of *Aldh2*^−/−^*Fanca*^−/−^ embryos at E12.5 (green mouse symbol, Figure 2B).

The origin, identity, and sites of clearance of the physiologically relevant toxic aldehyde(s) within the mother are unclear. Ethanol is an important source of acetaldehyde, which is the key substrate for Aldh2 and causes developmental failure in fetal alcohol syndrome (O’Shea and Kaufman, 1979, Webster et al., 1983). We therefore reasoned that challenging mothers carrying *Fanca*^−/−^ embryos with ethanol should provide direct evidence for an aldehyde causing damage to the embryos. When *Aldh2*^+/−^ mothers are exposed to an acute dose of ethanol (4.5 g/kg) during early pregnancy (E7.5), all *Aldh2*^+/−^*Fanca*^−/−^ embryos are developmentally compromised (this is not the case in the absence of ethanol; Figures 2C and 2D). We next asked whether an extra maternal allele of *Aldh2* could protect these embryos from ethanol-derived aldehyde-mediated damage. When *Aldh2*^+/+^ mothers were given exogenous ethanol during gestation, *Aldh2*^+/−^*Fanca*^−/−^ embryos remained developmentally intact and were significantly protected, in strong contrast to the same embryos from an *Aldh2*^+/−^ mother (Figures 2D and 2E). The striking effect of maternal ethanol challenge clearly demonstrates that maternal haploinsufficiency leads to reduced fetal protection to aldehydes. Cumulatively, these results show the extent to which maternal aldehyde catabolism deficiency impacts the development of *Aldh2*^−/−^*Fanca*^−/−^ and *Aldh2*^+/−^*Fanca*^−/−^ embryos.

### Expression of Acetaldehyde-Catabolizing Enzymes during Pregnancy

**Figure 3:**
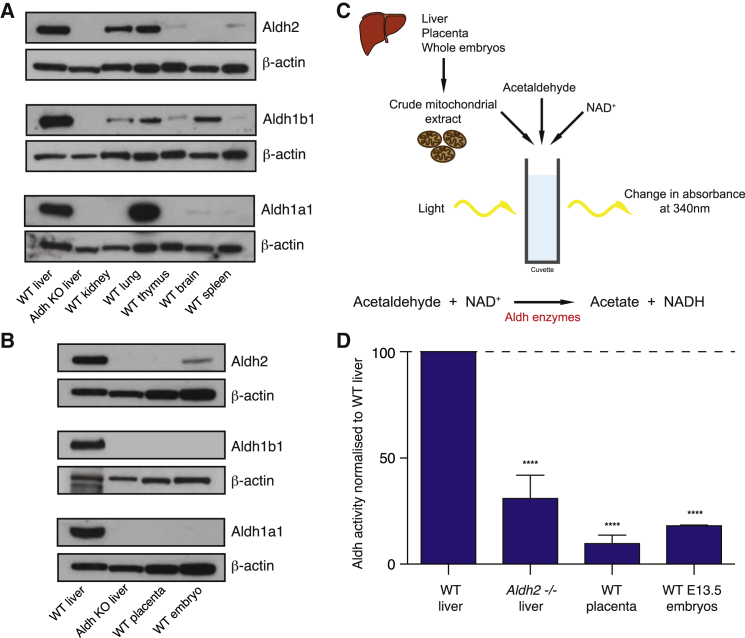
Expression and Activity of Acetaldehyde-Catabolizing Enzymes in the Fetal-Maternal Unit (A) Western blot showing the expression of Aldh2, Aldh1b1, and Aldh1a1 in whole-cell extracts of a panel of tissues taken from a wild-type mouse. Expression of beta-actin is shown as a loading control. Whole-cell extracts from the corresponding livers obtained from *Aldh* knockout females is shown as a control for the specificity of each antibody. (B) Western blot showing the expression of Aldh2, Aldh1b1, and Aldh1a1 in whole-cell extracts of wild-type (WT) liver, placenta, and E12.5 embryo. Expression of beta-actin is shown as a loading control. The corresponding *Aldh* knockout in the liver is again run alongside tissue samples to control for the specificity of each antibody. (C) Schematic of the Aldh activity assay. Acetaldehyde is converted to acetate by Aldh2 (and other Aldh enzymes), which produces NADH. The production of NADH, and thus the Aldh activity, can be measured in a crude mitochondrial extract by the addition of acetaldehyde and assessment of the rate of change of absorbance at 340 nm. (D) Aldh2 activity in the mouse wild-type (WT) liver, *Aldh2*^−/−^ liver, WT placenta, and WT E13.5 embryo. Aldh activities were normalized to WT liver. Unpaired t test, ^∗∗∗∗^p < 0.0001, 5% confidence interval. Error bars represent SD. n = 7 for WT and *Aldh2*^−/−^ liver, n = 3 for placenta, n = 2 for embryos.

Given the importance of maternal aldehyde catabolism for the development of *Fanca*^−/−^ embryos, we next wanted to determine where aldehyde catabolism occurs within the fetal-maternal unit. To address this, we looked at the expression of Aldh2 and two closely related aldehyde dehydrogenases, Aldh1a1 and Aldh1b1, in the mother and embryo (Figure S4). These two enzymes are known to also catabolize acetaldehyde (Stagos et al., 2010, Yoshida et al., 1992). The maternal liver expresses all three Aldh enzymes at high levels, with expression also present in most maternal tissues (Figure 3A). However, it came as a particular surprise that the placenta shows no detectable Aldh2, Aldh1a1, and Aldh1b1 expression (Figure 3B); this organ is the interface between the maternal and fetal circulation. In addition, whole E12.5 embryos show very low levels of Aldh2 protein, a striking difference to that observed for the maternal liver. It is possible that another Aldh-class enzyme might be expressed in the placenta and the embryo. To address this, we carried out an enzymatic assay for the catabolism of acetaldehyde (Figure 3C). We used acetaldehyde, as it is a known substrate of Aldh2, Aldh1b1, and Aldh1a1. In addition, the ethanol challenge experiment described above shows that acetaldehyde can damage *Aldh2*^+/−^*Fanca*^−/−^ embryos conceived by *Aldh2*^+/−^ mothers. This assay measures the turnover of NAD^+^ (an essential cofactor for aldehyde dehydrogenases) in crude mitochondrial extracts incubated with acetaldehyde. Maternal liver extract shows very robust acetaldehyde catabolism that is greatly reduced in an *Aldh2*^−/−^ liver extract (Figure 3D). Activity in the *Aldh2*^−/−^ liver is not completely absent due to redundant mitochondrial and contaminating cytosolic enzymes. The aldehyde catabolism activity in the placenta and E13.5 embryo extract is low; in fact, it is similar to that of an *Aldh2*^−/−^ liver. This indicates that the fetus and placenta may not be able to contribute to aldehyde detoxification to the same extent as that of the mother in early development.

### *Aldh2* Deficiency Leads to the Accumulation of DNA Damage in *Fanca*^−/−^ Embryos

**Figure 4:**
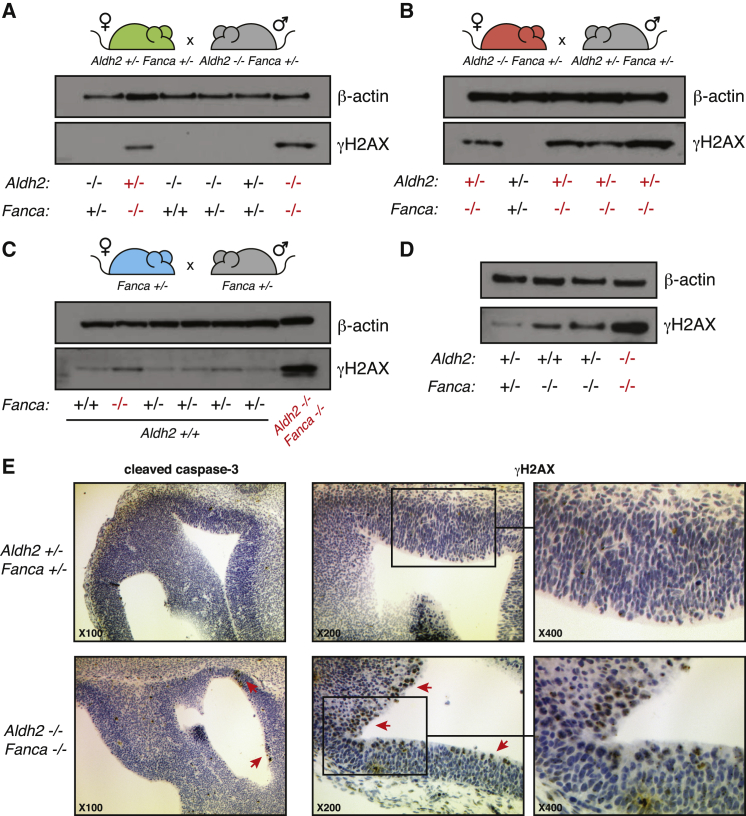
Aldehyde Catabolism Prevents DNA Damage in *Fanca*-Deficient Embryos (A) Western blot showing the detection of γH2AX in whole-cell extracts made from individual whole E12.5 embryos (where the fetal livers had been removed). Individual embryos from a single litter, generated from the mating displayed, are shown. *Aldh2*^−/−^*Fanca*^−/−^ and *Aldh2*^+/−^*Fanca*^−/−^ embryos can be clearly distinguished from littermates through the presence of γH2AX. Expression of beta-actin is shown as a loading control. (B) Western blot showing the detection of γH2AX in whole-cell extracts made from individual whole E12.5 embryos (where the fetal livers had been removed). Individual embryos from a single litter, generated from the mating displayed, are shown. *Aldh2*^+/−^*Fanca*^−/−^ embryos can be clearly distinguished from littermates through the presence of γH2AX. Expression of beta-actin is shown as a loading control. (C) Western blot showing the detection of γH2AX in whole-cell extracts made from individual whole E12.5 embryos (where the fetal livers had been removed). Individual embryos from a single litter, generated from the mating displayed, are shown. Expression of beta-actin is shown as a loading control. (D) Western blot comparing the relative detection of γH2AX in whole-cell extracts made from individual whole E12.5 embryos of the relevant genotypes (not all from the same litter). Expression of beta-actin is shown as a loading control. (E) Immunohistochemistry for cleaved caspase-3 (×100) or γH2AX (×200, inset ×400) of sections of the periventricular region of the developing brain, from an *Aldh2*^+/−^*Fanca*^+/−^ or an *Aldh2*^−/−^*Fanca*^−/−^ embryo at E12.5. Red arrows denote cells staining positive for cleaved caspase-3 or γH2AX.

The striking developmental phenotype of *Aldh2*^−/−^*Fanca*^−/−^ and *Aldh2*^+/−^*Fanca*^−/−^ embryos when conceived by either *Aldh2*^−/−^ or *Aldh2*^+/−^ mothers led us to ask whether this correlated with an accumulation of DNA damage. DNA double-strand breaks (DSBs) in the nucleus stimulate the phosphorylation of histone H2AX (γH2AX), which is readily detected by western blot analysis (Rogakou et al., 1998). We therefore made whole-cell extracts from E12.5 embryos generated from both *Aldh2*^+/−^ and *Aldh2*^−/−^ mothers. Strikingly, *Aldh2*^−/−^*Fanca*^−/−^ and *Aldh2*^+/−^*Fanca*^−/−^ embryos can be clearly distinguished from their littermates through the strong presence of γH2AX, a marker of DNA damage (Figures 4A and 4B). While *Aldh2*^+/+^*Fanca*^−/−^ control embryos do show a γH2AX signal, this is far less striking than the induction observed for *Aldh2*^−/−^*Fanca*^−/−^ embryos (Figure 4C). In addition, *Aldh2*^−/−^*Fanca*^−/−^ embryos have the most γH2AX compared to *Aldh2*^+/−^*Fanca*^−/−^ and *Fanca*^−/−^ embryos (Figure 4D).

We also looked at the induction of γH2AX and cleaved caspase-3 (a marker of apoptosis) by immunohistochemistry. We stained sections from the periventricular region of developing brain of E12.5 embryos (conceived by an *Aldh2*^+/−^ mother). This is an easily identifiable region of the brain of the embryo, with a defined anatomy, which allows direct comparison between genotypes. It is immediately apparent that many more cells in this region of *Aldh2*^−/−^*Fanca*^−/−^ embryos stain strongly for nuclear γH2AX and cleaved capase-3 versus control (Figure 4E). These results indicate that deficiency of maternal and fetal aldehyde catabolism results in the accumulation of DNA damage and increased apoptosis in *Fanca*-deficient embryos.

### Rescue of Embryonic Lethality, but Not Hematopoietic Stem Cells, when *Aldh2*^−/−^*Fanca*^−/−^ Mice Are Carried by *Aldh2*^+/+^ Mothers

**Figure 5:**
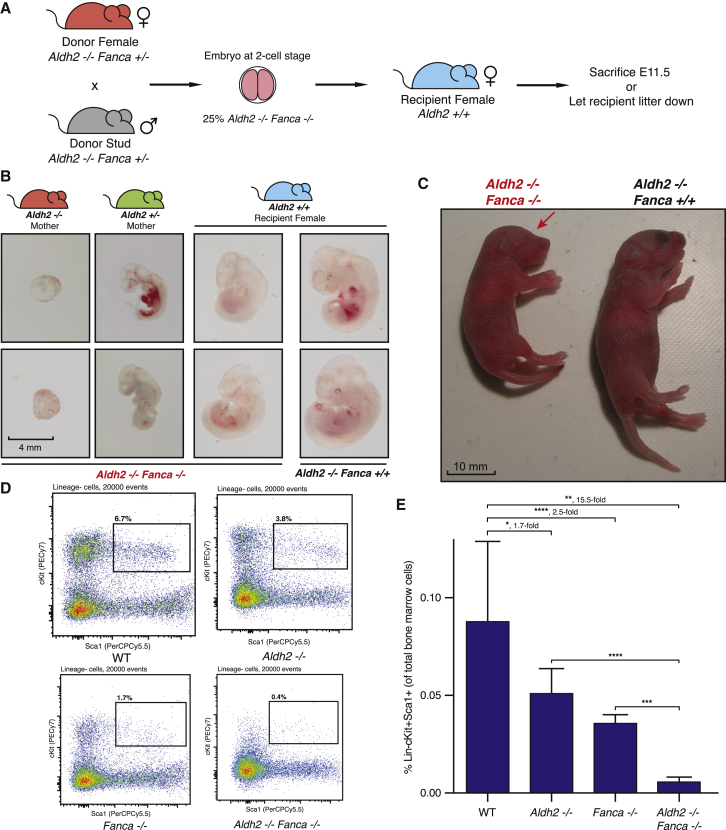
Embryo Transfer of *Aldh2*^−/−^*Fanca*^−/−^ Embryos into *Aldh2*^+/+^ Mothers Rescues Their Development but Not Blood Stem Cells (A) Schematic overview of the embryo transfer experiment. Embryos at the two-cell stage obtained from intercrossing *Aldh2*^−/−^*Fanca*^+/−^ males (gray) and females (red) were harvested and transferred into the uteri of mothers completely competent at aldehyde catabolism (*Aldh2*^+/+^; blue mouse). (B) Representative images of *Aldh2*^−/−^*Fanca*^−/−^ embryos at E11.5, generated from either *Aldh2*^−/−^*Fanca*^+/−^ females (left, red mouse), *Aldh2*^+/−^*Fanca*^+/−^ females (middle, green mouse), or *Aldh2*^+/+^ recipient females by embryo transfer (right, blue mouse). *Aldh2*^−/−^*Fanca*^+/+^ embryos at E11.5, generated by embryo transfer into *Aldh2*^+/+^ females, are shown as controls. *Aldh2*^+/+^ mothers suppress the developmental defects of *Aldh2*^−/−^*Fanca*^−/−^ embryos at E11.5. (C) Photograph of an *Aldh2*^−/−^*Fanca*^−/−^ pup at P1 (1 day postpartum) next to an *Aldh2*^−/−^*Fanca*^+/+^ littermate control. Both pups were born to an *Aldh2*^+/+^ mother, using embryo transfer. Red arrow shows the lack of an eye in the *Aldh2*^−/−^*Fanca*^−/−^ pup. (D) Representative flow cytometry profiles of bone marrow cells obtained from P1 pups of various genotypes born from embryo transfer (for *Aldh2*^−/−^*Fanca*^−/−^ pups and *Aldh2*^−/−^ control) or through natural crosses (for WT and *Fanca*^−/−^ controls). The cells were stained for lineage markers, c-Kit and Sca-1, and the profiles show 20,000 lineage-negative events. The box denotes the Lin^−^cKit^+^Sca1^+^ (LKS) population, which is enriched for hematopoietic stem cells. (E) Bar chart showing the quantification of hematopoietic stem and progenitor cell (HSPC) populations assessed by flow cytometry in (D). Unpaired t test; ^∗^p < 0.05, ^∗∗^p < 0.005, ^∗∗∗^p < 0.0005, ^∗∗∗^p < 0.0001, 5% confidence interval. Error bars represent SD. n = 12 for WT, n = 24 for Aldh2^−/−^, n = 3 for Fanca^−/−^, n = 3 for *Aldh2*^−/−^*Fanca*^−/−^.

So far we have shown that the maternal capacity to remove aldehydes protects the embryo from accumulating endogenous DNA damage and developmental defects. Although this maternal detoxification is important, we also know that fetal Aldh2 and DNA repair provide protection. In order to disassociate how much maternal and fetal aldehyde catabolism contributes to ensure development, we sought to determine if complete aldehyde detoxification by the mother could rescue developmental attrition of *Aldh2*^−/−^*Fanca*^−/−^ embryos, using embryo transfer experiments (as natural genetic crosses do not allow us to address this question). Embryos at the two-cell stage obtained from crossing *Aldh2*^−/−^*Fanca*^+/−^ males and females were harvested and transferred into the uteri of mothers completely competent at aldehyde catabolism (*Aldh2*^+/+^; Figure 5A). This embryo transfer experiment allows us to interrogate the developmental fate of *Aldh2*^−/−^*Fanca*^−/−^ embryos, which would otherwise never survive after E12.5 in an *Aldh2*^−/−^ mother or E16.5 in an *Aldh2*^+/−^ mother. Remarkably, we noted that *Aldh2*^+/+^ mothers suppress developmental defects of *Aldh2*^−/−^*Fanca*^−/−^ embryos at E11.5. A total of 25% of *Aldh2*^−/−^*Fanca*^−/−^ embryos from embryo transfer show developmental defects (n = 8), compared to 100% of *Aldh2*^−/−^*Fanca*^−/−^ embryos conceived by *Aldh2*^+/−^ mothers (n = 10) at E11.5 (^∗∗^p = 0.0015; Figure 5B). Furthermore, when such surrogate pregnancies are allowed to come to term, *Aldh2*^−/−^*Fanca*^−/−^ mice are born for the first time (Figures 5C and S5A). However, these mice are smaller than their littermates and have a high prevalence of developmental defects, including craniofacial abnormalities (Figure 5C, data not shown).

The hallmark of FA is the development of bone marrow failure due to loss of the HSC pool (Ceccaldi et al., 2012). Recent evidence in humans and mice suggests that stem cell defects can be detected in utero (Ceccaldi et al., 2012, Kamimae-Lanning et al., 2013). Our previous work has shown that aldehyde-mediated DNA damage might explain HSC attrition in adult mice, and in this study we show that aldehydes have a profound impact on the development of FA-deficient embryos (Garaycoechea et al., 2012). Now that *Aldh2*^−/−^*Fanca*^−/−^ mice can be rescued through gestation into neonatal life, we wanted to see if these mice had intact hematopoietic stem and progenitor cells (HSPCs) in their bone marrow. The data in Figures 5D and 5E show clearly that there is a significant 15.5-fold depletion of the Lin^−^cKit^+^Sca1^+^ (LKS) population (representing the HSPC pool) of these mice, compared to a 2.5-fold depletion in the *Fanca*^−/−^ control.

Furthermore, one single *Aldh2*^*−/−*^*Fanca*^*−/−*^ mouse born naturally to an *Aldh2*^*+/−*^ mother survived the embryonic window and was weaned (a rare occurrence of 1 out of 388 mice). In agreement with the fate of *Aldh2*^*−/−*^*Fanca*^*−/−*^ mice born from embryo transfer, this mouse developed bone marrow failure at 7 weeks of age (Figure S5B). Analysis of the bone marrow revealed a severe loss of LKS cells, showing that bone marrow failure was due to depletion of HSPCs. Cumulatively, these data allow us to disassociate the role of maternal and fetal aldehyde catabolism during development. The embryo transfer experiment conclusively demonstrates that wild-type maternal *Aldh2* is necessary and sufficient for the birth of *Aldh2*^*−/−*^*Fanca*^*−/−*^ mice. In other words, maternal catabolism is crucial for general development to proceed, but embryonic Aldh2 is essential to prevent development defects and for the overall preservation of blood stem cells.

## Discussion

**Figure 6:**
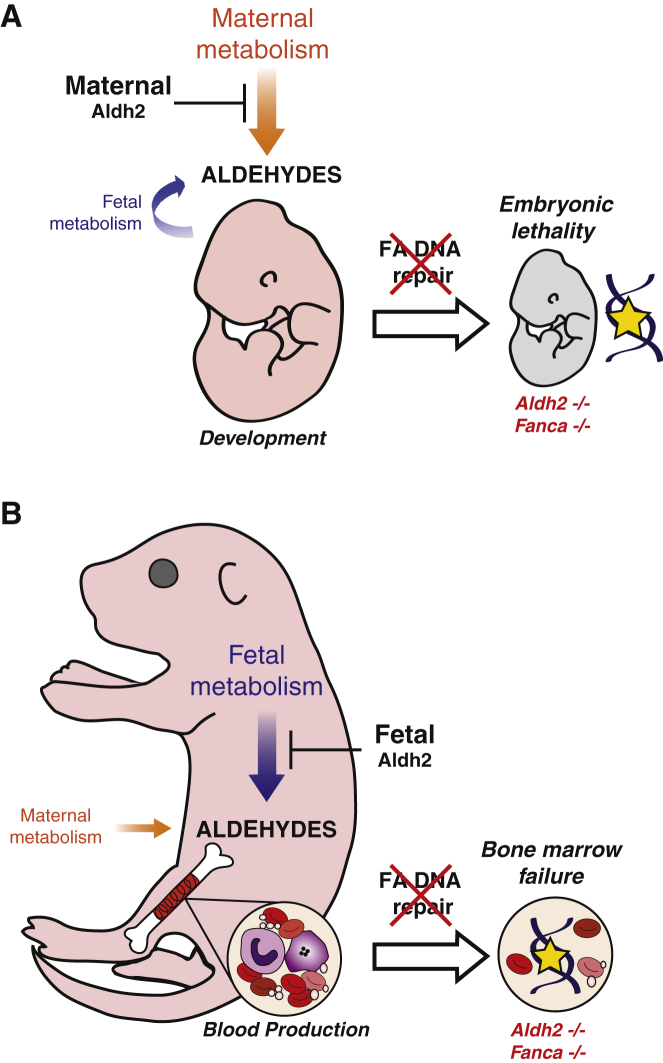
Model for the Role of Aldehyde Catabolism and FA DNA Repair during Development (A) In early development, maternal metabolism produces aldehydes, which diffuse across the placenta and damage the DNA of the developing embryo. Fetal metabolism may also produce aldehydes, which disrupt development. Maternal and fetal Aldh2 play a key role in detoxifying these aldehydes and protecting the early embryo from developmental defects. (B) In later development, the embryo is larger and produces a greater aldehyde burden from fetal metabolism; fetal Aldh2 counteracts this in order to prevent developmental defects. In addition, there is an organism intrinsic requirement for Aldh2 and FA DNA repair in the fetus, which together are required to protect bone marrow stem cells from accumulating DNA damage and undergoing attrition.

The work presented in this paper defines a basic mechanism for the protection of the growing embryo from genotoxic aldehydes. This mechanism operates at three levels. First, the mother detoxifies aldehydes that she produces from her circulation. Second, the embryo is reliant on the FA DNA repair pathway to respond to and repair DNA damage caused by these aldehydes that have evaded both maternal and fetal removal. Third, embryonic aldehyde catabolism is essential to preserve bone marrow HSPCs in utero (Figure 6).

Aldehyde removal through maternal Aldh2 is the primary protective mechanism to shield the early embryo from these genotoxins. There is increasing evidence to show that early insults to the fetus, including DNA damage, can lead to disease in adult life, a process known as intrauterine programming (Fowden et al., 2005). Human epidemiological studies have revealed that impaired intrauterine growth, due to lack of availability of nutrients, oxygen, or hormones during pregnancy, is linked to the development of diabetes and other metabolic disorders (Fowden et al., 2006). In addition, mice with a hypomorphic mutation in the DNA damage response protein ATR develop a progeroid syndrome, and this phenotype is due to high levels of replicative stress and DNA damage during embryogenesis, but not in adult tissues (Murga et al., 2009). In agreement with previous work on intrauterine programming, our study suggests that in utero exposure to aldehydes, which in certain circumstances can lead to the accumulation of DNA damage in the embryo, may be a mechanism that explains the origin of congenital abnormalities in humans. Our data also point to acetaldehyde-mediated DNA damage in utero being responsible for the developmental defects seen in the fetal alcohol syndrome, which is caused by excessive maternal ethanol consumption during pregnancy. Lastly, given the evidence that childhood leukemias are often initiated in utero, this raises an intriguing possibility that aldehyde-induced DNA damage during gestation contributes to early genetic changes that cause these neoplasmas (Clarkson and Boyse, 1971, Greaves and Wiemels, 2003, van Dijk et al., 1996).

The genetic interaction between the mother and the embryo presented in this paper is highly unusual in the mammal. Genetic screens in flies and zebrafish have uncovered many instances of this class of interaction; however, this is mainly due to a requirement for maternal RNA to express essential proteins very early in development (Anderson and Nüsslein-Volhard, 1984, Nüsslein-Volhard et al., 1987, Pelegri et al., 1999). In mammals, there are just two other clear examples of maternal lethal zygotic effects that affect later stages of embryonic development (Li et al., 2008, Rutschmann et al., 2012). Our work further underscores the importance of considering the maternal genotype (in addition to allele segregation) when assessing embryonic phenotypes. In this case, it seems most likely that the maternal biomass, which in early gestation is considerably larger than the embryo, produces acetaldehyde or another substrate of Aldh2. The widespread expression of Aldh2 would suggest that these aldehydes are probably broken down before they access the embryo. Two other enzymes (Aldh1a1 and Aldh1b1) are also capable of removing the same aldehydes; however, our results indicate that Aldh2 deficiency cannot be fully compensated by them. This may be due to the lower enzymatic efficiency or subcellular localization of these enzymes (Klyosov et al., 1996, Peng and Yin, 2009, Stagos et al., 2010). We were surprised to note that the placenta seems completely devoid of expression of any of the three Aldhs, and our enzymatic assay confirms negligible activity in placenta from wild-type mice. It is possible that an endogenous aldehyde may be required for the normal physiology of this tissue, which would explain the absence of aldehyde dehydrogenase activity.

Maternal embryo transfer allowed us to further confirm the relevance of maternal versus fetal aldehyde catabolism. Our results show that *Aldh2*^+/+^ mothers suppress the embryonic lethality of *Aldh2*^−/−^*Fanca*^−/−^ mice. However, these embryos are developmentally compromised, indicating that fetal Aldh2 is required to prevent developmental abnormalities. This is further emphasized by the severe lack of HSPCs in the bone marrow of these neonates. It would seem that as the embryo grows, its own metabolism generates aldehydes, which, if not cleared by the fetus, damage HSPCs and lead to their attrition. Alternatively, there may be a cell-intrinsic requirement for Aldh2 and FA DNA repair in the HSPCs themselves in order to protect them against damage and attrition. Nevertheless, these results are in agreement with recent studies that indicate that bone marrow dysfunction in FA begins in utero (Ceccaldi et al., 2012, Kamimae-Lanning et al., 2013).

So far, we have focused on how the DNA of the embryo is shielded from aldehydes; the other key protection mechanism is fetal DNA repair. The FA pathway acts in a DNA crosslink repair process that requires the Rev1 protein (Niedzwiedz et al., 2004, Ross et al., 2005). However, neither maternal nor fetal Aldh2 appear essential for the development of *Aldh2*^−/−^*Rev1*^−/−^ embryos. In fact, these embryos can be conceived by *Aldh2*^−/−^ mothers and appear indistinguishable from *Rev1*^−/−^ embryos. Furthermore, *Aldh2*^−/−^*Rev1*^−/−^ neonates do not have a reduced HSPC pool compared to *Rev1*^−/−^ pups (Figure S6B). Consistent with these findings, *Rev1*^−/−^ hematopoietic cells are no more sensitive to acetaldehyde than wild-type cells (Figure S6A).

The phenotype of *Aldh2*^−/−^*Rev1*^−/−^ diverges significantly from the phenotype of *Aldh2*^−/−^*Fanca*^−/−^ mice. This is a surprising result, as Rev1 and the FA proteins act in a common pathway to maintain resistance to chemotherapy-induced crosslinking agents. These data suggest that the genetic requirements to repair DNA damage caused by an Aldh2 substrate differ significantly from the requirements to repair canonical chemotherapy-induced crosslinks. Future work will be needed to define precisely the chemical nature of DNA damage created by aldehydes, as this may not be a simple DNA interstrand crosslink.

As already mentioned, FA is characterized by developmental defects and bone marrow failure (Alter, 2003, Kutler et al., 2003). In most instances, the mother and embryo are likely to be aldehyde catabolism proficient. However, it is possible that in certain instances aldehyde levels in the embryo might spike—most obviously when a mother consumes alcohol in the first trimester of pregnancy. Such situations would require intact FA repair in order to reverse aldehyde-induced DNA damage. Approximately 540 million individuals worldwide (mainly across Southeast Asia and Japan) are genetically deficient in *ALDH2* (*ALDH2^∗^2* allele) (Brooks et al., 2009). The molecular defect is due to single amino acid substitution (E487K) that causes a dominant-negative mutant form of ALDH2, resulting in heterozygotes having only 5%–15% of wild-type ALDH2 activity (Brooks et al., 2009, Harada et al., 1981, Yoshida et al., 1981, Yoshida et al., 1984). This provides a unique opportunity to study the impact of ALDH2 deficiency and FA in humans. A recent study looked at the clinical progression of FA in children of Japanese origin that also segregate the *ALDH2* mutant allele, *ALDH2^∗^2* (Hira et al., 2013). This study clearly shows that ALDH2 deficiency leads to a much more rapid progression of bone marrow failure. However, this study also shows that ALDH2 deficiency does not lead to more developmental defects. This is a surprise when put into the context of our work in mice, but it is worth noting two factors: first, the genotype of the mothers is not known, and second, two of the three patients that were homozygous for *ALDH2^∗^2* carried extensive developmental malformations. These children could only be born to *ALDH2^∗^2* heterozygous or homozygous mothers. Nevertheless, it will be crucial to combine the phenotypic analysis of Southeast Asian FA patients with their maternal *ALDH2* status.

Finally, the many humans carrying the *ALDH2^∗^2* allele are known to be sensitive to alcohol, and alcohol consumption in this group enhances their risk of developing head and neck cancer (Brooks et al., 2009). However, it is not known if this common allele also impacts on fetal wellbeing and the prevalence of birth defects, particularly with alcohol consumption during pregnancy. Future studies should address this question, which is of clear importance for public health and preventative medicine in this populous part of the world.

## Experimental Procedures

Please refer to the Supplemental Experimental Procedures for detailed methodology on flow cytometry, survival assays, and comprehensive aldehyde dehydrogenase activity assay conditions.

### Mice

*Aldh2*^−/−^ and *Fanca*^−/−^ mice on a C57BL6/Jo1a background were described previously (Garaycoechea et al., 2012, Langevin et al., 2011). *Aldh2*-deficient mice were generated from embryonic stem cells (ESCs) obtained from EUCOMM (*Aldh2*^*tm1a(EUCOMM)Wtsi*^; Mouse Genome Informatics [MGI] code: 4431566). *Fanca*-deficient mice were generated from ESCs, obtained from EUCOMM (*Fanca*^*tm1a(EUCOMM)Wtsi*^; MGI code: 4434431). *Rev1*-deficient mice were a gift from N. de Wind (*Rev1*^*tm1Ndew*^; MGI code: 3701945) and described previously (Jansen et al., 2006). Once imported, these mice were maintained in the C57BL6/Jo1a background. All animals were maintained in specific pathogen-free conditions. All animal experiments undertaken in this study were done so with the approval of the UK Home Office.

### Histology

Histological analysis was performed on whole embryos fixed in neutral buffered formalin for 24 hr. The samples were then paraffin embedded, and 4 μm sections were cut before being stained with hematoxylin and eosin. For immunohistochemistry, samples were cut and stained as described previously (Langevin et al., 2011), using rabbit anti-phospho-H2AX (Cell Signaling, 2577, 1:50) and rabbit anti-cleaved caspase-3 (Cell Signaling, Asp175 9661L, 1:100).

### Flow Cytometry

Flow cytometry was performed on bone marrow, spleen, and thymic cells that were isolated from mutant mice and appropriate controls. Bone marrow cells were obtained either by flushing from the femora and tibiae (adult bones) or by crushing the femora, tibiae, and humeri (P1 pups) and passing through a 40 μm filter. The cells were stained as described previously (Garaycoechea et al., 2012), and samples were run on a LSRII flow cytometer (BD Pharmingen), and the data were analyzed with FlowJo 10.0.7 (Tree Star).

### Timed Matings

Timed matings of *Aldh2*^−/−^*Fanca*^+/−^, *Aldh2*^+/−^*Fanca*^+/−^, and *Fanca*^+/−^ females were set up with corresponding males. Females were checked for the presence of a vaginal plug the following morning, which was considered day E0.5. Females were then killed at E10.5–E12.5 or E16.5, and embryos or resorbed sacs were removed for genotyping and analysis. For the acute ethanol during pregnancy experiment, plugged females were injected with 4.5 g/kg of a 28% ethanol solution at E7.5 in two separate intraperitoneal injections of 2.25 g/kg, 4 hr apart. At E13.5, pregnant females were killed and uteri taken for dissection of embryos.

### Western Blotting

Western blot extracts were prepared from whole individual embryos (with the fetal livers removed) or various tissues by disruption in RIPA buffer (0.1% SDS, 50 mM Tris-HCl [pH 7.4], 150 mM NaCl, 0.5% Na-deoxycholate, 1% NP-40, 1 mM EDTA, serine/threonine phosphatase inhibitor cocktail (PhosphoStop, Sigma-Aldrich), and protease inhibitor cocktail (Roche) using a QIAGEN TissueLyser II. γH2AX monoclonal antibody (Millipore, JBW301) was used at 1:1,000. Beta-actin polyclonal antibody (Abcam, ab8227) was used at 1:2,000. Aldh2 (Proteintech, 15310-1-AP), Aldh1b1 (Proteintech, 15560-1-AP), and Aldh1a1 (Proteintech, 15910-1-AP) polyclonal antibodies were used at 1:2,000.

### Embryo Transfer

*Aldh2*^−/−^*Fanca*^+/−^ female donor mice (4 weeks old) were injected with five international units of pregnant mare’s serum (PMS) and human chorionic gonadotrophin (hCG) intraperitoneally on day 1. On day 3, they were placed overnight with a proven *Aldh2*^−/−^*Fanca*^+/−^ stud, and on day 4 potentially pregnant donors were identified by the presence of a vaginal plug. These females were sacrificed, and two-cell-stage embryos were flushed from their uteri. These were washed in M2 media and maintained in M16 media under mineral oil in a CO_2_ incubator until being transferred into recipients. On day 3, recipient wild-type C57BL6/Jo1a females, weighing 20–30 g, were mated overnight with vasectomized (CD1) males to produce pseudopregnant recipients. These were identified on day 4 as being positive for a vaginal plug, and up to 16 two-cell embryos were then implanted into their uteri unilaterally in a surgical manner. The recipient females were then sacrificed 11 days after embryo transfer for dissection of embryos or allowed to litter down for analysis of P1 pups.

### Aldehyde Dehydrogenase Activity Assay

Mouse livers, whole E13.5 embryos, or placentas were homogenized in order to prepare a crude mitochondrial extract. This extract was subsequently used to perform Aldh enzymatic activity assays. Reactions were set up in a cuvette containing the crude mitochondrial extract, NaPPi buffer (pH 9.0), NAD^+^, and the substrate acetaldehyde. After substrate addition, the absorbance at 340 nm was recorded using a Cary 5000 UV-Vis-NIR spectrophotometer at room temperature (RT) for 350 s. To calculate the [NADH] production in mol/min/mg total protein, we used the equation absorbance = ε × c × L, where ε = 6220 M^−1^, L = path length (1 cm), and c = [NADH] in mol. The assay was adapted from a protocol by D. Mochly-Rosen, Stanford University.

## Author Contributions

N.O., F.L., G.P.C., and K.J.P. designed the study and wrote the paper. N.O. performed the majority of experiments presented. F.L. helped characterize developmental failure throughout the study and analyzed Rev1 mice. N.d.W. provided *Rev1*-deficient mice. G.K. performed embryo transfer experiments.

## Supplemental Information

Document S1. Supplemental Experimental Procedures and Figures S1–S6

Document S2. Article plus Supplemental Information
