# Supplementary material for: Maternal Aldehyde Elimination during Pregnancy Preserves the Fetal Genome
Source: Mol Cell. 2014 Sep 18;55(6):807–17. doi: 10.1016/j.molcel.2014.07.010 (PMC4175174; doi:10.1016/j.molcel.2014.07.010)
Supplement: Document S1. Supplemental Experimental Procedures and Figures S1–S6 [file mmc1.pdf]

Molecular Cell, Volume *55*

Supplemental Information

Maternal Aldehyde Elimination during Pregnancy Preserves the Fetal Genome

Nina Oberbeck, Frédéric Langevin, Gareth King, Niels de Wind, Gerry P. Crossan, and Ketan J. Patel

Figure S1

A

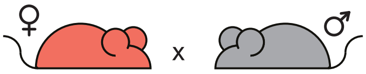

Aldh2 -/- *Fanca+/-* x *Aldh2 +/- Fanca +/-*

Genotype of off-spring **3 weeks old**	n = 186		
	F_{exp}	F_{obs}	p value
Aldh2 +/- Fanca +/+	*12.5 %*	15.1% n = 28	0.8865
Aldh2 -/- Fanca +/+	*12.5 %*	18.8% n = 35	0.5852
Aldh2 +/- Fanca +/-	*25 %*	25.8% n = 48	0.8146
Aldh2 -/- Fanca +/-	*25 %*	39.2% n = 73	* 0.0217
Aldh2 +/- Fanca -/-	***12.5 %***	**1.1% n = 2**	*** 0.0114**
Aldh2 -/- Fanca -/-	***12.5 %***	**0% n = 0**	***** 0.0004**

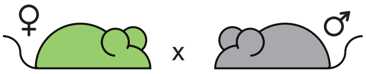

Aldh2 +/- *Fanca +/-* x *Aldh2 -/- Fanca +/-*

Genotype of off-spring **3 weeks old**	n = 388		
	F_{exp}	F_{obs}	p value
Aldh2 +/- Fanca +/+	*12.5 %*	15.5% n = 60	0.9215
Aldh2 -/- Fanca +/+	*12.5 %*	18.6% n = 72	0.3927
Aldh2 +/- Fanca +/-	*25 %*	31.2% n = 121	0.3056
Aldh2 -/- Fanca +/-	*25 %*	29.4% n = 114	0.6332
Aldh2 +/- Fanca -/-	***12.5 %***	**5.2% n = 20**	**0.8763**
Aldh2 -/- Fanca -/-	***12.5 %***	**0.003% n = 1**	****** < 0.0001**

B

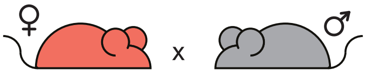

Aldh2 -/- *Fanca+/-* x *Aldh2 +/- Fanca +/-*

Genotype of embryos **E16.5**	n = 59		
	F_{exp}	F_{obs}	p value
Aldh2 +/- Fanca +/+	*12.5 %*	10.2% n = 6	1.0000
Aldh2 -/- Fanca +/+	*12.5 %*	22% n = 13	0.2193
Aldh2 +/- Fanca +/-	*25 %*	25.4% n = 15	1.0000
Aldh2 -/- Fanca +/-	*25 %*	37.3% n = 22	0.2336
Aldh2 +/- Fanca -/-	***12.5 %***	**5.1% n = 3**	**0.3220**
Aldh2 -/- Fanca -/-	***12.5 %***	**0% n = 0**	*** 0.0129**

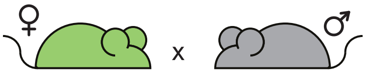

Aldh2 +/- *Fanca +/-* x *Aldh2 -/- Fanca +/-*

Genotype of embryos **E16.5**	n = 64		
	F_{exp}	F_{obs}	p value
Aldh2 +/- Fanca +/+	*12.5 %*	25% n = 16	0.1117
Aldh2 -/- Fanca +/+	*12.5 %*	7.8% n = 5	0.5600
Aldh2 +/- Fanca +/-	*25 %*	20.3% n = 13	0.6733
Aldh2 -/- Fanca +/-	*25 %*	23.4% n = 15	1.0000
Aldh2 +/- Fanca -/-	***12.5 %***	**15.6% n = 10**	**0.8000**
Aldh2 -/- Fanca -/-	***12.5 %***	**7.8% n = 5**	**0.5600**

C

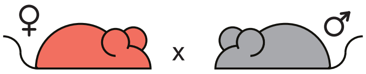

Aldh2 -/- *Fanca+/-* x *Aldh2 +/- Fanca +/-*

Genotype of embryos **E10.5-E12.5**	n = 83		
	F_{exp}	F_{obs}	p value
Aldh2 +/- Fanca +/+	*12.5 %*	19.3% n = 16	0.2855
Aldh2 -/- Fanca +/+	*12.5 %*	13.3% n = 11	1.0000
Aldh2 +/- Fanca +/-	*25 %*	31.3% n = 26	0.4910
Aldh2 -/- Fanca +/-	*25 %*	19.3% n = 16	0.4560
Aldh2 +/- Fanca -/-	***12.5 %***	**15.7% n = 13**	**0.6540**
Aldh2 -/- Fanca -/-	***12.5 %***	**1.2% n = 1**	**** 0.0093**

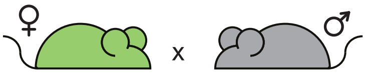

Aldh2 +/- *Fanca +/-* x *Aldh2 -/- Fanca +/-*

Genotype of embryos **E10.5-E12.5**	n = 144		
	F_{exp}	F_{obs}	p value
Aldh2 +/- Fanca +/+	*12.5 %*	10.4% n = 15	0.7119
Aldh2 -/- Fanca +/+	*12.5 %*	10.4% n = 15	0.7119
Aldh2 +/- Fanca +/-	*25 %*	35.4% n = 51	0.0721
Aldh2 -/- Fanca +/-	*25 %*	24.3% n = 35	1.000
Aldh2 +/- Fanca -/-	***12.5 %***	**12.5% n = 18**	**1.000**
Aldh2 -/- Fanca -/-	***12.5 %***	**6.9% n = 10**	**0.1629**

Figure S2

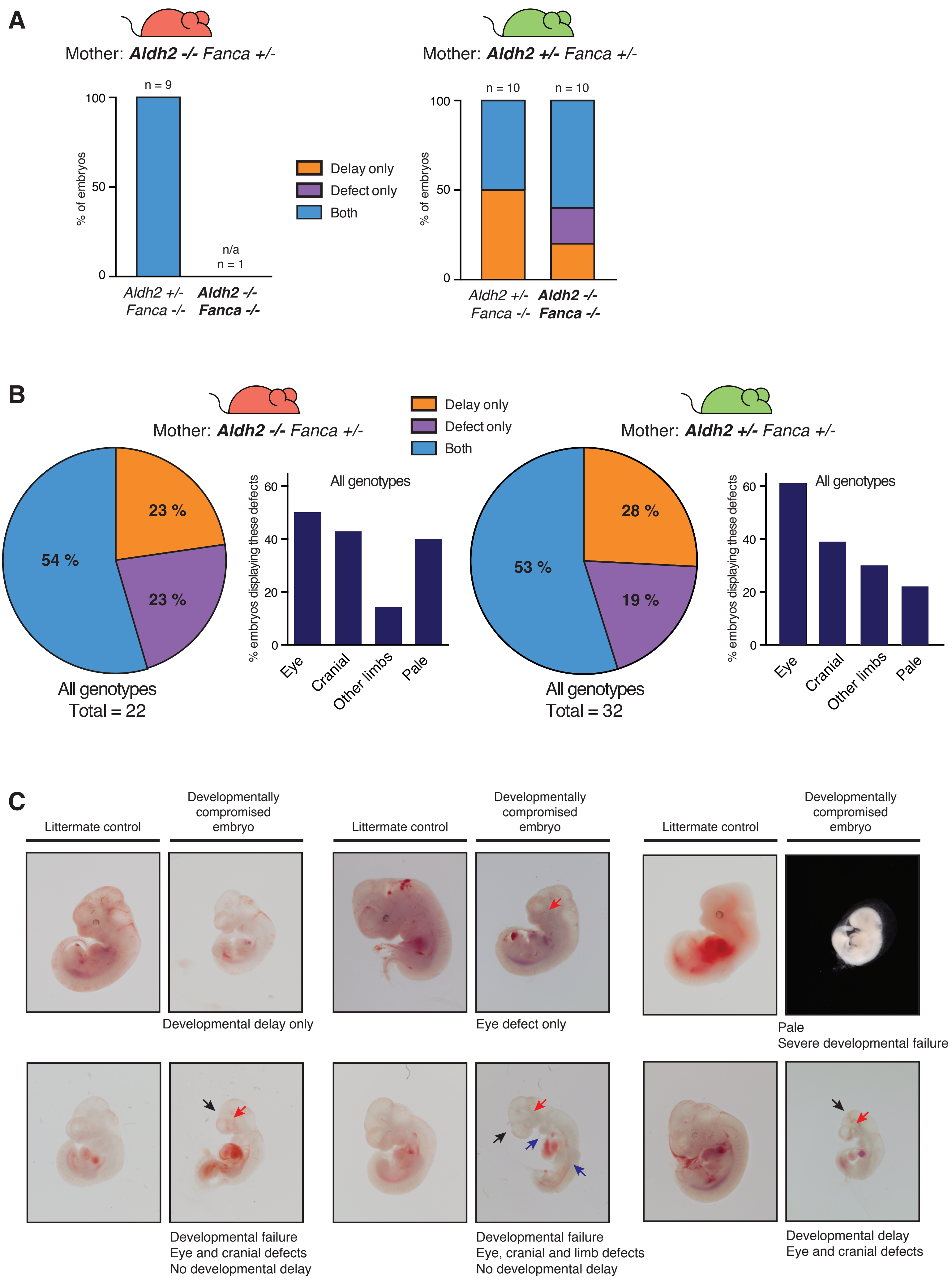

A
Mother: Aldh2 -/- Fanca +/-
n = 9
100
50
0
% of embryos
n/a
n = 1
Aldh2 +/- Fanca -/-
Aldh2 -/- Fanca -/-
Delay only
Defect only
Both
Mother: Aldh2 +/- Fanca +/-
n = 10
n = 10
Aldh2 +/- Fanca -/-
Aldh2 -/- Fanca -/-
B
Mother: Aldh2 -/- Fanca +/-
23 %
54 %
23 %
All genotypes
Total = 22
% embryos displaying these defects
All genotypes
60
40
20
0
Eye
Cranial
Other limbs
Pale
Mother: Aldh2 +/- Fanca +/-
28 %
53 %
19 %
All genotypes
Total = 32
C
Littermate control
Developmentally compromised embryo
Developmental delay only
Eye defect only
Pale
Severe developmental failure
Developmental failure
Eye and cranial defects
No developmental delay
Developmental failure
Eye, cranial and limb defects
No developmental delay
Developmental delay
Eye and cranial defects

Figure S3

A

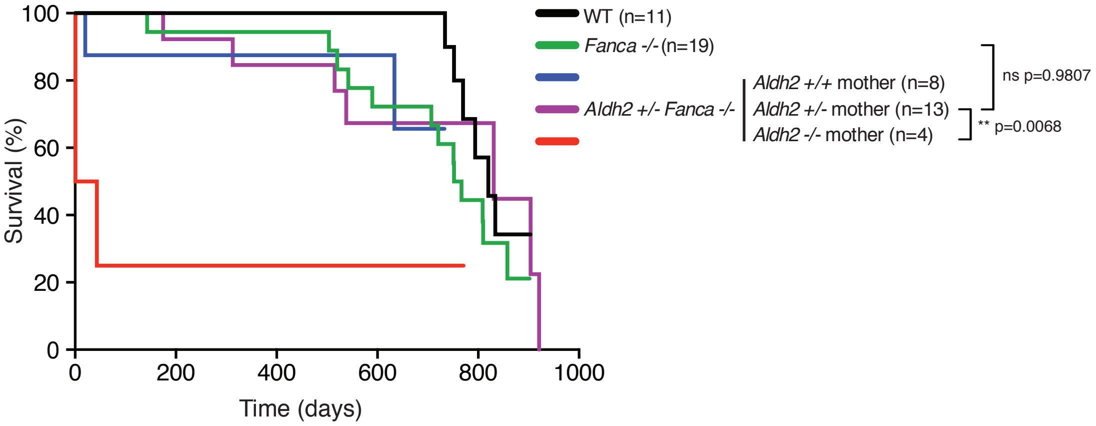

Survival (%)
100
80
60
40
20
0
0
200
400
600
800
1000
Time (days)
WT (n=11)
Fanca -/- (n=19)
Aldh2 +/- Fanca -/-
Aldh2 +/+ mother (n=8)
Aldh2 +/- mother (n=13)
Aldh2 -/- mother (n=4)
ns p=0.9807
** p=0.0068

B

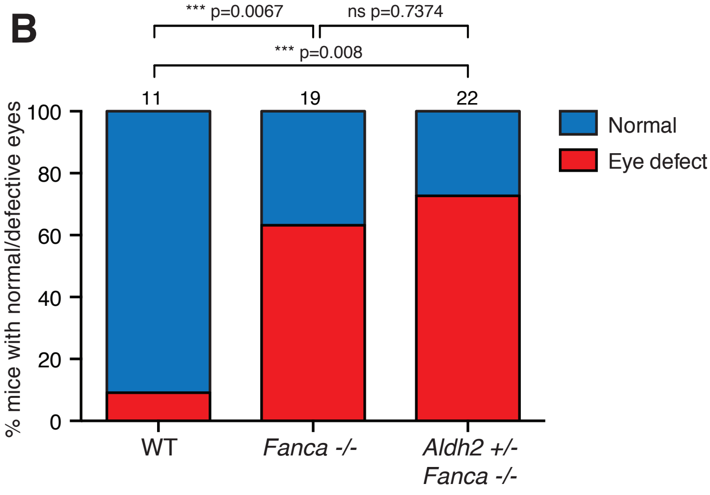

*** p=0.0067
ns p=0.7374
*** p=0.008
11
19
22
% mice with normal/defective eyes
100
80
60
40
20
0
WT
Fanca -/-
Aldh2 +/-
Fanca -/-
Normal
Eye defect

Figure S4

```
ALDH2_MOUSE      1  MLRAALTTVRRGPRLSRLLSAAATSAVPAPNHQPEVFCNQIFINNEWHDAVSRK   54
ALDH1B1_MOUSE    1  MLTARLLLPRLLCLQGRTTSYSTAAALPNPIPNPEICYNKLFINNEWHDAVSKK   54
ALDH1A1_MOUSE    1  ------------------MSSPAQPAVPAPLADLKIQHTKIFINNEWHNSVSGK   36

ALDH2_MOUSE     55  TFPTVNPSTGEVICQVAEGNKEDVDKAVKAARAAFQLGSPWRRMDASDRGRLLY  108
ALDH1B1_MOUSE   55  TFPTVNPTTGEVIGHVAEGDRADVDLAVKAAREAFRLGSPWRRMDASERGRLLN  108
ALDH1A1_MOUSE   37  KFPVLNPATEEVICHVEEGDKADVDKAVKAARQAFQIGSPWRTMDASERGRLLN   90

ALDH2_MOUSE    109  RLADLIERDRTYLAALETLDNGKPYVISYLVDLDMVLKCLRYYAGWADKYHGKT  162
ALDH1B1_MOUSE  109  RLADLVERDRVYLASLETLDNGKPFQESYVLDLDEVIKVYRYFAGWADKWHGKT  162
ALDH1A1_MOUSE   91  KLADLMERDRLLLATMEALNGGKVFANAYLSDLGGCIKALKYCAGWADKIHGQT  144

ALDH2_MOUSE    163  IPIDGDFFSYTRHEPVGVCGQIIPWNFPLLMQAWKLGPALATGNVVVMKVAEQT  216
ALDH1B1_MOUSE  163  IPMDGEHFCFTRHEPVGVCGQIIPWNFPLVMQGWKLAPALATGNTVVMKVAEQT  216
ALDH1A1_MOUSE  145  IPSDGDIFTYTRREPIGVCGQIIPWNFPMLMFIWKIGPALSCGNTVVVKPAEQT  198

ALDH2_MOUSE    217  PLTALYVANLIKEAGFPPGVVNIVPGFGPTAGAAIASHEGVDKVAFTGSTEVGH  270
ALDH1B1_MOUSE  217  PLSALYLASLIKEAGFPPGVVNIITGYGPTAGAAIAQHMDVDKVAFTGSTEVGH  270
ALDH1A1_MOUSE  199  PLTALHLASLIKEAGFPPGVVNIVPGYGPTAGAAISSHMDVDKVAFTGSTQVGK  252

ALDH2_MOUSE    271  LIQVAAGSSNLKRVTLELGGKSPNIIMSDADMDWAVEQAHFALFFNQGQCCCAG  324
ALDH1B1_MOUSE  271  LIQKAAGESNLKRVTLELGGKSPSIVLADADMEHAVDQCHEALFFNMGQCCCAG  324
ALDH1A1_MOUSE  253  LIKEAAGKSNLKRVTLELGGKSPCIVFADADLDIAVEFAHHGVFYHQGQCCVAA  306

ALDH2_MOUSE    325  SRTFVQENVYDEFVERSVARAKSRVVGNPFDSRTEQGPQVDETQFKKILGYIKS  378
ALDH1B1_MOUSE  325  SRTFVEESIYREFLERTVEKAKQRKVGNPFELDTQQGPQVDKEQFERILGYIRL  378
ALDH1A1_MOUSE  307  SRIFVEESVYDEFVKRSVERAKKYVLGNPLTPGINQGPQIDKEQHDKILDLIES  360

ALDH2_MOUSE    379  GQQEGAKLLCGGGAAADRGYFIQPTVFGDVKDGMTIAKEEIFGPVMQILKFKTI  432
ALDH1B1_MOUSE  379  GQKEGAKLLCGGERLGERGFFIKPTVFGDVQDGMRIAKEEIFGPVQPLFKFKKI  432
ALDH1A1_MOUSE  361  GKKEGAKLECGGGRWGNKGFFVQPTVFSNVTDEMRIAKEEIFGPVQQIMKFKSV  414

ALDH2_MOUSE    433  EEVVGRANDSKYGLAAAVFTKDLDKANYLSQALQAGTVWINCYDVFGAQSPFGG  486
ALDH1B1_MOUSE  433  EEVIQRANNTRYGLAAAVFTRDLDKAIYFTQALQAGTVWVNTYNIVTCHTPFGG  486
ALDH1A1_MOUSE  415  DDVIKRANNTTYGLAAGLFTKDLDKAITVSSALQAGVVWVNCYMMLSAQCPFGG  468

ALDH2_MOUSE    487  YKMSGSGRELGEYGLQAYTEVKTVTVKVPQKNS                      519
ALDH1B1_MOUSE  487  FKESGNGRELGEDGLRAYTEVKTVTIKVPEKNS                      519
ALDH1A1_MOUSE  469  FKMSGNGRELGEHGLYEYTELKTVAMKISQKNS                      501
```

Dotted line denotes mitochondrial targeting sequence

Green box denotes catalytic cysteine

Red box denotes amino acid mutated in the common human *ALDH2* polymorphism (E487K, *Aldh2*2*) responsible for the 'Asian Flush'

Figure S5

A

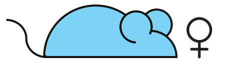

Recipient Female
Aldh2 +/+

P1 pups born from embryo transfer

	Aldh2 -/-			
	Fanca +/+	*Fanca +/-*	*Fanca -/-*	
F_{exp}	25 %	50 %	25 %	
F_{obs}	28.6 % n = 12	59.5 % n = 25	11.9 % n = 5	p = 0.24 ns

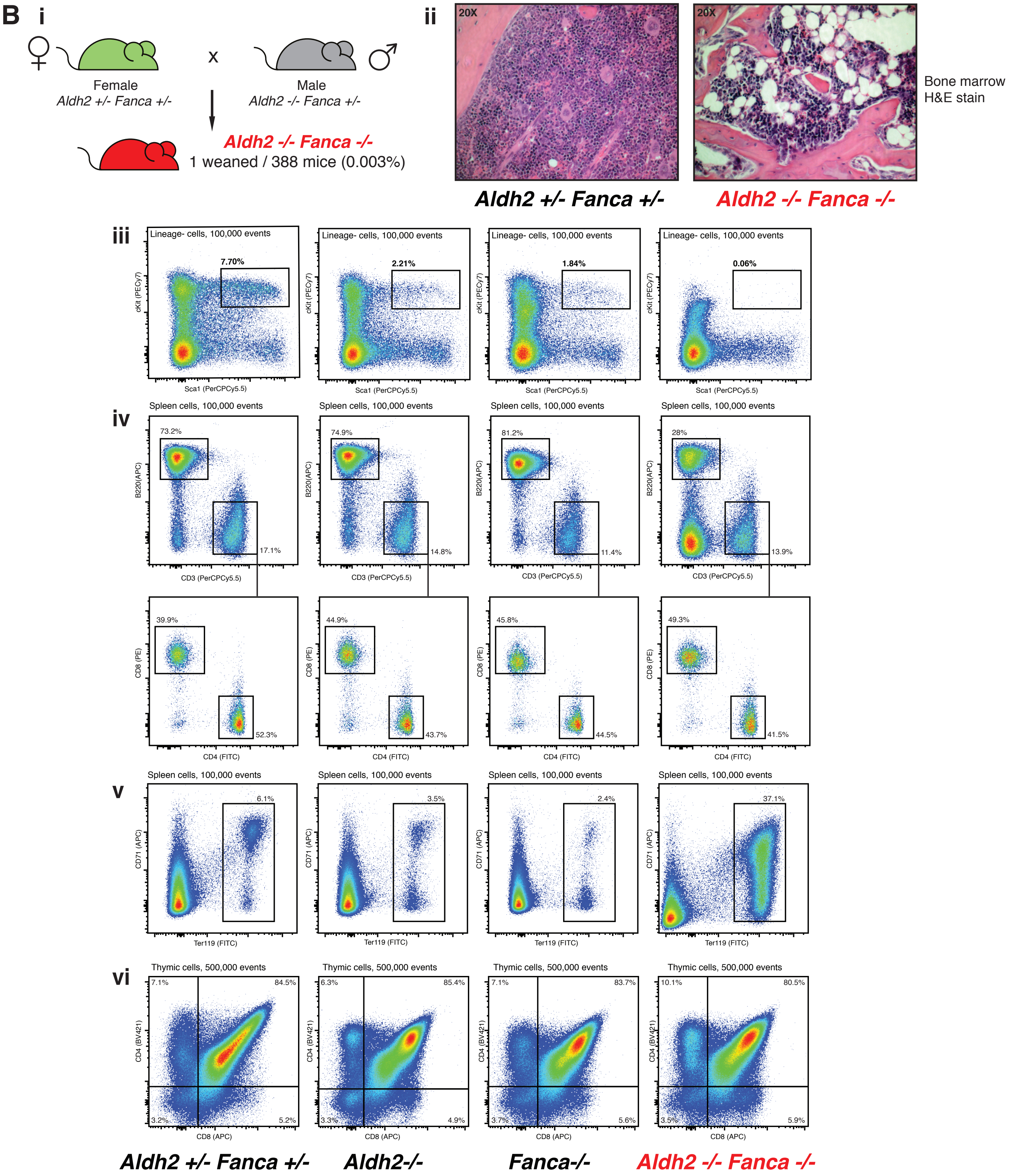

Figure S6

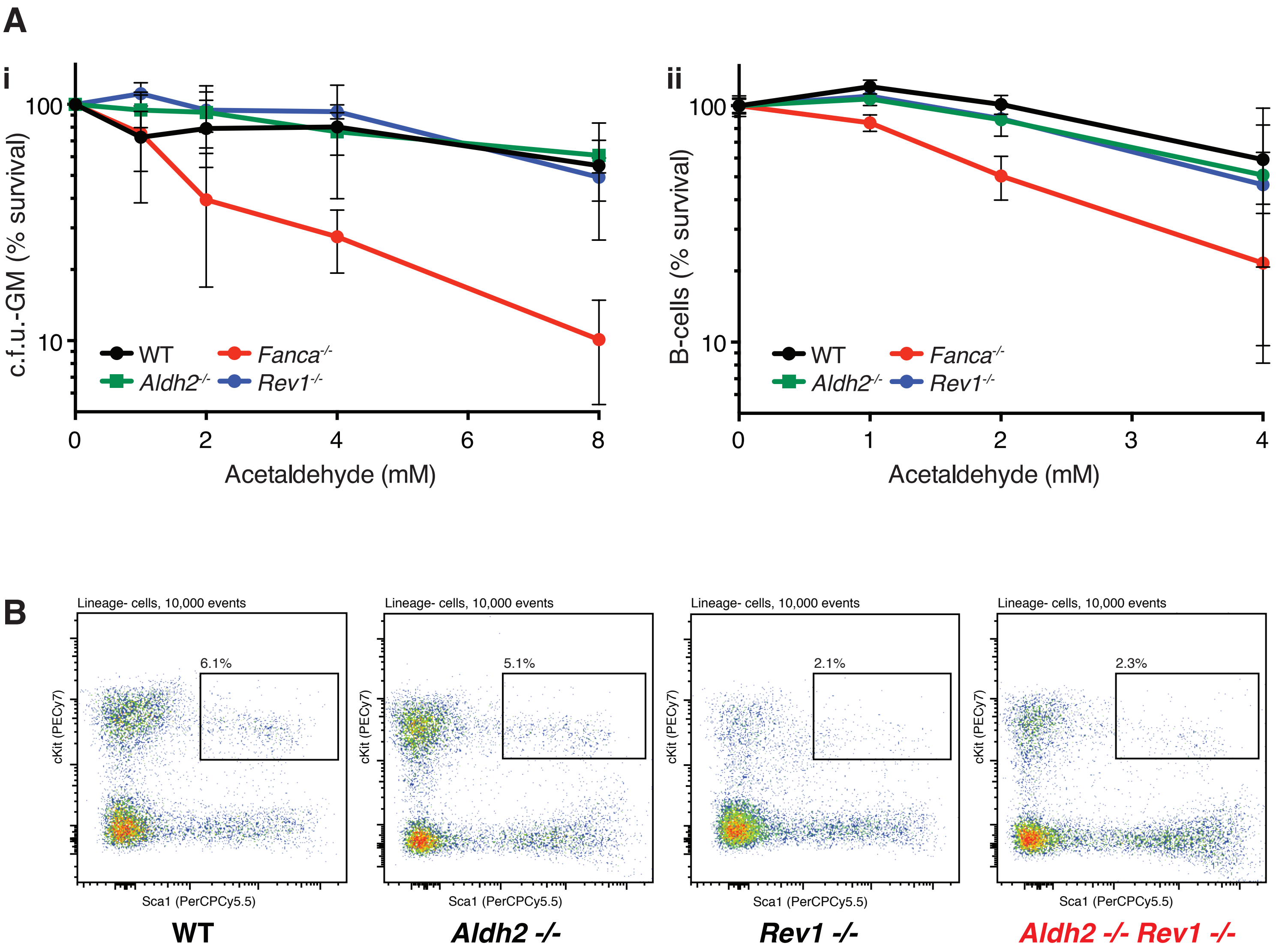

A
i
c.f.u.-GM (% survival)
100
10
WT
Fanca-/-
Aldh2-/-
Rev1-/-
0
2
4
6
8
Acetaldehyde (mM)
ii
B-cells (% survival)
100
10
WT
Fanca-/-
Aldh2-/-
Rev1-/-
0
1
2
3
4
Acetaldehyde (mM)
B
Lineage- cells, 10,000 events
6.1%
cKit (PECy7)
Sca1 (PerCPCy5.5)
WT
Lineage- cells, 10,000 events
5.1%
cKit (PECy7)
Sca1 (PerCPCy5.5)
Aldh2 -/-
Lineage- cells, 10,000 events
2.1%
cKit (PECy7)
Sca1 (PerCPCy5.5)
Rev1 -/-
Lineage- cells, 10,000 events
2.3%
cKit (PECy7)
Sca1 (PerCPCy5.5)
Aldh2 -/- Rev1 -/-

Supplemental Figure Legends

Figure S1, Related to Figure 1: *Aldh2$^{-/-}$Fanca$^{-/-}$* mice are embryonic lethal in the pure C57BL6/Jo1a genetic background

Observed and expected frequencies of all possible genotypes of offspring at **(A)** 3 weeks of age, **(B)** E16.5 and **(C)** E10.5-E12.5, obtained from the intercrosses displayed. The genotypes of interest: *Aldh2$^{+/-}$Fanca$^{+/-}$* and *Aldh2$^{-/-}$Fanca$^{-/-}$* are shaded in grey. Fisher's exact test, 5% confidence interval. For the data involving embryos, genotyped resorptions are not included in the data.

Figure S2, Related to Figure 2A and 2B: Description of the spontaneous developmental defects seen in E10.5-E12.5 embryos

(A) Bar graph displaying the proportion of developmentally compromised E10.5-E12.5 *Aldh2$^{+/-}$Fanca$^{-/-}$* and *Aldh2$^{-/-}$Fanca$^{-/-}$* embryos that have developmental defects only (purple), developmental delay only (orange), or have both developmental defects and delay (blue). Embryos were generated from either *Aldh2$^{-/-}$Fanca$^{+/-}$* (red mouse, left graph) or *Aldh2$^{+/-}$Fanca$^{+/-}$* (green mouse, right graph) females. The total number of embryos of each genotype is shown above each column.

(B) Pie chart displaying the percentage of developmentally compromised E10.5-E12.5 embryos of all genotypes that have developmental defects only (purple), developmental delay only (orange), or have both developmental defects and delay (blue). Bar graph adjacent to the pie chart shows the percentage of these embryos with eye, cranial or defects of other limbs, or that are pale. Embryos may, and often do, contain a combination of several of these defects. The embryos were generated from either *Aldh2$^{-/-}$Fanca$^{+/-}$* (red mouse, left charts) or *Aldh2$^{+/-}$Fanca$^{+/-}$* (green mouse, right charts) females.

(C) Representative images of developmentally compromised embryos at E10.5-E12.5 in comparison to littermate controls. This is to demonstrate the scoring of various developmental abnormalities. Red arrows denote eye defects. Black arrows denote cranial defects. Blue arrows denote limb defects.

Figure S3, Related to Figure 2B: Survival and eye defects in *Aldh2$^{+/-}$Fanca$^{-/-}$* mice

(A) Kaplan–Meier curve showing the survival of a cohort of *Aldh2$^{+/-}$Fanca$^{-/-}$* mice

born to Aldh2$^{+/+}$Fanca$^{+/-}$, *Aldh2*$^{+/-}$*Fanca*$^{-/-}$ and *Aldh2*$^{-/-}$*Fanca*$^{+/-}$ females, and congenic controls. *Aldh2*$^{+/-}$*Fanca*$^{-/-}$ mice born to Aldh2$^{-/-}$*Fanca*$^{+/-}$ mothers have severely reduced survival. The Gehan-Breslow-Wilcoxon test was used to compare survival curves.

(B) Bar graph showing the proportion of mice of each genotype which are normal (blue) or have eye defects (red). The total number of embryos of each genotype is shown above each column. Fisher's exact test was used to compare the proportion of embryos with eye defects between the different genotypes, 5% confidence interval.

Figure S4, Related to Figure 3: Protein alignment of the mouse Aldh2, Aldh1b1 and Aldh1a1 proteins

Three aldehyde dehydrogenases known to catabolize acetaldehyde are aligned using the T-Coffee alignment tool (EMBL-EBI). Dark blue denotes conserved residues. Aldh2 and Aldh1b1 contain a mitochondrial localisation signal.

Figure S5, Related to Figure 5

(A) Rescue of embryonic lethality when *Aldh2*$^{-/-}$*Fanca*$^{-/-}$ mice are carried by *Aldh2*$^{+/+}$ mothers

Observed and expected frequencies of P1 pups born from embryo transfer. Fisher's exact test, 5% confidence interval.

(B) A single *Aldh2*$^{-/-}$*Fanca*$^{-/-}$ mouse was born to an *Aldh2*$^{+/-}$*Fanca*$^{+/-}$ female and developed bone marrow failure at 7 weeks of age

(i) The genetic intercross which generated the single *Aldh2*$^{-/-}$*Fanca*$^{-/-}$ mouse.

(ii) Bone marrow histology of a control and the *Aldh2*$^{-/-}$*Fanca*$^{-/-}$ mouse, stained with haematoxylin and eosin (H&E; x20).

(iii) Flow cytometry profiles of bone marrow cells from the *Aldh2*$^{-/-}$*Fanca*$^{-/-}$ mouse and appropriate controls, showing 100,000 lineage-negative events. The *Aldh2*$^{-/-}$*Fanca*$^{-/-}$ mouse has a severely depleted Lin^{-}cKit^{+}Sca1^{+} (LKS) population.

(iv) Flow cytometry profiles of spleen cells from the *Aldh2*$^{-/-}$*Fanca*$^{-/-}$ mouse and appropriate controls, showing 100,000 cells. Cells were stained with B and T cell markers. The *Aldh2*$^{-/-}$*Fanca*$^{-/-}$ mouse has normal numbers of B and T cells in the spleen.

(v) Flow cytometry profiles of spleen cells from the *Aldh2*$^{-/-}$*Fanca*$^{-/-}$ mouse and

appropriate controls, showing 100,000 cells. Cells were stained with markers of erythropoiesis. The *Aldh2*$^{-/-}$*Fanca*$^{-/-}$ mouse shows extramedullary erythropoiesis.

(vi) Flow cytometry profiles of thymic cells from the *Aldh2*$^{-/-}$*Fanca*$^{-/-}$ mouse and appropriate controls, showing 500,000 cells. Cells were stained with markers of T-cells. The *Aldh2*$^{-/-}$*Fanca*$^{-/-}$ mouse has a normal T-cell profile.

Figure S6, Related to Figure 1 and Discussion

(A) *Rev1*$^{-/-}$ B cells and granulocyte-macrophage progenitors are not sensitive to exogenous acetaldehyde

Survival of WT, *Aldh2*$^{-/-}$, *Rev1*$^{-/-}$ and *Fanca*$^{-/-}$ **(i)** granulocyte–macrophage (c.f.u.-GM) progenitors or **(ii)** B cells following exposure to acetaldehyde *in vitro*. Each data point represents the mean of two independent experiments, each carried out in triplicate or quadruplicate respectively. Error bars represent SD.

(B) *Aldh2*$^{-/-}$*Rev1*$^{-/-}$ pups at P1 have an intact haematopoietic stem and progenitor cell (HSPC) population

Representative flow cytometry profiles of HSPCs of P1 pups, showing 10,000 lineage-negative events. The box denotes the Lin^{-}cKit^{+}Sca1^{+} (LKS) population. *Aldh2*$^{-/-}$ and *Aldh2*$^{-/-}$*Rev1*$^{-/-}$ pups were generated from intercrossing *Aldh2*$^{-/-}$*Rev1*$^{+/-}$ females and males. Wild type (WT) and *Rev1*$^{-/-}$ pups were generated from intercrossing *Rev1*$^{+/-}$ females and males.

Supplemental Experimental Procedures

Flow cytometry

Flow cytometry was performed on bone marrow, spleen and thymic cells that were isolated from mutant mice and appropriate controls. Bone marrow cells were obtained either by flushing from the femora and tibiae (adult bones) or by crushing the femora, tibiae and humeri (P1 pups) and passing through a 40-μm filter. The following antibodies were used to stain for the $Lin^{-}cKit^{+}Sca1^{+}$ population of the bone marrow: FITC-conjugated lineage cocktail with antibodies anti-CD4 (clone H129.19, BD Pharmingen), CD3e (clone 145-2C11, eBioscience), Ly-6G/Gr-1 (clone RB6-8C5, eBioscience), CD11b/Mac-1 (clone M1/70, BD Pharmingen), CD45R/B220 (clone RA3-6B2, BD Pharmingen), Fcε R1α (clone MAR-1, eBioscience), CD8a (clone 53-6.7, BD Pharmingen), CD11c (clone N418, eBioscience) and TER-119 (clone Ter119, BD Pharmingen) and anti-c-Kit (PerCP-Cy5.5, clone 2B8, eBioscience) and Sca-1 (PE-Cy7, clone D7, eBioscience) antibodies. B and T cells in the spleen were assessed using anti-CD45R/B220 (APC, clone RA3-6B2, BD Pharmingen), CD3e (PerCPCy5.5, clone 145-2C11, eBioscience), CD4 (FITC, clone H129.19, BD Pharmingen) and CD8a (PE, clone 53-6.7, BD Pharmingen) antibodies. The maturation of the erythroid lineage in the spleen was analysed using antibodies anti-TER-119 (FITC, clone Ter-119, BD Pharmingen) and anti-CD71 (APC, clone C2, BD Pharmingen). Thymic T-cells were assessed using a PE-conjugated lineage cocktail with antibodies anti-Ly-6G/Gr-1 (clone RB6-8C5, eBioscience), CD11b/Mac-1 (clone M1/70, BD Pharmingen), CD45R/B220 (clone RA3-6B2, BD Pharmingen), CD11c (clone N418, eBioscience), CD3e (clone 145-2C11, eBioscience) and TER-119 (clone Ter119, BD Pharmingen), and anti-CD4 (BV421, clone H129.19, BD Pharmingen) and CD8a (APC, clone 53-6.7, BD Pharmingen) antibodies. All antibodies were used at 1:200 and the samples were incubated for 15 min at 4 °C in the dark. Samples were run on a LSRII flow cytometer (BD Pharmingen) and the data were analysed with FlowJo 10.0.7 (Tree Star).

Aldehyde dehydrogenase activity assay

Mouse livers, whole E13.5 embryos or placentas (0.5 g) were finely minced with scissors and homogenised with 500 μl of homogenisation buffer (210 mM mannitol,

70 mM sucrose, 1 mM EDTA, 5 mM MOPS pH 7.4 in H_20) using a tight-fitting Dounce homogeniser. The homogenate was centrifuged at 700 x g for 10 min, the supernatant removed, and centrifuged again at 700 x g for 10 min. The supernatant was removed and centrifuged at 7000 x g for 20 min. The pellet was washed in homogenisation buffer and centrifuged again at 7000 x g for 10 min. The pellet was re-suspended in 300 µl of enzyme assay buffer (10 mM DTT, 20 % glycerol, 0.1 % Triton X-100 and 0.1 M Tris-HCl pH 8.0 in H_20) and centrifuged at 100,000 x g for 30 min at 4 °C to obtain a clear supernatant. Protein concentration was measured using a NanoDrop. To perform the Aldh enzymatic activity assay, a 2 ml reaction was set-up in a cuvette containing 50 mM NaPPi buffer pH 9.0, 2.5 mM NAD^+, 10 mM acetaldehyde and 0.5 mg protein preparation in H_20. The absorbance at 340 nm was recorded using a Cary 5000 UV-Vis-NIR spectrophotometer, at room temperature (RT) for 300 s without the addition of the acetaldehyde substrate, in order to quench the reaction of endogenous aldehydes. After 3 min, the acetaldehyde was added, and the absorbance at 340 nm was recorded for a further 350 s. To calculate the [NADH] production in mols/min/mg total protein, we used: Absorbance = ε x c x L, where ε = 6220 M^{-1}, L = path length (1 cm) and c = [NADH] in mols. The assay was adapted from a protocol by D Mochley-Rosen, Stanford University.

Survival assays of primary mouse B cells

The primary mouse B cells survival assay was performed with lymphocytes purified from the spleen using Lympholyte M (Cederlane). Lymphocytes were stimulated with LPS (Sigma L4391) at a final concentration of 40 µg ml^{-1}. A total of 4×10^5 cells were plated with acetaldehyde at various concentrations in one well of a 24-well plate. After 7 days, the total cells were enumerated, counting 100 images using a ViCell XR (Beckman Coulter). Each data point represents the mean of two independent experiments, each carried out in quadruplicate.

Methylcellulose c.f.u.-GM survival assay

The methylcellulose c.f.u.-GM survival assay was carried out as described previously (Meagher et al., 1982). Briefly, total bone marrow was flushed in IMDM (GIBCO) from the femora and tibiae of mice. Equal numbers of total bone marrow cells were exposed to various concentrations of acetaldehyde *in vitro* for 4 h in a sealed CryoVial. After treatment, two ten-fold serial dilutions of the bone marrow were

made, and these cells were plated in duplicate into 6-well plates with methylcellulose medium with recombinant cytokines (without EPO; MethoCult™ GF M3534). Colonies were counted after 7 days.

Supplemental References

Meagher, R.C., Sieber, F., and Spivak, J.L. (1982). Suppression of Hematopoietic-Progenitor-Cell Proliferation by Ethanol and Acetaldehyde. N. Engl. J. Med. *307*, 845-849.
