# Supplementary material for: Maternal Aldehyde Elimination during Pregnancy Preserves the Fetal Genome
Source: Mol Cell. 2014 Sep 18;55(6):807–17. doi: 10.1016/j.molcel.2014.07.010 (PMC4175174; doi:10.1016/j.molcel.2014.07.010)
Supplement: Document S2. Article plus Supplemental Information [file mmc2.pdf]

CellPress

Maternal Aldehyde Elimination during Pregnancy Preserves the Fetal Genome

Nina Oberbeck,[1] Frédéric Langevin,[1] Gareth King,[1] Niels de Wind,[2] Gerry P. Crossan,[1,4,*] and Ketan J. Patel[1,3,4,*]
[1]MRC Laboratory of Molecular Biology, Francis Crick Avenue, Cambridge CB2 0QH, UK
[2]Department of Toxicogenetics, Leiden University Medical Center, P.O. Box 9600, 2300 RC Leiden, the Netherlands
[3]Department of Medicine, Level 5, Addenbrooke's Hospital, University of Cambridge, Cambridge CB2 0QQ, UK
[4]Co-senior author
*Correspondence: gcrossan@mrc-lmb.cam.ac.uk (G.P.C.), kjp@mrc-lmb.cam.ac.uk (K.J.P.)
http://dx.doi.org/10.1016/j.molcel.2014.07.010
This is an open access article under the CC BY license (http://creativecommons.org/licenses/by/3.0/).

SUMMARY

Maternal metabolism provides essential nutrients to enable embryonic development. However, both mother and embryo produce reactive metabolites that can damage DNA. Here we discover how the embryo is protected from these genotoxins. Pregnant mice lacking Aldh2, a key enzyme that detoxifies reactive aldehydes, cannot support the development of embryos lacking the Fanconi anemia DNA repair pathway gene *Fanca*. Remarkably, transferring *Aldh2$^{-/-}$Fanca$^{-/-}$* embryos into wild-type mothers suppresses developmental defects and rescues embryonic lethality. These rescued neonates have severely depleted hematopoietic stem and progenitor cells, indicating that despite intact maternal aldehyde catabolism, fetal Aldh2 is essential for hematopoiesis. Hence, maternal and fetal aldehyde detoxification protects the developing embryo from DNA damage. Failure of this genome preservation mechanism might explain why birth defects and bone marrow failure occur in Fanconi anemia, and may have implications for fetal well-being in the many women in Southeast Asia that are genetically deficient in *ALDH2*.

INTRODUCTION

Birth defects are common and a substantial burden to human health, but their etiology is complex and often due to many factors. Maternal exposure to X-rays and chemotherapeutic agents give rise to birth defects, mainly because these agents cause direct damage to the fetal genome (Arnon et al., 2001; Hall, 1991; Streffer et al., 2003; Toledo et al., 1971). The developing embryo limits this damage through DNA repair, thus attenuating the potential of these mutagens to corrupt development. This is further underscored by striking developmental phenotypes associated with humans that are genetically defective in certain DNA repair pathways.

Children afflicted with Fanconi anemia (FA) have a genetic deficiency in DNA repair and are often born with a multitude of birth defects affecting many organs, combined with intrauterine growth retardation (Alter and Rosenberg, 2013). Provided these defects are not so severe as to cause early death, most FA patients develop bone marrow failure and are cancer prone (Alter, 2003; Kutler et al., 2003). FA results from an inability to repair DNA crosslinks, a specific form of DNA damage where the two complementary strands of DNA are covalently linked. This disease is genetically heterogeneous with germline mutations in any one of sixteen genes (*FANCA-Q*) resulting in the disease (Garaycoechea and Patel, 2014). Despite the significant progress made in identifying the genes responsible for FA and defining how their gene products cooperate to repair DNA crosslinked by chemotherapeutic agents (such as cisplatin), our understanding of how this DNA repair defect leads to congenital abnormalities and bone marrow failure is limited (Hodskinson et al., 2014; Klein Douwel et al., 2014; Knipscheer et al., 2009; Räschle et al., 2008). In addition, whilst the developmental defects associated with FA clearly relate to a fundamental role for DNA crosslink repair during embryogenesis, it is unclear how the intrauterine environment contributes to the eventual loss of bone marrow function.

We recently showed that a major physiological function of the FA DNA repair pathway is to protect the genome from damage caused by endogenous aldehydes (Garaycoechea et al., 2012; Langevin et al., 2011; Rosado et al., 2011). These highly reactive molecules are byproducts of many metabolic pathways, such as lipid peroxidation and the breakdown of alcohols. Mice that lack both the aldehyde-detoxifying enzyme Aldh2 and the key FA protein Fancd2 are cancer prone and develop bone marrow failure (Garaycoechea et al., 2012; Langevin et al., 2011). Here we address whether metabolically derived aldehydes cause DNA damage to the developing embryo. Our results reveal how maternal and fetal aldehyde catabolism cooperate with fetal FA DNA repair to preserve development.

RESULTS

A Role for *Aldh2* and Only Certain DNA Crosslink Repair Genes in Development

Aldh2$^{-/-}$Fancd2$^{-/-}$ mice generated in a hybrid genetic background (C57BL6/Jo1a × 129S6/SvTac) succumb to leukemia and possess very few hematopoietic stem cells (HSCs). These double-deficient mice cannot be born to *Aldh2$^{-/-}$* mothers but

CrossMark

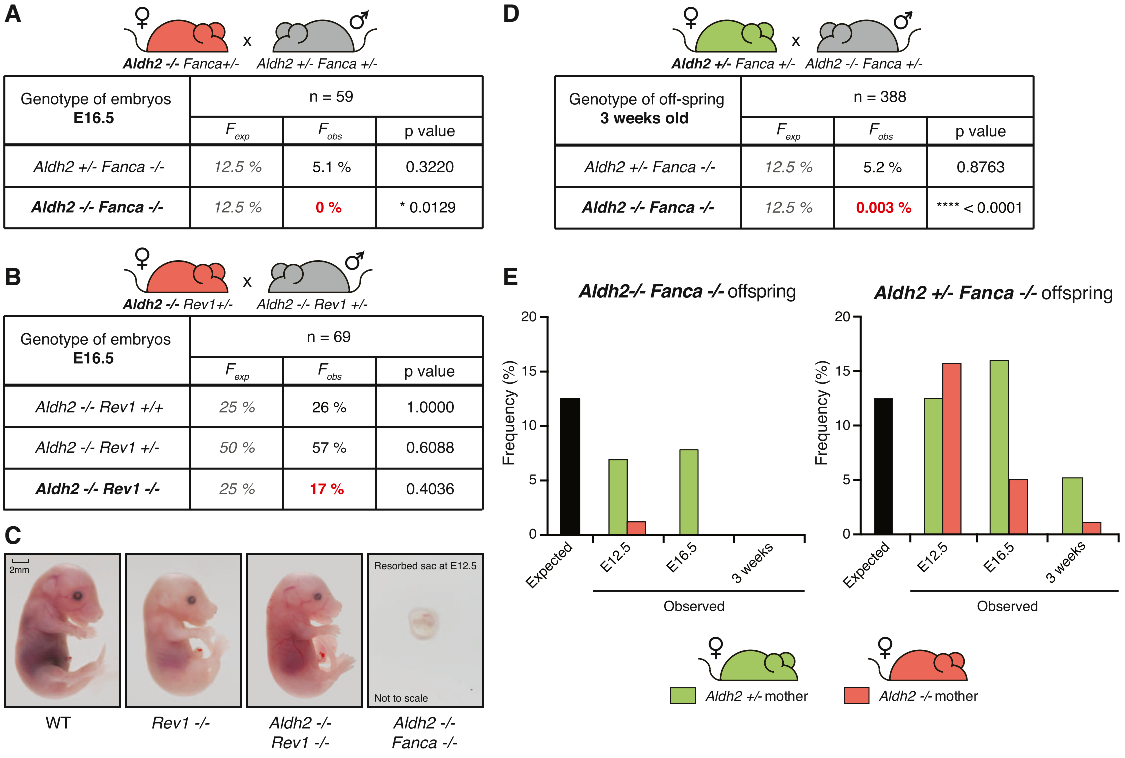

Figure 1. *Aldh2* Is Essential for the Development of *Fanca*$^{-/-}$, but Not *Rev1*$^{-/-}$, Embryos

(A) Observed and expected frequencies of *Aldh2*$^{+/-}$*Fanca*$^{-/-}$ and *Aldh2*$^{-/-}$*Fanca*$^{-/-}$ fetuses at E16.5, obtained from *Aldh2*$^{-/-}$*Fanca*$^{+/-}$ females (red) intercrossed with *Aldh2*$^{+/-}$*Fanca*$^{+/-}$ males (gray). Genotyped resorptions were not included in the data. Fisher's exact test, 5% confidence interval.

(B) Observed and expected frequencies of *Aldh2*$^{-/-}$*Rev1*$^{+/+}$, *Aldh2*$^{-/-}$*Rev1*$^{+/-}$, and *Aldh2*$^{-/-}$*Rev1*$^{-/-}$ fetuses at E16.5, obtained from intercrossing *Aldh2*$^{-/-}$*Rev1*$^{+/-}$ females (red) and males (gray). Genotyped resorptions were not included in the data. Fisher's exact test, 5% confidence interval.

(C) Representative images of E16.5 fetuses of various genotypes referred to in the table in (B). The *Aldh2*$^{-/-}$*Rev1*$^{-/-}$ and *Aldh2*$^{-/-}$*Fanca*$^{-/-}$ embryos were both generated from *Aldh2*$^{-/-}$ females. The *Aldh2*$^{-/-}$*Fanca*$^{-/-}$ resorbed sac was at E12.5.

(D) Observed and expected frequencies of *Aldh2*$^{+/-}$*Fanca*$^{-/-}$ and *Aldh2*$^{-/-}$*Fanca*$^{-/-}$ mice at 3 weeks of age, obtained from *Aldh2*$^{+/-}$*Fanca*$^{+/-}$ females (green) intercrossed with *Aldh2*$^{-/-}$*Fanca*$^{+/-}$ males (gray). Fisher's exact test, 5% confidence interval.

(E) Bar graphs show frequencies of *Aldh2*$^{-/-}$*Fanca*$^{-/-}$ and *Aldh2*$^{+/-}$*Fanca*$^{-/-}$ offspring at E10.5–E12.5, E16.5, and 3 weeks of age, generated from either *Aldh2*$^{+/-}$*Fanca*$^{+/-}$ females (green bars) or *Aldh2*$^{-/-}$*Fanca*$^{+/-}$ females (red bars). These females were intercrossed with *Aldh2*$^{-/-}$*Fanca*$^{+/-}$ and *Aldh2*$^{+/-}$*Fanca*$^{+/-}$ males, respectively. The expected frequency is represented by the black bar (12.5%). Genotyped resorptions are not included in the data. Refer to Figure S1 for complete data and statistics.

can be born to mothers that are heterozygous for *Aldh2* (*Aldh2*$^{+/-}$), intimating a crucial developmental role for maternal aldehyde catabolism (Langevin et al., 2011). In order to understand this essential maternal requirement for *Aldh2* in the development of DNA repair-deficient embryos, we initially set out to establish three key points: (1) what contribution the mouse genetic background might have on this phenomenon, (2) whether the genetic requirement for DNA repair was generalizable to a FA repair gene upstream of *Fancd2*, and (3) if this interaction was a feature of DNA crosslink repair generally or restricted only to the FA repair genes.

Autosomal recessive mutations in any one of sixteen genes (*FANCA-Q*) can be mutated in FA, but almost 60% of the cases are due to mutations in the *FANCA* gene (Neveling et al., 2009). We therefore set out to generate mice that lack *Aldh2* and *Fanca* (*Aldh2*$^{-/-}$*Fanca*$^{-/-}$) in a pure C57BL6/Jo1a background. In the first instance, we mated *Aldh2*$^{-/-}$*Fanca*$^{+/-}$ females with *Aldh2*$^{+/-}$*Fanca*$^{+/-}$ males and found that no *Aldh2*$^{-/-}$*Fanca*$^{-/-}$ mice were weaned (Figure S1A, left table, available online). This result indicates that *Aldh2* deficiency is synthetically lethal with *Fanca* deficiency, similar to what has already been reported for *Aldh2* and *Fancd2* in the hybrid background. To learn more about the timing of this synthetic lethal interaction, we looked earlier in development, at embryonic day 16.5 (E16.5), and found that *Aldh2*$^{-/-}$*Fanca*$^{-/-}$ fetuses were already absent in late gestation (Figure 1A).

Genetic and biochemical evidence has shown that DNA translesion synthesis (TLS) mediated by Rev1 is essential for DNA crosslink repair. Moreover, Rev1 and the FA proteins function in a common pathway to maintain resistance to DNA interstrand crosslinking agents (Niedzwiedz et al., 2004; Ross et al., 2005). Mice lacking *Rev1* are born at lower than expected Mendelian frequency, and their postnatal life is very similar to, or even slightly more severe than that of FA knockout mice (Jansen et al., 2006). We therefore asked if *Aldh2*$^{-/-}$*Rev1*$^{-/-}$ embryos were viable in late gestation when conceived by *Aldh2*$^{-/-}$

mothers. Surprisingly, *Aldh2*$^{-/-}$*Rev1*$^{-/-}$ E16.5 fetuses are readily detected and appear developmentally intact and similar to *Rev1*$^{-/-}$ or wild-type controls (Figures 1B and 1C). This is in stark contrast to *Aldh2*$^{-/-}$*Fanca*$^{-/-}$ embryos, which are completely resorbed by E12.5. We finally asked if a single maternal allele of *Aldh2* would enable *Aldh2*$^{-/-}$*Fanca*$^{-/-}$ mice to be born. This was particularly important since *Aldh2*$^{-/-}$*Fancd2*$^{-/-}$ mice bred in the C57BL6/Jo1a × 129S6/SvTac background are viable only when conceived by *Aldh2*$^{+/-}$ mothers. Surprisingly, *Aldh2*$^{-/-}$*Fanca*$^{-/-}$ mice in the pure C57BL6/Jo1a background are not viable, even when generated from the same cross that allowed the birth of *Aldh2*$^{-/-}$*Fancd2*$^{-/-}$ mice (Figure 1D). We also noted that *Aldh2*$^{+/-}$*Fanca*$^{-/-}$ mice are not born at the expected ratio. When *Aldh2*$^{+/-}$*Fanca*$^{-/-}$ pups are conceived by *Aldh2*$^{-/-}$*Fanca*$^{+/-}$ mothers, they are observed at a reduced frequency of 1.1% by 3 weeks (expected 12.5%, *p = 0.0114). This is mirrored in *Aldh2*$^{+/-}$*Fanca*$^{-/-}$ mothers, where there is a tendency toward reduced numbers of *Aldh2*$^{+/-}$*Fanca*$^{-/-}$ pups (ns, p = 0.8763). These data demonstrate a fetal Aldh2 haploinsufficient effect by which one fetal allele of *Aldh2* is insufficient to fully rescue the embryonic lethality of *Fanca*$^{-/-}$ embryos (Figures S1A and 1D).

We then determined the point at which *Aldh2*$^{-/-}$*Fanca*$^{-/-}$ and *Aldh2*$^{+/-}$*Fanca*$^{-/-}$ embryos died during gestation, from either *Aldh2*$^{-/-}$ or *Aldh2*$^{+/-}$ mothers (Figures 1E, S1A, S1B, and S1C). This revealed that *Aldh2*$^{-/-}$ mothers resorb *Aldh2*$^{-/-}$*Fanca*$^{-/-}$ embryos early in gestation, prior to E12.5, but these embryos survive longer in *Aldh2*$^{+/-}$ mothers, with fetuses being evident at E16.5. We also noted that a single allele of fetal *Aldh2* enables *Fanca*$^{-/-}$ embryos to proceed further in gestation, but their viability is also impacted by maternal aldehyde catabolism. This indicates that although the maternal *Aldh2* status has a profound influence on development, fetal *Aldh2* also provides protection. In summary, maternal and fetal Aldh2 is essential for the viability of *Fanca*$^{-/-}$ embryos, but surprisingly this is not the case for *Rev1*$^{-/-}$ embryos, in a directly comparable pure C57BL6/Jo1a genetic background.

Maternal *Aldh2* Is Critical for the Development of *Fanca*-Deficient Embryos and in Their Protection against Exogenous Ethanol

We next sought to comprehensively analyze the manner in which the development of *Aldh2*$^{-/-}$*Fanca*$^{-/-}$ and *Aldh2*$^{+/-}$*Fanca*$^{-/-}$ embryos is impacted when they are conceived by either *Aldh2*$^{-/-}$ (red) or *Aldh2*$^{+/-}$ (green) mothers (Figure 2A). We quantified the data in Figure 2A by genotyping embryos conceived by the two maternal genotypes and scoring them into three groups (normal, blue; embryos that were delayed in development or carried anatomical defects, green; embryos that were resorbed, red; Figure 2B). Representative images of the embryos of the three main genotypes in Figure 2A show first that *Aldh2*$^{-/-}$*Fanca*$^{-/-}$ embryos are dying from a widespread failure in development. When conceived by *Aldh2*$^{-/-}$ mothers, double mutants are mostly resorbed by E12.5. However, when conceived by *Aldh2*$^{+/-}$ mothers, double mutants are still intact at E12.5, but all have developmental defects including eye, limb, and craniofacial abnormalities (Figures 2B, S2A, and S2B). Similarly, *Aldh2*$^{+/-}$*Fanca*$^{-/-}$ embryos, when conceived by *Aldh2*$^{-/-}$ mothers, are resorbed and show developmental defects, and a single maternal allele of *Aldh2* suppresses these resorptions (Figures 2B, S2A, and S2B). There is a wide spectrum of developmental abnormalities affecting multiple organ systems, which may reflect the stochastic nature of DNA damage (Figures S2C and S2D). *Aldh2*$^{+/-}$*Fanca*$^{-/-}$ mice born to *Aldh2*$^{-/-}$ mothers are rare; however, they can be born to *Aldh2*$^{+/-}$ mothers (albeit at a lower Mendelian frequency than expected), and they have a similar survival to *Fanca*$^{-/-}$ mice. The Kaplan-Meier curve demonstrates that *Aldh2*$^{+/-}$*Fanca*$^{-/-}$ mice born to *Aldh2*$^{-/-}$ mothers have a severely reduced survival compared to the same mice born to Aldh2-proficient mothers (Figure S3). In summary, these data highlight that maternal Aldh2 deficiency has a profound impact on FA-deficient embryos. This is best illustrated by the fact that the presence of just one maternal allele of *Aldh2* almost completely suppresses resorption of *Aldh2*$^{-/-}$*Fanca*$^{-/-}$ embryos at E12.5 (green mouse symbol, Figure 2B).

The origin, identity, and sites of clearance of the physiologically relevant toxic aldehyde(s) within the mother are unclear. Ethanol is an important source of acetaldehyde, which is the key substrate for Aldh2 and causes developmental failure in fetal alcohol syndrome (O'Shea and Kaufman, 1979; Webster et al., 1983). We therefore reasoned that challenging mothers carrying *Fanca*$^{-/-}$ embryos with ethanol should provide direct evidence for an aldehyde causing damage to the embryos. When *Aldh2*$^{+/-}$ mothers are exposed to an acute dose of ethanol (4.5 g/kg) during early pregnancy (E7.5), all *Aldh2*$^{+/-}$*Fanca*$^{-/-}$ embryos are developmentally compromised (this is not the case in the absence of ethanol; Figures 2C and 2D). We next asked whether an extra maternal allele of *Aldh2* could protect these embryos from ethanol-derived aldehyde-mediated damage. When *Aldh2*$^{+/+}$ mothers were given exogenous ethanol during gestation, *Aldh2*$^{+/-}$*Fanca*$^{-/-}$ embryos remained developmentally intact and were significantly protected, in strong contrast to the same embryos from an *Aldh2*$^{+/-}$ mother (Figures 2D and 2E). The striking effect of maternal ethanol challenge clearly demonstrates that maternal haploinsufficiency leads to reduced fetal protection to aldehydes. Cumulatively, these results show the extent to which maternal aldehyde catabolism deficiency impacts the development of *Aldh2*$^{-/-}$*Fanca*$^{-/-}$ and *Aldh2*$^{+/-}$*Fanca*$^{-/-}$ embryos.

Expression of Acetaldehyde-Catabolizing Enzymes during Pregnancy

Given the importance of maternal aldehyde catabolism for the development of *Fanca*$^{-/-}$ embryos, we next wanted to determine where aldehyde catabolism occurs within the fetal-maternal unit. To address this, we looked at the expression of Aldh2 and two closely related aldehyde dehydrogenases, Aldh1a1 and Aldh1b1, in the mother and embryo (Figure S4). These two enzymes are known to also catabolize acetaldehyde (Stagos et al., 2010; Yoshida et al., 1992). The maternal liver expresses all three Aldh enzymes at high levels, with expression also present in most maternal tissues (Figure 3A). However, it came as a particular surprise that the placenta shows no detectable Aldh2, Aldh1a1, and Aldh1b1 expression (Figure 3B); this organ is the interface between the maternal and fetal circulation. In addition, whole E12.5 embryos show very low levels of Aldh2

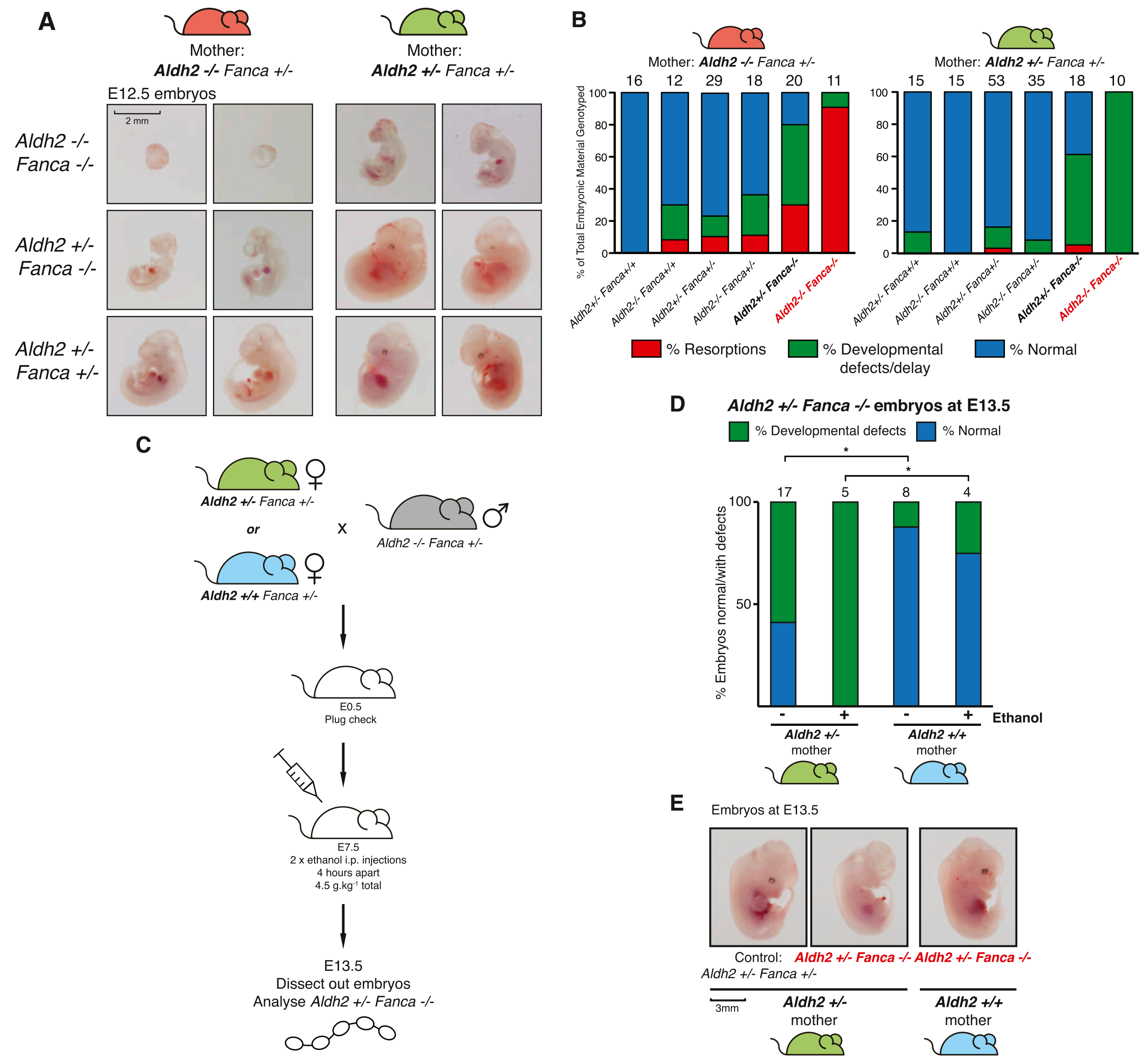

Figure 2. Nature of Spontaneous and Ethanol-Induced Developmental Defects in *Fanca*$^{-/-}$ Embryos in Relation to Maternal *Aldh2*

(A) Representative images of *Aldh2*$^{-/-}$*Fanca*$^{-/-}$ and *Aldh2*$^{+/-}$*Fanca*$^{-/-}$ embryos, in comparison to *Aldh2*$^{+/-}$*Fanca*$^{+/-}$ controls, at E12.5, generated from either *Aldh2*$^{-/-}$*Fanca*$^{+/-}$ (red mouse, left) or *Aldh2*$^{+/-}$*Fanca*$^{+/-}$ (green mouse, right) females.

(B) Bar graph displaying the proportion of E10.5–E12.5 embryos that are normal (blue), have developmental defects/delay (green), and are resorptions (red) out of the total embryonic material genotyped. Embryos were generated from either *Aldh2*$^{-/-}$*Fanca*$^{+/-}$ (red mouse, left graph) or *Aldh2*$^{+/-}$*Fanca*$^{+/-}$ (green mouse, right graph) females. The total number of embryos of each genotype is shown above each column.

(C) Schematic representation of the experiment to expose pregnant *Aldh2*$^{+/+}$*Fanca*$^{+/-}$ or *Aldh2*$^{+/-}$*Fanca*$^{+/-}$ females to ethanol.

(D) Bar graph displaying the proportion of *Aldh2*$^{+/-}$*Fanca*$^{-/-}$ E13.5 embryos that are normal (blue) and have developmental defects (green). These embryos were generated from either *Aldh2*$^{+/-}$*Fanca*$^{+/-}$ females (green mouse) or *Aldh2*$^{+/+}$*Fanca*$^{+/-}$ females (blue mouse) who had either been exposed to ethanol at E7.5 of pregnancy or not. Genotyped resorptions were not included in the data. Fisher's exact test was used to compare the proportion of embryos with developmental defects between *Aldh2*$^{+/-}$ and *Aldh2*$^{+/+}$ mothers; *$p < 0.05$, 5% confidence interval. The total number of embryos of each genotype is shown above each column.

(E) Representative images of control and *Aldh2*$^{+/-}$*Fanca*$^{-/-}$ E13.5 embryos, generated from either an *Aldh2*$^{+/-}$*Fanca*$^{+/-}$ female (green mouse) or an *Aldh2*$^{+/+-}$ *Fanca*$^{+/-}$ female (blue mouse), following exposure to ethanol during pregnancy.

protein, a striking difference to that observed for the maternal liver. It is possible that another Aldh-class enzyme might be expressed in the placenta and the embryo. To address this, we carried out an enzymatic assay for the catabolism of acetaldehyde (Figure 3C). We used acetaldehyde, as it is a known substrate of Aldh2, Aldh1b1, and Aldh1a1. In addition, the ethanol challenge experiment described above shows that acetaldehyde can damage *Aldh2*$^{+/-}$*Fanca*$^{-/-}$ embryos conceived by *Aldh2*$^{+/-}$ mothers. This assay measures the turnover of NAD^+ (an essential cofactor for aldehyde dehydrogenases) in crude mitochondrial extracts

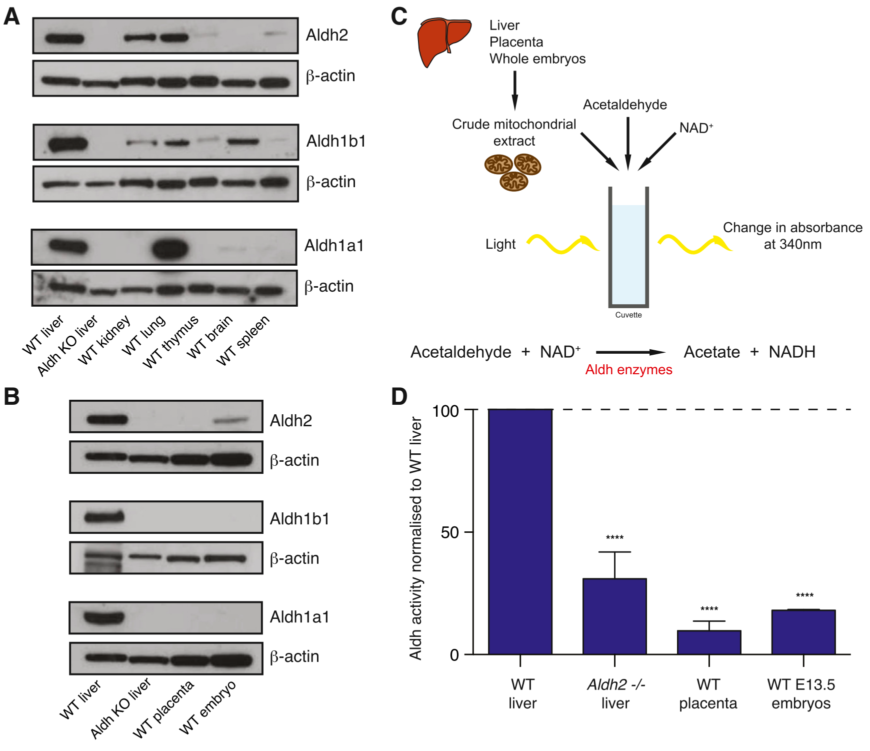

Figure 3. Expression and Activity of Acetaldehyde-Catabolizing Enzymes in the Fetal-Maternal Unit

(A) Western blot showing the expression of Aldh2, Aldh1b1, and Aldh1a1 in whole-cell extracts of a panel of tissues taken from a wild-type mouse. Expression of beta-actin is shown as a loading control. Whole-cell extracts from the corresponding livers obtained from *Aldh* knockout females is shown as a control for the specificity of each antibody.

(B) Western blot showing the expression of Aldh2, Aldh1b1, and Aldh1a1 in whole-cell extracts of wild-type (WT) liver, placenta, and E12.5 embryo. Expression of beta-actin is shown as a loading control. The corresponding *Aldh* knockout in the liver is again run alongside tissue samples to control for the specificity of each antibody.

(C) Schematic of the Aldh activity assay. Acetaldehyde is converted to acetate by Aldh2 (and other Aldh enzymes), which produces NADH. The production of NADH, and thus the Aldh activity, can be measured in a crude mitochondrial extract by the addition of acetaldehyde and assessment of the rate of change of absorbance at 340 nm.

(D) Aldh2 activity in the mouse wild-type (WT) liver, *Aldh2*$^{-/-}$ liver, WT placenta, and WT E13.5 embryo. Aldh activities were normalized to WT liver. Unpaired t test, ****$p < 0.0001$, 5% confidence interval. Error bars represent SD. n = 7 for WT and *Aldh2*$^{-/-}$ liver, n = 3 for placenta, n = 2 for embryos.

incubated with acetaldehyde. Maternal liver extract shows very robust acetaldehyde catabolism that is greatly reduced in an *Aldh2*$^{-/-}$ liver extract (Figure 3D). Activity in the *Aldh2*$^{-/-}$ liver is not completely absent due to redundant mitochondrial and contaminating cytosolic enzymes. The aldehyde catabolism activity in the placenta and E13.5 embryo extract is low; in fact, it is similar to that of an *Aldh2*$^{-/-}$ liver. This indicates that the fetus and placenta may not be able to contribute to aldehyde detoxification to the same extent as that of the mother in early development.

Aldh2 Deficiency Leads to the Accumulation of DNA Damage in *Fanca*$^{-/-}$ Embryos

The striking developmental phenotype of *Aldh2*$^{-/-}$*Fanca*$^{-/-}$ and *Aldh2*$^{+/-}$*Fanca*$^{-/-}$ embryos when conceived by either *Aldh2*$^{-/-}$ or *Aldh2*$^{+/-}$ mothers led us to ask whether this correlated with an accumulation of DNA damage. DNA double-strand breaks (DSBs) in the nucleus stimulate the phosphorylation of histone H2AX (γH2AX), which is readily detected by western blot analysis (Rogakou et al., 1998). We therefore made whole-cell extracts from E12.5 embryos generated from both *Aldh2*$^{+/-}$ and *Aldh2*$^{-/-}$ mothers. Strikingly, *Aldh2*$^{-/-}$*Fanca*$^{-/-}$ and *Aldh2*$^{+/-}$*Fanca*$^{-/-}$ embryos can be clearly distinguished from their littermates through the strong presence of γH2AX, a marker of DNA damage (Figures 4A and 4B). While *Aldh2*$^{+/+}$*Fanca*$^{-/-}$ control embryos do show a γH2AX signal, this is far less striking than the induction observed for *Aldh2*$^{-/-}$*Fanca*$^{-/-}$ embryos (Figure 4C). In addition, *Aldh2*$^{-/-}$*Fanca*$^{-/-}$ embryos have the most γH2AX compared to *Aldh2*$^{+/-}$*Fanca*$^{-/-}$ and *Fanca*$^{-/-}$ embryos (Figure 4D).

We also looked at the induction of γH2AX and cleaved caspase-3 (a marker of apoptosis) by immunohistochemistry. We stained sections from the periventricular region of developing brain of E12.5 embryos (conceived by an *Aldh2*$^{+/-}$ mother). This is an easily identifiable region of the brain of the embryo, with a defined anatomy, which allows direct comparison between genotypes. It is immediately apparent that many more cells in this region of *Aldh2*$^{-/-}$*Fanca*$^{-/-}$ embryos stain strongly for nuclear γH2AX and cleaved capase-3 versus control (Figure 4E). These results indicate that deficiency of maternal and fetal aldehyde catabolism results in the accumulation of DNA damage and increased apoptosis in *Fanca*-deficient embryos.

Rescue of Embryonic Lethality, but Not Hematopoietic Stem Cells, when *Aldh2*$^{-/-}$*Fanca*$^{-/-}$ Mice Are Carried by *Aldh2*$^{+/+}$ Mothers

So far we have shown that the maternal capacity to remove aldehydes protects the embryo from accumulating endogenous DNA damage and developmental defects. Although this maternal detoxification is important, we also know that fetal Aldh2 and DNA repair provide protection. In order to disassociate how much maternal and fetal aldehyde catabolism contributes to ensure development, we sought to determine if complete aldehyde detoxification by the mother could rescue developmental attrition of *Aldh2*$^{-/-}$*Fanca*$^{-/-}$ embryos, using embryo transfer experiments (as natural genetic crosses do not allow us to address this question). Embryos at the two-cell stage obtained from crossing *Aldh2*$^{-/-}$*Fanca*$^{+/-}$ males and females were harvested and transferred into the uteri of mothers completely competent at aldehyde catabolism (*Aldh2*$^{+/+}$; Figure 5A). This

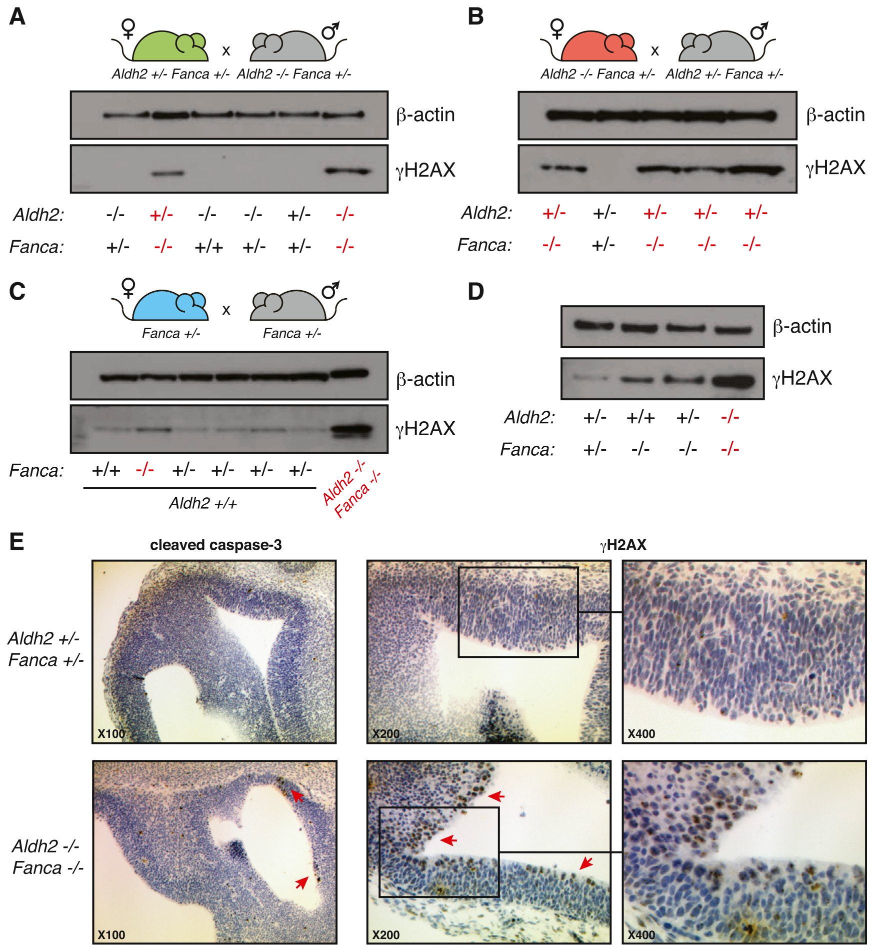

Figure 4. Aldehyde Catabolism Prevents DNA Damage in *Fanca*-Deficient Embryos

(A) Western blot showing the detection of γH2AX in whole-cell extracts made from individual whole E12.5 embryos (where the fetal livers had been removed). Individual embryos from a single litter, generated from the mating displayed, are shown. *Aldh2*$^{-/-}$*Fanca*$^{-/-}$ and *Aldh2*$^{+/-}$*Fanca*$^{-/-}$ embryos can be clearly distinguished from littermates through the presence of γH2AX. Expression of beta-actin is shown as a loading control.

(B) Western blot showing the detection of γH2AX in whole-cell extracts made from individual whole E12.5 embryos (where the fetal livers had been removed). Individual embryos from a single litter, generated from the mating displayed, are shown. *Aldh2*$^{+/-}$*Fanca*$^{-/-}$ embryos can be clearly distinguished from littermates through the presence of γH2AX. Expression of beta-actin is shown as a loading control.

(C) Western blot showing the detection of γH2AX in whole-cell extracts made from individual whole E12.5 embryos (where the fetal livers had been removed). Individual embryos from a single litter, generated from the mating displayed, are shown. Expression of beta-actin is shown as a loading control.

(D) Western blot comparing the relative detection of γH2AX in whole-cell extracts made from individual whole E12.5 embryos of the relevant genotypes (not all from the same litter). Expression of beta-actin is shown as a loading control.

(E) Immunohistochemistry for cleaved caspase-3 (×100) or γH2AX (×200, inset ×400) of sections of the periventricular region of the developing brain, from an *Aldh2*$^{+/-}$*Fanca*$^{+/-}$ or an *Aldh2*$^{-/-}$*Fanca*$^{-/-}$ embryo at E12.5. Red arrows denote cells staining positive for cleaved caspase-3 or γH2AX.

embryo transfer experiment allows us to interrogate the developmental fate of *Aldh2*$^{-/-}$*Fanca*$^{-/-}$ embryos, which would otherwise never survive after E12.5 in an *Aldh2*$^{-/-}$ mother or E16.5 in an *Aldh2*$^{+/-}$ mother. Remarkably, we noted that *Aldh2*$^{+/+}$ mothers suppress developmental defects of *Aldh2*$^{-/-}$*Fanca*$^{-/-}$ embryos at E11.5. A total of 25% of *Aldh2*$^{-/-}$*Fanca*$^{-/-}$ embryos from embryo transfer show developmental defects (n = 8), compared to 100% of *Aldh2*$^{-/-}$*Fanca*$^{-/-}$ embryos conceived by *Aldh2*$^{+/-}$ mothers (n = 10) at E11.5 (**p = 0.0015; Figure 5B). Furthermore, when such surrogate pregnancies are allowed to come to term, *Aldh2*$^{-/-}$*Fanca*$^{-/-}$ mice are born for the first time (Figures 5C and S5A). However, these mice are smaller than their littermates and have a high prevalence of developmental defects, including craniofacial abnormalities (Figure 5C, data not shown).

The hallmark of FA is the development of bone marrow failure due to loss of the HSC pool (Ceccaldi et al., 2012). Recent evidence in humans and mice suggests that stem cell defects can be detected in utero (Ceccaldi et al., 2012; Kamimae-Lanning et al., 2013). Our previous work has shown that aldehyde-mediated DNA damage might explain HSC attrition in adult mice, and in this study we show that aldehydes have a profound impact on the development of FA-deficient embryos (Garaycoechea et al., 2012). Now that *Aldh2*$^{-/-}$*Fanca*$^{-/-}$ mice can be rescued through gestation into neonatal life, we wanted to see if these mice had intact hematopoietic stem and progenitor cells (HSPCs) in their bone marrow. The data in Figures 5D and 5E show clearly that there is a significant 15.5-fold depletion of the Lin^{-}cKit^{+}Sca1^{+} (LKS) population (representing the HSPC pool) of these mice, compared to a 2.5-fold depletion in the *Fanca*$^{-/-}$ control.

Furthermore, one single *Aldh2*$^{-/-}$*Fanca*$^{-/-}$ mouse born naturally to an *Aldh2*$^{+/-}$ mother survived the embryonic window and was weaned (a rare occurrence of 1 out of 388 mice). In agreement with the fate of *Aldh2*$^{-/-}$*Fanca*$^{-/-}$ mice born from embryo transfer, this mouse developed bone marrow failure at 7 weeks of age (Figure S5B). Analysis of the bone marrow revealed a severe loss of LKS cells, showing that bone marrow failure was due to depletion of HSPCs. Cumulatively, these data allow us to disassociate the role of maternal and fetal aldehyde catabolism during development. The embryo transfer experiment conclusively demonstrates that wild-type maternal *Aldh2* is necessary and sufficient for the birth of *Aldh2*$^{-/-}$*Fanca*$^{-/-}$ mice. In other words, maternal catabolism is crucial for general development to proceed, but embryonic Aldh2 is essential to prevent development defects and for the overall preservation of blood stem cells.

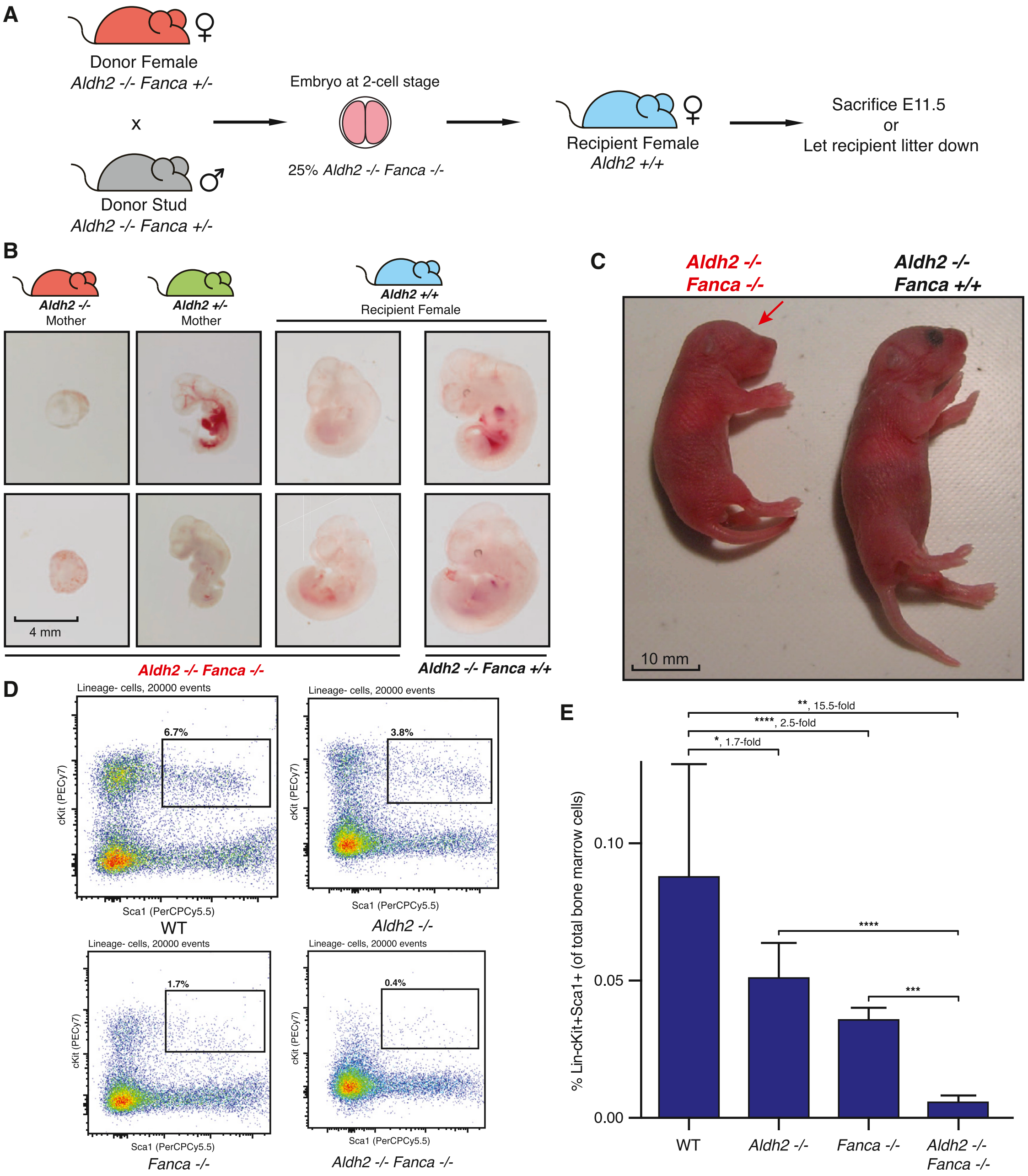

Figure 5. Embryo Transfer of *Aldh2*$^{-/-}$*Fanca*$^{-/-}$ Embryos into *Aldh2*$^{+/+}$ Mothers Rescues Their Development but Not Blood Stem Cells

(A) Schematic overview of the embryo transfer experiment. Embryos at the two-cell stage obtained from intercrossing *Aldh2*$^{-/-}$*Fanca*$^{+/-}$ males (gray) and females (red) were harvested and transferred into the uteri of mothers completely competent at aldehyde catabolism (*Aldh2*$^{+/+}$; blue mouse).

(B) Representative images of *Aldh2*$^{-/-}$*Fanca*$^{-/-}$ embryos at E11.5, generated from either *Aldh2*$^{-/-}$*Fanca*$^{+/-}$ females (left, red mouse), *Aldh2*$^{+/-}$*Fanca*$^{+/-}$ females (middle, green mouse), or *Aldh2*$^{+/+}$ recipient females by embryo transfer (right, blue mouse). *Aldh2*$^{-/-}$*Fanca*$^{+/+}$ embryos at E11.5, generated by embryo transfer into *Aldh2*$^{+/+}$ females, are shown as controls. *Aldh2*$^{+/+}$ mothers suppress the developmental defects of *Aldh2*$^{-/-}$*Fanca*$^{-/-}$ embryos at E11.5.

(C) Photograph of an *Aldh2*$^{-/-}$*Fanca*$^{-/-}$ pup at P1 (1 day postpartum) next to an *Aldh2*$^{-/-}$*Fanca*$^{+/+}$ littermate control. Both pups were born to an *Aldh2*$^{+/+}$ mother, using embryo transfer. Red arrow shows the lack of an eye in the *Aldh2*$^{-/-}$*Fanca*$^{-/-}$ pup.

(D) Representative flow cytometry profiles of bone marrow cells obtained from P1 pups of various genotypes born from embryo transfer (for *Aldh2*$^{-/-}$*Fanca*$^{-/-}$ pups and *Aldh2*$^{-/-}$ control) or through natural crosses (for WT and *Fanca*$^{-/-}$ controls). The cells were stained for lineage markers, c-Kit and Sca-1, and the profiles show 20,000 lineage-negative events. The box denotes the Lin^{-}cKit^{+}Sca1^{+} (LKS) population, which is enriched for hematopoietic stem cells.

(E) Bar chart showing the quantification of hematopoietic stem and progenitor cell (HSPC) populations assessed by flow cytometry in (D). Unpaired t test; *$p < 0.05$, **$p < 0.005$, ***$p < 0.0005$, ***$p < 0.0001$, 5% confidence interval. Error bars represent SD. n = 12 for WT, n = 24 for Aldh2$^{-/-}$, n = 3 for Fanca$^{-/-}$, n = 3 for *Aldh2*$^{-/-}$*Fanca*$^{-/-}$.

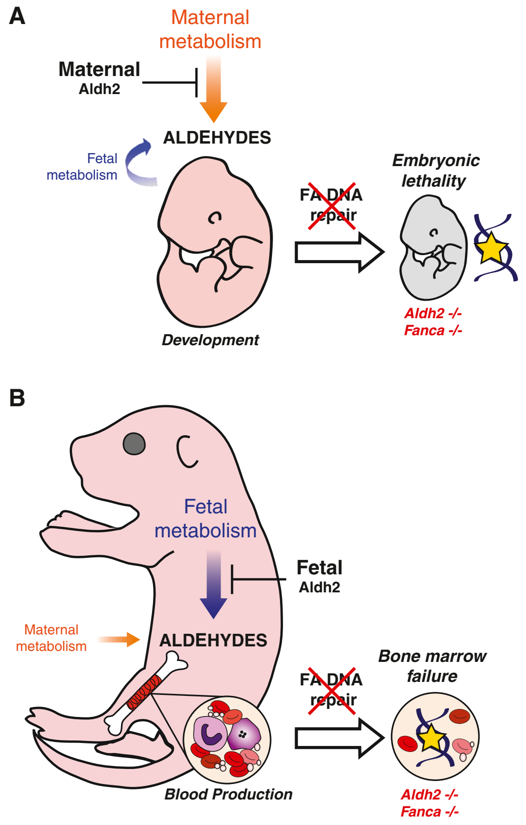

Figure 6. Model for the Role of Aldehyde Catabolism and FA DNA Repair during Development

(A) In early development, maternal metabolism produces aldehydes, which diffuse across the placenta and damage the DNA of the developing embryo. Fetal metabolism may also produce aldehydes, which disrupt development. Maternal and fetal Aldh2 play a key role in detoxifying these aldehydes and protecting the early embryo from developmental defects.

(B) In later development, the embryo is larger and produces a greater aldehyde burden from fetal metabolism; fetal Aldh2 counteracts this in order to prevent developmental defects. In addition, there is an organism intrinsic requirement for Aldh2 and FA DNA repair in the fetus, which together are required to protect bone marrow stem cells from accumulating DNA damage and undergoing attrition.

DISCUSSION

The work presented in this paper defines a basic mechanism for the protection of the growing embryo from genotoxic aldehydes. This mechanism operates at three levels. First, the mother detoxifies aldehydes that she produces from her circulation. Second, the embryo is reliant on the FA DNA repair pathway to respond to and repair DNA damage caused by these aldehydes that have evaded both maternal and fetal removal. Third, embryonic aldehyde catabolism is essential to preserve bone marrow HSPCs in utero (Figure 6).

Aldehyde removal through maternal Aldh2 is the primary protective mechanism to shield the early embryo from these genotoxins. There is increasing evidence to show that early insults to the fetus, including DNA damage, can lead to disease in adult life, a process known as intrauterine programming (Fowden et al., 2005). Human epidemiological studies have revealed that impaired intrauterine growth, due to lack of availability of nutrients, oxygen, or hormones during pregnancy, is linked to the development of diabetes and other metabolic disorders (Fowden et al., 2006). In addition, mice with a hypomorphic mutation in the DNA damage response protein ATR develop a progeroid syndrome, and this phenotype is due to high levels of replicative stress and DNA damage during embryogenesis, but not in adult tissues (Murga et al., 2009). In agreement with previous work on intrauterine programming, our study suggests that in utero exposure to aldehydes, which in certain circumstances can lead to the accumulation of DNA damage in the embryo, may be a mechanism that explains the origin of congenital abnormalities in humans. Our data also point to acetaldehyde-mediated DNA damage in utero being responsible for the developmental defects seen in the fetal alcohol syndrome, which is caused by excessive maternal ethanol consumption during pregnancy. Lastly, given the evidence that childhood leukemias are often initiated in utero, this raises an intriguing possibility that aldehyde-induced DNA damage during gestation contributes to early genetic changes that cause these neoplasmas (Clarkson and Boyse, 1971; Greaves and Wiemels, 2003; van Dijk et al., 1996).

The genetic interaction between the mother and the embryo presented in this paper is highly unusual in the mammal. Genetic screens in flies and zebrafish have uncovered many instances of this class of interaction; however, this is mainly due to a requirement for maternal RNA to express essential proteins very early in development (Anderson and Nüsslein-Volhard, 1984; Nüsslein-Volhard et al., 1987; Pelegri et al., 1999). In mammals, there are just two other clear examples of maternal lethal zygotic effects that affect later stages of embryonic development (Li et al., 2008; Rutschmann et al., 2012). Our work further underscores the importance of considering the maternal genotype (in addition to allele segregation) when assessing embryonic phenotypes. In this case, it seems most likely that the maternal biomass, which in early gestation is considerably larger than the embryo, produces acetaldehyde or another substrate of Aldh2. The widespread expression of Aldh2 would suggest that these aldehydes are probably broken down before they access the embryo. Two other enzymes (Aldh1a1 and Aldh1b1) are also capable of removing the same aldehydes; however, our results indicate that Aldh2 deficiency cannot be fully compensated by them. This may be due to the lower enzymatic efficiency or subcellular localization of these enzymes (Klyosov et al., 1996; Peng and Yin, 2009; Stagos et al., 2010). We were surprised to note that the placenta seems completely devoid of expression of any of the three Aldhs, and our enzymatic assay confirms negligible activity in placenta from wild-type mice. It is possible that an endogenous aldehyde may be required for the normal physiology of this tissue, which would explain the absence of aldehyde dehydrogenase activity.

Maternal embryo transfer allowed us to further confirm the relevance of maternal versus fetal aldehyde catabolism. Our results show that *Aldh2*$^{+/+}$ mothers suppress the embryonic lethality of *Aldh2*$^{-/-}$*Fanca*$^{-/-}$ mice. However, these embryos are developmentally compromised, indicating that fetal Aldh2 is required to prevent developmental abnormalities. This is further emphasized by the severe lack of HSPCs in the bone

marrow of these neonates. It would seem that as the embryo grows, its own metabolism generates aldehydes, which, if not cleared by the fetus, damage HSPCs and lead to their attrition. Alternatively, there may be a cell-intrinsic requirement for Aldh2 and FA DNA repair in the HSPCs themselves in order to protect them against damage and attrition. Nevertheless, these results are in agreement with recent studies that indicate that bone marrow dysfunction in FA begins in utero (Ceccaldi et al., 2012; Kamimae-Lanning et al., 2013).

So far, we have focused on how the DNA of the embryo is shielded from aldehydes; the other key protection mechanism is fetal DNA repair. The FA pathway acts in a DNA crosslink repair process that requires the Rev1 protein (Niedzwiedz et al., 2004; Ross et al., 2005). However, neither maternal nor fetal Aldh2 appear essential for the development of *Aldh2*$^{-/-}$*Rev1*$^{-/-}$ embryos. In fact, these embryos can be conceived by *Aldh2*$^{-/-}$ mothers and appear indistinguishable from *Rev1*$^{-/-}$ embryos. Furthermore, *Aldh2*$^{-/-}$*Rev1*$^{-/-}$ neonates do not have a reduced HSPC pool compared to *Rev1*$^{-/-}$ pups (Figure S6B). Consistent with these findings, *Rev1*$^{-/-}$ hematopoietic cells are no more sensitive to acetaldehyde than wild-type cells (Figure S6A).

The phenotype of *Aldh2*$^{-/-}$*Rev1*$^{-/-}$ diverges significantly from the phenotype of *Aldh2*$^{-/-}$*Fanca*$^{-/-}$ mice. This is a surprising result, as Rev1 and the FA proteins act in a common pathway to maintain resistance to chemotherapy-induced crosslinking agents. These data suggest that the genetic requirements to repair DNA damage caused by an Aldh2 substrate differ significantly from the requirements to repair canonical chemotherapy-induced crosslinks. Future work will be needed to define precisely the chemical nature of DNA damage created by aldehydes, as this may not be a simple DNA interstrand crosslink.

As already mentioned, FA is characterized by developmental defects and bone marrow failure (Alter, 2003; Kutler et al., 2003). In most instances, the mother and embryo are likely to be aldehyde catabolism proficient. However, it is possible that in certain instances aldehyde levels in the embryo might spike—most obviously when a mother consumes alcohol in the first trimester of pregnancy. Such situations would require intact FA repair in order to reverse aldehyde-induced DNA damage. Approximately 540 million individuals worldwide (mainly across Southeast Asia and Japan) are genetically deficient in *ALDH2* (*ALDH2*2* allele) (Brooks et al., 2009). The molecular defect is due to single amino acid substitution (E487K) that causes a dominant-negative mutant form of ALDH2, resulting in heterozygotes having only 5%–15% of wild-type ALDH2 activity (Brooks et al., 2009; Harada et al., 1981; Yoshida et al., 1981, 1984). This provides a unique opportunity to study the impact of ALDH2 deficiency and FA in humans. A recent study looked at the clinical progression of FA in children of Japanese origin that also segregate the *ALDH2* mutant allele, *ALDH2*2* (Hira et al., 2013). This study clearly shows that ALDH2 deficiency leads to a much more rapid progression of bone marrow failure. However, this study also shows that ALDH2 deficiency does not lead to more developmental defects. This is a surprise when put into the context of our work in mice, but it is worth noting two factors: first, the genotype of the mothers is not known, and second, two of the three patients that were homozygous for *ALDH2*2* carried extensive developmental malformations. These children could only be born to *ALDH2*2* heterozygous or homozygous mothers. Nevertheless, it will be crucial to combine the phenotypic analysis of Southeast Asian FA patients with their maternal *ALDH2* status.

Finally, the many humans carrying the *ALDH2*2* allele are known to be sensitive to alcohol, and alcohol consumption in this group enhances their risk of developing head and neck cancer (Brooks et al., 2009). However, it is not known if this common allele also impacts on fetal wellbeing and the prevalence of birth defects, particularly with alcohol consumption during pregnancy. Future studies should address this question, which is of clear importance for public health and preventative medicine in this populous part of the world.

EXPERIMENTAL PROCEDURES

Please refer to the Supplemental Experimental Procedures for detailed methodology on flow cytometry, survival assays, and comprehensive aldehyde dehydrogenase activity assay conditions.

Mice

Aldh2$^{-/-}$ and *Fanca*$^{-/-}$ mice on a C57BL6/Jo1a background were described previously (Garaycoechea et al., 2012; Langevin et al., 2011). *Aldh2*-deficient mice were generated from embryonic stem cells (ESCs) obtained from EUCOMM (*Aldh2*$^{tm1a(EUCOMM)Wtsi}$; Mouse Genome Informatics [MGI] code: 4431566). *Fanca*-deficient mice were generated from ESCs, obtained from EUCOMM (*Fanca*$^{tm1a(EUCOMM)Wtsi}$; MGI code: 4434431). *Rev1*-deficient mice were a gift from N. de Wind (*Rev1*^{tm1Ndew}; MGI code: 3701945) and described previously (Jansen et al., 2006). Once imported, these mice were maintained in the C57BL6/Jo1a background. All animals were maintained in specific pathogen-free conditions. All animal experiments undertaken in this study were done so with the approval of the UK Home Office.

Histology

Histological analysis was performed on whole embryos fixed in neutral buffered formalin for 24 hr. The samples were then paraffin embedded, and 4 μm sections were cut before being stained with hematoxylin and eosin. For immunohistochemistry, samples were cut and stained as described previously (Langevin et al., 2011), using rabbit anti-phospho-H2AX (Cell Signaling, 2577, 1:50) and rabbit anti-cleaved caspase-3 (Cell Signaling, Asp175 9661L, 1:100).

Flow Cytometry

Flow cytometry was performed on bone marrow, spleen, and thymic cells that were isolated from mutant mice and appropriate controls. Bone marrow cells were obtained either by flushing from the femora and tibiae (adult bones) or by crushing the femora, tibiae, and humeri (P1 pups) and passing through a 40 μm filter. The cells were stained as described previously (Garaycoechea et al., 2012), and samples were run on a LSRII flow cytometer (BD Pharmingen), and the data were analyzed with FlowJo 10.0.7 (Tree Star).

Timed Matings

Timed matings of *Aldh2*$^{-/-}$*Fanca*$^{+/-}$, *Aldh2*$^{+/-}$*Fanca*$^{+/-}$, and *Fanca*$^{+/-}$ females were set up with corresponding males. Females were checked for the presence of a vaginal plug the following morning, which was considered day E0.5. Females were then killed at E10.5–E12.5 or E16.5, and embryos or resorbed sacs were removed for genotyping and analysis. For the acute ethanol during pregnancy experiment, plugged females were injected with 4.5 g/kg of a 28% ethanol solution at E7.5 in two separate intraperitoneal injections of 2.25 g/kg, 4 hr apart. At E13.5, pregnant females were killed and uteri taken for dissection of embryos.

Western Blotting

Western blot extracts were prepared from whole individual embryos (with the fetal livers removed) or various tissues by disruption in RIPA buffer (0.1% SDS, 50 mM Tris-HCl [pH 7.4], 150 mM NaCl, 0.5% Na-deoxycholate, 1% NP-40,

1 mM EDTA, serine/threonine phosphatase inhibitor cocktail (PhosphoStop, Sigma-Aldrich), and protease inhibitor cocktail (Roche) using a QIAGEN TissueLyser II. γH2AX monoclonal antibody (Millipore, JBW301) was used at 1:1,000. Beta-actin polyclonal antibody (Abcam, ab8227) was used at 1:2,000. Aldh2 (Proteintech, 15310-1-AP), Aldh1b1 (Proteintech, 15560-1-AP), and Aldh1a1 (Proteintech, 15910-1-AP) polyclonal antibodies were used at 1:2,000.

Embryo Transfer

Aldh2$^{-/-}$*Fanca*$^{+/-}$ female donor mice (4 weeks old) were injected with five international units of pregnant mare's serum (PMS) and human chorionic gonadotrophin (hCG) intraperitoneally on day 1. On day 3, they were placed overnight with a proven *Aldh2*$^{-/-}$*Fanca*$^{+/-}$ stud, and on day 4 potentially pregnant donors were identified by the presence of a vaginal plug. These females were sacrificed, and two-cell-stage embryos were flushed from their uteri. These were washed in M2 media and maintained in M16 media under mineral oil in a CO_2 incubator until being transferred into recipients. On day 3, recipient wild-type C57BL6/Jo1a females, weighing 20–30 g, were mated overnight with vasectomized (CD1) males to produce pseudopregnant recipients. These were identified on day 4 as being positive for a vaginal plug, and up to 16 two-cell embryos were then implanted into their uteri unilaterally in a surgical manner. The recipient females were then sacrificed 11 days after embryo transfer for dissection of embryos or allowed to litter down for analysis of P1 pups.

Aldehyde Dehydrogenase Activity Assay

Mouse livers, whole E13.5 embryos, or placentas were homogenized in order to prepare a crude mitochondrial extract. This extract was subsequently used to perform Aldh enzymatic activity assays. Reactions were set up in a cuvette containing the crude mitochondrial extract, NaPPi buffer (pH 9.0), NAD^+, and the substrate acetaldehyde. After substrate addition, the absorbance at 340 nm was recorded using a Cary 5000 UV-Vis-NIR spectrophotometer at room temperature (RT) for 350 s. To calculate the [NADH] production in mol/min/mg total protein, we used the equation absorbance = $\varepsilon \times c \times L$, where $\varepsilon = 6220\ M^{-1}$, L = path length (1 cm), and c = [NADH] in mol. The assay was adapted from a protocol by D. Mochly-Rosen, Stanford University.

SUPPLEMENTAL INFORMATION

Supplemental Information includes Supplemental Experimental Procedures and six figures and can be found with this article online at http://dx.doi.org/10.1016/j.molcel.2014.07.010.

AUTHOR CONTRIBUTIONS

N.O., F.L., G.P.C., and K.J.P. designed the study and wrote the paper. N.O. performed the majority of experiments presented. F.L. helped characterize developmental failure throughout the study and analyzed Rev1 mice. N.d.W. provided *Rev1*-deficient mice. G.K. performed embryo transfer experiments.

ACKNOWLEDGMENTS

The authors wish to thank Annabelle Curry and all E29 staff for their help with animal husbandry and animal experiments. We thank Neil Grant for his invaluable help with photography of embryos and Mark Arends for his help in analyzing histological samples. The authors would also like to thank Charlotte Sutherell for her help in conducting initial aldehyde dehydrogenase activity assays. The authors would like to thank all members of the Patel laboratory for critical reading of the manuscript. G.P.C. is funded by CRUK, and F.L. is funded by March of Dimes and Children with Cancer UK.

Received: April 24, 2014
Revised: June 27, 2014
Accepted: July 15, 2014
Published: August 21, 2014

REFERENCES

Alter, B.P. (2003). Cancer in Fanconi anemia, 1927-2001. Cancer *97*, 425–440.

Alter, B.P., and Rosenberg, P.S. (2013). VACTERL-H Association and Fanconi Anemia. Mol Syndromol *4*, 87–93.

Anderson, K.V., and Nüsslein-Volhard, C. (1984). Information for the dorsal—ventral pattern of the Drosophila embryo is stored as maternal mRNA. Nature *311*, 223–227.

Arnon, J., Meirow, D., Lewis-Roness, H., and Ornoy, A. (2001). Genetic and teratogenic effects of cancer treatments on gametes and embryos. Hum. Reprod. Update *7*, 394–403.

Brooks, P.J., Enoch, M.A., Goldman, D., Li, T.K., and Yokoyama, A. (2009). The alcohol flushing response: an unrecognized risk factor for esophageal cancer from alcohol consumption. PLoS Med. *6*, e50.

Ceccaldi, R., Parmar, K., Mouly, E., Delord, M., Kim, J.M., Regairaz, M., Pla, M., Vasquez, N., Zhang, Q.S., Pondarre, C., et al. (2012). Bone marrow failure in Fanconi anemia is triggered by an exacerbated p53/p21 DNA damage response that impairs hematopoietic stem and progenitor cells. Cell Stem Cell *11*, 36–49.

Clarkson, B.D., and Boyse, E.A. (1971). Possible explanation of the high concoddance for acute leukaemia in monozygotic twins. Lancet *1*, 699–701.

Fowden, A.L., Giussani, D.A., and Forhead, A.J. (2005). Endocrine and metabolic programming during intrauterine development. Early Hum. Dev. *81*, 723–734.

Fowden, A.L., Giussani, D.A., and Forhead, A.J. (2006). Intrauterine programming of physiological systems: causes and consequences. Physiology (Bethesda) *21*, 29–37.

Garaycoechea, J.I., and Patel, K.J. (2014). Why does the bone marrow fail in Fanconi anemia? Blood *123*, 26–34.

Garaycoechea, J.I., Crossan, G.P., Langevin, F., Daly, M., Arends, M.J., and Patel, K.J. (2012). Genotoxic consequences of endogenous aldehydes on mouse haematopoietic stem cell function. Nature *489*, 571–575.

Greaves, M.F., and Wiemels, J. (2003). Origins of chromosome translocations in childhood leukaemia. Nat. Rev. Cancer *3*, 639–649.

Hall, E.J. (1991). Scientific view of low-level radiation risks. Radiographics *11*, 509–518.

Harada, S., Agarwal, D.P., and Goedde, H.W. (1981). Aldehyde dehydrogenase deficiency as cause of facial flushing reaction to alcohol in Japanese. Lancet *2*, 982.

Hira, A., Yabe, H., Yoshida, K., Okuno, Y., Shiraishi, Y., Chiba, K., Tanaka, H., Miyano, S., Nakamura, J., Kojima, S., et al. (2013). Variant ALDH2 is associated with accelerated progression of bone marrow failure in Japanese Fanconi anemia patients. Blood *122*, 3206–3209.

Hodskinson, M.R., Silhan, J., Crossan, G.P., Garaycoechea, J.I., Mukherjee, S., Johnson, C.M., Schärer, O.D., and Patel, K.J. (2014). Mouse SLX4 is a tumor suppressor that stimulates the activity of the nuclease XPF-ERCC1 in DNA crosslink repair. Mol. Cell *54*, 472–484.

Jansen, J.G., Langerak, P., Tsaalbi-Shtylik, A., van den Berk, P., Jacobs, H., and de Wind, N. (2006). Strand-biased defect in C/G transversions in hypermutating immunoglobulin genes in Rev1-deficient mice. J. Exp. Med. *203*, 319–323.

Kamimae-Lanning, A.N., Goloviznina, N.A., and Kurre, P. (2013). Fetal origins of hematopoietic failure in a murine model of Fanconi anemia. Blood *121*, 2008–2012.

Klein Douwel, D., Boonen, R.A., Long, D.T., Szypowska, A.A., Räschle, M., Walter, J.C., and Knipscheer, P. (2014). XPF-ERCC1 acts in Unhooking DNA interstrand crosslinks in cooperation with FANCD2 and FANCP/SLX4. Mol. Cell *54*, 460–471.

Klyosov, A.A., Rashkovetsky, L.G., Tahir, M.K., and Keung, W.M. (1996). Possible role of liver cytosolic and mitochondrial aldehyde dehydrogenases in acetaldehyde metabolism. Biochemistry *35*, 4445–4456.

Knipscheer, P., Räschle, M., Smogorzewska, A., Enoiu, M., Ho, T.V., Schärer, O.D., Elledge, S.J., and Walter, J.C. (2009). The Fanconi anemia pathway promotes replication-dependent DNA interstrand cross-link repair. Science *326*, 1698–1701.

Kutler, D.I., Singh, B., Satagopan, J., Batish, S.D., Berwick, M., Giampietro, P.F., Hanenberg, H., and Auerbach, A.D. (2003). A 20-year perspective on the International Fanconi Anemia Registry (IFAR). Blood *101*, 1249–1256.

Langevin, F., Crossan, G.P., Rosado, I.V., Arends, M.J., and Patel, K.J. (2011). Fancd2 counteracts the toxic effects of naturally produced aldehydes in mice. Nature *475*, 53–58.

Li, X., Ito, M., Zhou, F., Youngson, N., Zuo, X., Leder, P., and Ferguson-Smith, A.C. (2008). A maternal-zygotic effect gene, Zfp57, maintains both maternal and paternal imprints. Dev. Cell *15*, 547–557.

Murga, M., Bunting, S., Montaña, M.F., Soria, R., Mulero, F., Cañamero, M., Lee, Y., McKinnon, P.J., Nussenzweig, A., and Fernandez-Capetillo, O. (2009). A mouse model of ATR-Seckel shows embryonic replicative stress and accelerated aging. Nat. Genet. *41*, 891–898.

Neveling, K., Endt, D., Hoehn, H., and Schindler, D. (2009). Genotype-phenotype correlations in Fanconi anemia. Mutat. Res. *668*, 73–91.

Niedzwiedz, W., Mosedale, G., Johnson, M., Ong, C.Y., Pace, P., and Patel, K.J. (2004). The Fanconi anaemia gene FANCC promotes homologous recombination and error-prone DNA repair. Mol. Cell *15*, 607–620.

Nüsslein-Volhard, C., Frohnhöfer, H.G., and Lehmann, R. (1987). Determination of anteroposterior polarity in Drosophila. Science *238*, 1675–1681.

O'Shea, K.S., and Kaufman, M.H. (1979). The teratogenic effect of acetaldehyde: implications for the study of the fetal alcohol syndrome. J. Anat. *128*, 65–76.

Pelegri, F., Knaut, H., Maischein, H.M., Schulte-Merker, S., and Nüsslein-Volhard, C. (1999). A mutation in the zebrafish maternal-effect gene nebel affects furrow formation and vasa RNA localization. Curr. Biol. *9*, 1431–1440.

Peng, G.S., and Yin, S.J. (2009). Effect of the allelic variants of aldehyde dehydrogenase ALDH2*2 and alcohol dehydrogenase ADH1B*2 on blood acetaldehyde concentrations. Hum. Genomics *3*, 121–127.

Räschle, M., Knipscheer, P., Enoiu, M., Angelov, T., Sun, J., Griffith, J.D., Ellenberger, T.E., Schärer, O.D., and Walter, J.C. (2008). Mechanism of replication-coupled DNA interstrand crosslink repair. Cell *134*, 969–980.

Rogakou, E.P., Pilch, D.R., Orr, A.H., Ivanova, V.S., and Bonner, W.M. (1998). DNA double-stranded breaks induce histone H2AX phosphorylation on serine 139. J. Biol. Chem. *273*, 5858–5868.

Rosado, I.V., Langevin, F., Crossan, G.P., Takata, M., and Patel, K.J. (2011). Formaldehyde catabolism is essential in cells deficient for the Fanconi anemia DNA-repair pathway. Nat. Struct. Mol. Biol. *18*, 1432–1434.

Ross, A.L., Simpson, L.J., and Sale, J.E. (2005). Vertebrate DNA damage tolerance requires the C-terminus but not BRCT or transferase domains of REV1. Nucleic Acids Res. *33*, 1280–1289.

Rutschmann, S., Crozat, K., Li, X., Du, X., Hanselman, J.C., Shigeoka, A.A., Brandl, K., Popkin, D.L., McKay, D.B., Xia, Y., et al. (2012). Hypopigmentation and maternal-zygotic embryonic lethality caused by a hypomorphic mbtps1 mutation in mice. G3 (Bethesda) *2*, 499–504.

Stagos, D., Chen, Y., Brocker, C., Donald, E., Jackson, B.C., Orlicky, D.J., Thompson, D.C., and Vasiliou, V. (2010). Aldehyde dehydrogenase 1B1: molecular cloning and characterization of a novel mitochondrial acetaldehyde-metabolizing enzyme. Drug Metab. Dispos. *38*, 1679–1687.

Streffer, C., Shore, R., Konermann, G., Meadows, A., Uma Devi, P., Preston Withers, J., Holm, L.E., Stather, J., and Mabuchi, K.; H R (2003). Biological effects after prenatal irradiation (embryo and fetus). A report of the International Commission on Radiological Protection. Ann. ICRP *33*, 5–206.

Toledo, T.M., Harper, R.C., and Moser, R.H. (1971). Fetal effects during cyclophosphamide and irradiation therapy. Ann. Intern. Med. *74*, 87–91.

van Dijk, B.A., Boomsma, D.I., and de Man, A.J. (1996). Blood group chimerism in human multiple births is not rare. Am. J. Med. Genet. *61*, 264–268.

Webster, W.S., Walsh, D.A., McEwen, S.E., and Lipson, A.H. (1983). Some teratogenic properties of ethanol and acetaldehyde in C57BL/6J mice: implications for the study of the fetal alcohol syndrome. Teratology *27*, 231–243.

Yoshida, A., Impraim, C.C., and Huang, I.Y. (1981). Enzymatic and structural differences between usual and atypical human liver alcohol dehydrogenases. J. Biol. Chem. *256*, 12430–12436.

Yoshida, A., Huang, I.Y., and Ikawa, M. (1984). Molecular abnormality of an inactive aldehyde dehydrogenase variant commonly found in Orientals. Proc. Natl. Acad. Sci. USA *81*, 258–261.

Yoshida, A., Hsu, L.C., and Davé, V. (1992). Retinal oxidation activity and biological role of human cytosolic aldehyde dehydrogenase. Enzyme *46*, 239–244.

Molecular Cell, Volume *55*

Supplemental Information

Maternal Aldehyde Elimination during Pregnancy Preserves the Fetal Genome

Nina Oberbeck, Frédéric Langevin, Gareth King, Niels de Wind, Gerry P. Crossan, and Ketan J. Patel

Figure S1

A

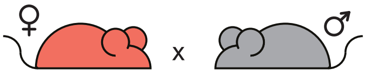

Aldh2 -/- *Fanca+/-* x *Aldh2 +/- Fanca +/-*

Genotype of off-spring **3 weeks old**	n = 186		
	F_{exp}	F_{obs}	p value
Aldh2 +/- Fanca +/+	*12.5 %*	15.1% n = 28	0.8865
Aldh2 -/- Fanca +/+	*12.5 %*	18.8% n = 35	0.5852
Aldh2 +/- Fanca +/-	*25 %*	25.8% n = 48	0.8146
Aldh2 -/- Fanca +/-	*25 %*	39.2% n = 73	* 0.0217
Aldh2 +/- Fanca -/-	***12.5 %***	**1.1% n = 2**	*** 0.0114**
Aldh2 -/- Fanca -/-	***12.5 %***	**0% n = 0**	***** 0.0004**

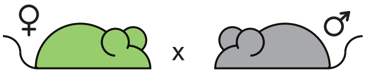

Aldh2 +/- *Fanca +/-* x *Aldh2 -/- Fanca +/-*

Genotype of off-spring **3 weeks old**	n = 388		
	F_{exp}	F_{obs}	p value
Aldh2 +/- Fanca +/+	*12.5 %*	15.5% n = 60	0.9215
Aldh2 -/- Fanca +/+	*12.5 %*	18.6% n = 72	0.3927
Aldh2 +/- Fanca +/-	*25 %*	31.2% n = 121	0.3056
Aldh2 -/- Fanca +/-	*25 %*	29.4% n = 114	0.6332
Aldh2 +/- Fanca -/-	***12.5 %***	**5.2% n = 20**	**0.8763**
Aldh2 -/- Fanca -/-	***12.5 %***	**0.003% n = 1**	****** < 0.0001**

B

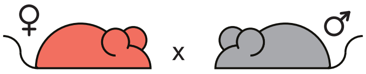

Aldh2 -/- *Fanca+/-* x *Aldh2 +/- Fanca +/-*

Genotype of embryos **E16.5**	n = 59		
	F_{exp}	F_{obs}	p value
Aldh2 +/- Fanca +/+	*12.5 %*	10.2% n = 6	1.0000
Aldh2 -/- Fanca +/+	*12.5 %*	22% n = 13	0.2193
Aldh2 +/- Fanca +/-	*25 %*	25.4% n = 15	1.0000
Aldh2 -/- Fanca +/-	*25 %*	37.3% n = 22	0.2336
Aldh2 +/- Fanca -/-	***12.5 %***	**5.1% n = 3**	**0.3220**
Aldh2 -/- Fanca -/-	***12.5 %***	**0% n = 0**	*** 0.0129**

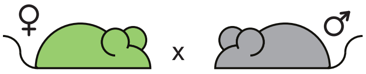

Aldh2 +/- *Fanca +/-* x *Aldh2 -/- Fanca +/-*

Genotype of embryos **E16.5**	n = 64		
	F_{exp}	F_{obs}	p value
Aldh2 +/- Fanca +/+	*12.5 %*	25% n = 16	0.1117
Aldh2 -/- Fanca +/+	*12.5 %*	7.8% n = 5	0.5600
Aldh2 +/- Fanca +/-	*25 %*	20.3% n = 13	0.6733
Aldh2 -/- Fanca +/-	*25 %*	23.4% n = 15	1.0000
Aldh2 +/- Fanca -/-	***12.5 %***	**15.6% n = 10**	**0.8000**
Aldh2 -/- Fanca -/-	***12.5 %***	**7.8% n = 5**	**0.5600**

C

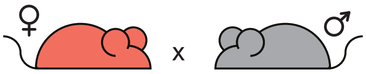

Aldh2 -/- *Fanca+/-* x *Aldh2 +/- Fanca +/-*

Genotype of embryos **E10.5-E12.5**	n = 83		
	F_{exp}	F_{obs}	p value
Aldh2 +/- Fanca +/+	*12.5 %*	19.3% n = 16	0.2855
Aldh2 -/- Fanca +/+	*12.5 %*	13.3% n = 11	1.0000
Aldh2 +/- Fanca +/-	*25 %*	31.3% n = 26	0.4910
Aldh2 -/- Fanca +/-	*25 %*	19.3% n = 16	0.4560
Aldh2 +/- Fanca -/-	***12.5 %***	**15.7% n = 13**	**0.6540**
Aldh2 -/- Fanca -/-	***12.5 %***	**1.2% n = 1**	**** 0.0093**

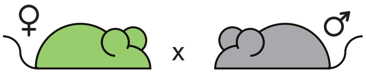

Aldh2 +/- *Fanca +/-* x *Aldh2 -/- Fanca +/-*

Genotype of embryos **E10.5-E12.5**	n = 144		
	F_{exp}	F_{obs}	p value
Aldh2 +/- Fanca +/+	*12.5 %*	10.4% n = 15	0.7119
Aldh2 -/- Fanca +/+	*12.5 %*	10.4% n = 15	0.7119
Aldh2 +/- Fanca +/-	*25 %*	35.4% n = 51	0.0721
Aldh2 -/- Fanca +/-	*25 %*	24.3% n = 35	1.000
Aldh2 +/- Fanca -/-	***12.5 %***	**12.5% n = 18**	**1.000**
Aldh2 -/- Fanca -/-	***12.5 %***	**6.9% n = 10**	**0.1629**

Figure S2

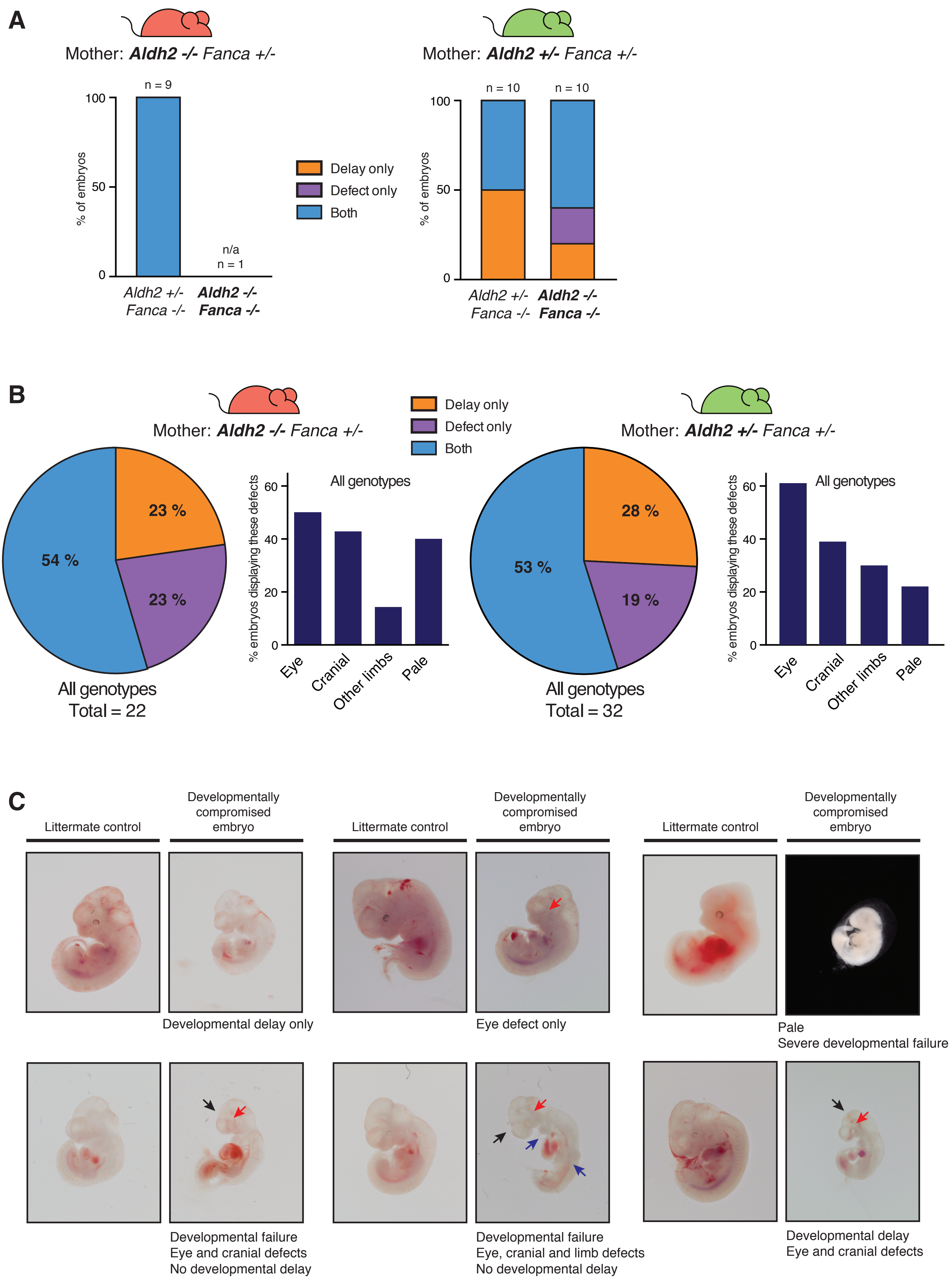
A
Mother: *Aldh2 -/- Fanca +/-*
n = 9
100
50
0
% of embryos
n/a
n = 1
Aldh2 +/- Fanca -/-
Aldh2 -/- Fanca -/-
Delay only
Defect only
Both
Mother: *Aldh2 +/- Fanca +/-*
n = 10
n = 10
100
50
0
% of embryos
Aldh2 +/- Fanca -/-
Aldh2 -/- Fanca -/-
B
Mother: *Aldh2 -/- Fanca +/-*
Delay only
Defect only
Both
Mother: *Aldh2 +/- Fanca +/-*
23 %
23 %
54 %
All genotypes
Total = 22
% embryos displaying these defects
60
40
20
0
All genotypes
Eye
Cranial
Other limbs
Pale
28 %
19 %
53 %
All genotypes
Total = 32
% embryos displaying these defects
60
40
20
0
All genotypes
Eye
Cranial
Other limbs
Pale
C
Littermate control
Developmentally compromised embryo
Littermate control
Developmentally compromised embryo
Littermate control
Developmentally compromised embryo
Developmental delay only
Eye defect only
Pale
Severe developmental failure
Developmental failure
Eye and cranial defects
No developmental delay
Developmental failure
Eye, cranial and limb defects
No developmental delay
Developmental delay
Eye and cranial defects

Figure S3

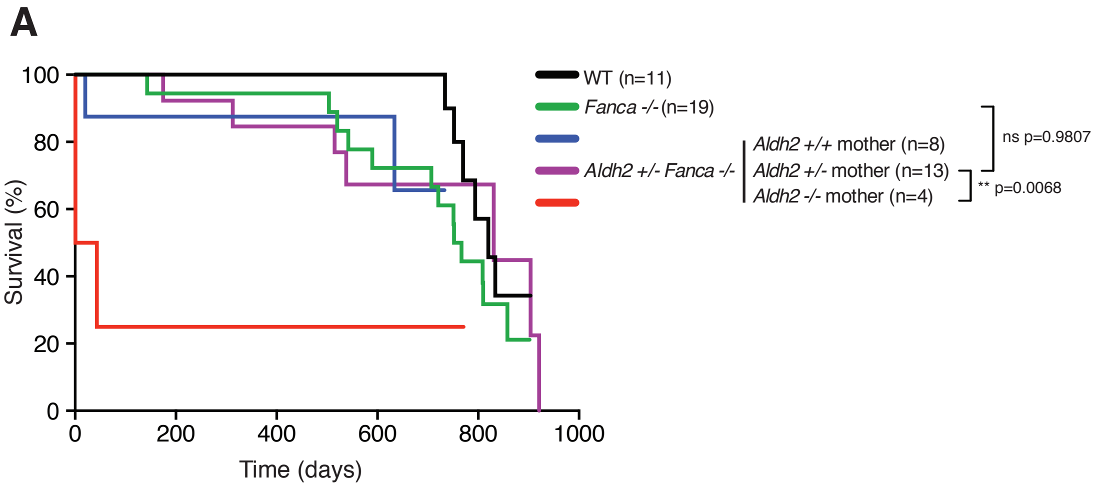
A
Survival (%)
100
80
60
40
20
0
0
200
400
600
800
1000
Time (days)
WT (n=11)
Fanca -/- (n=19)
Aldh2 +/- Fanca -/-
Aldh2 +/+ mother (n=8)
Aldh2 +/- mother (n=13)
Aldh2 -/- mother (n=4)
ns p=0.9807
** p=0.0068

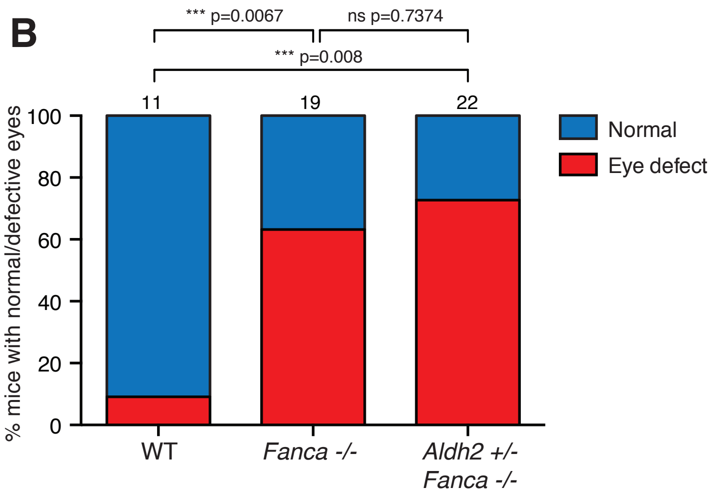
B
*** p=0.0067
ns p=0.7374
*** p=0.008
11
19
22
% mice with normal/defective eyes
100
80
60
40
20
0
WT
Fanca -/-
Aldh2 +/-
Fanca -/-
Normal
Eye defect

Figure S4

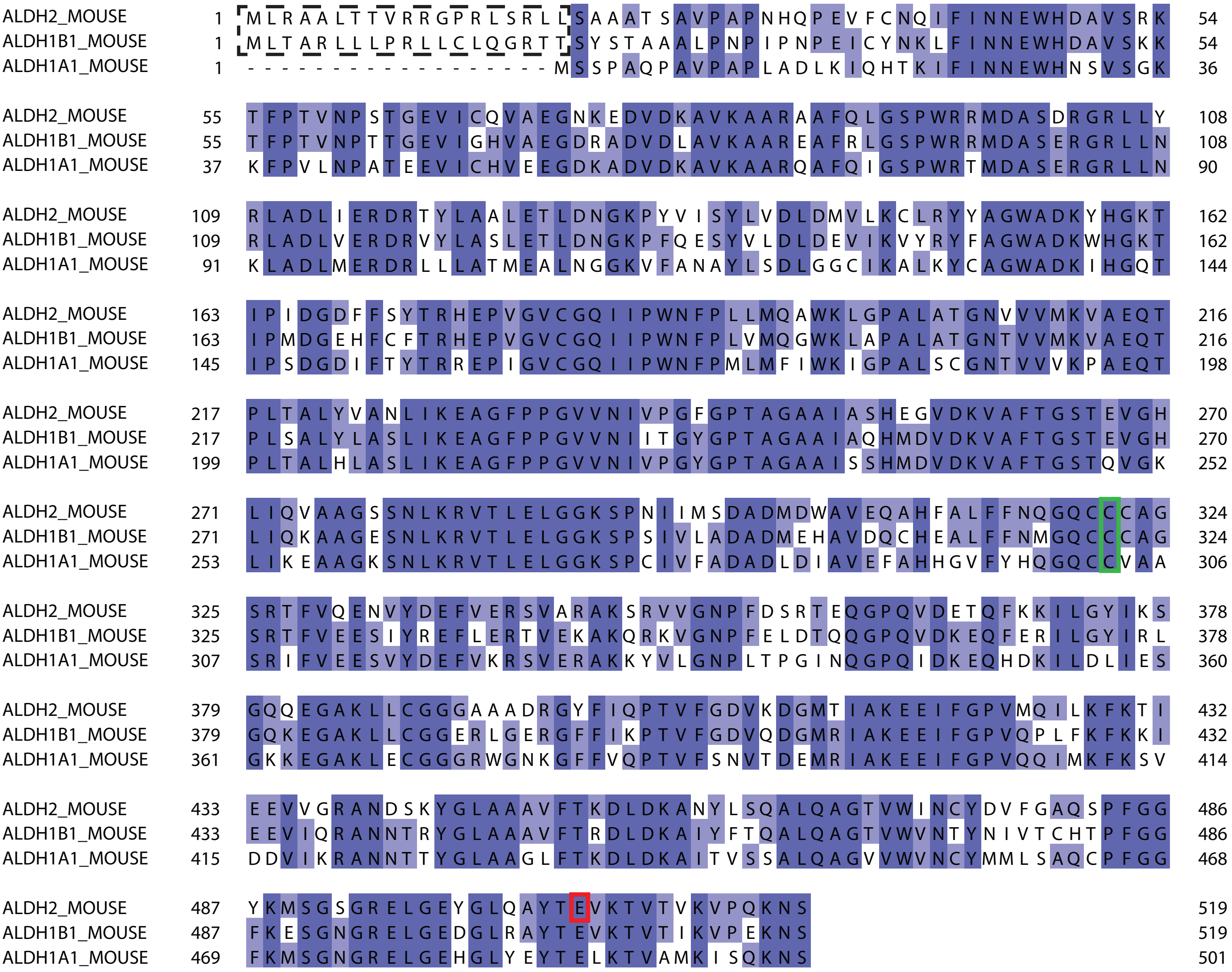

Dotted line denotes mitochondrial targeting sequence

Green box denotes catalytic cysteine

Red box denotes amino acid mutated in the common human *ALDH2* polymorphism (E487K, *Aldh2*2*) responsible for the 'Asian Flush'

Figure S5

A

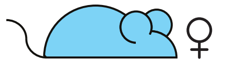

Recipient Female
Aldh2 +/+

P1 pups born from embryo transfer

	Aldh2 -/-			
	Fanca +/+	*Fanca +/-*	*Fanca -/-*	
F_{exp}	25 %	50 %	25 %	
F_{obs}	28.6 %<br>n = 12	59.5 %<br>n = 25	11.9 %<br>n = 5	p = 0.24<br>ns

B

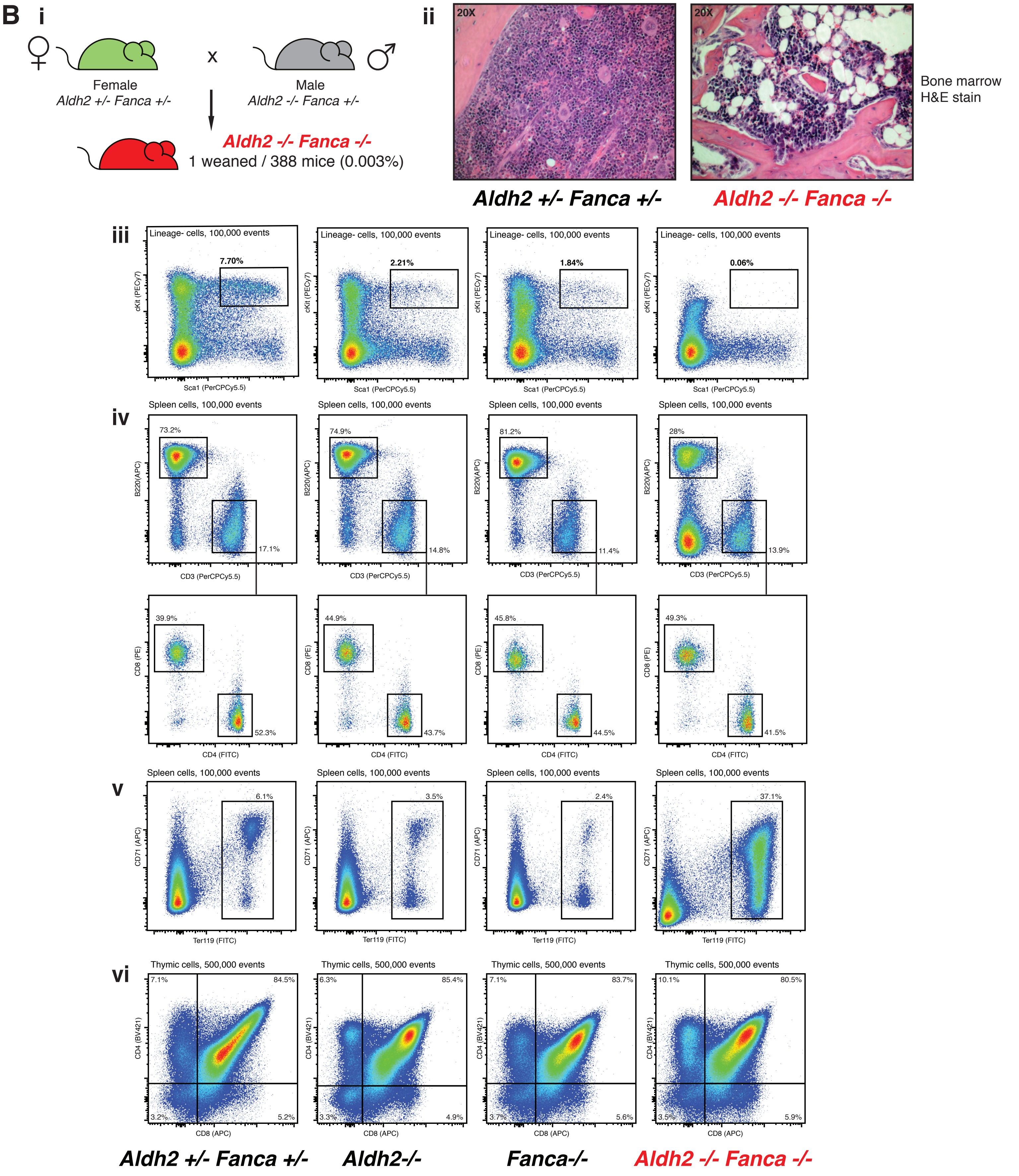

Figure S6

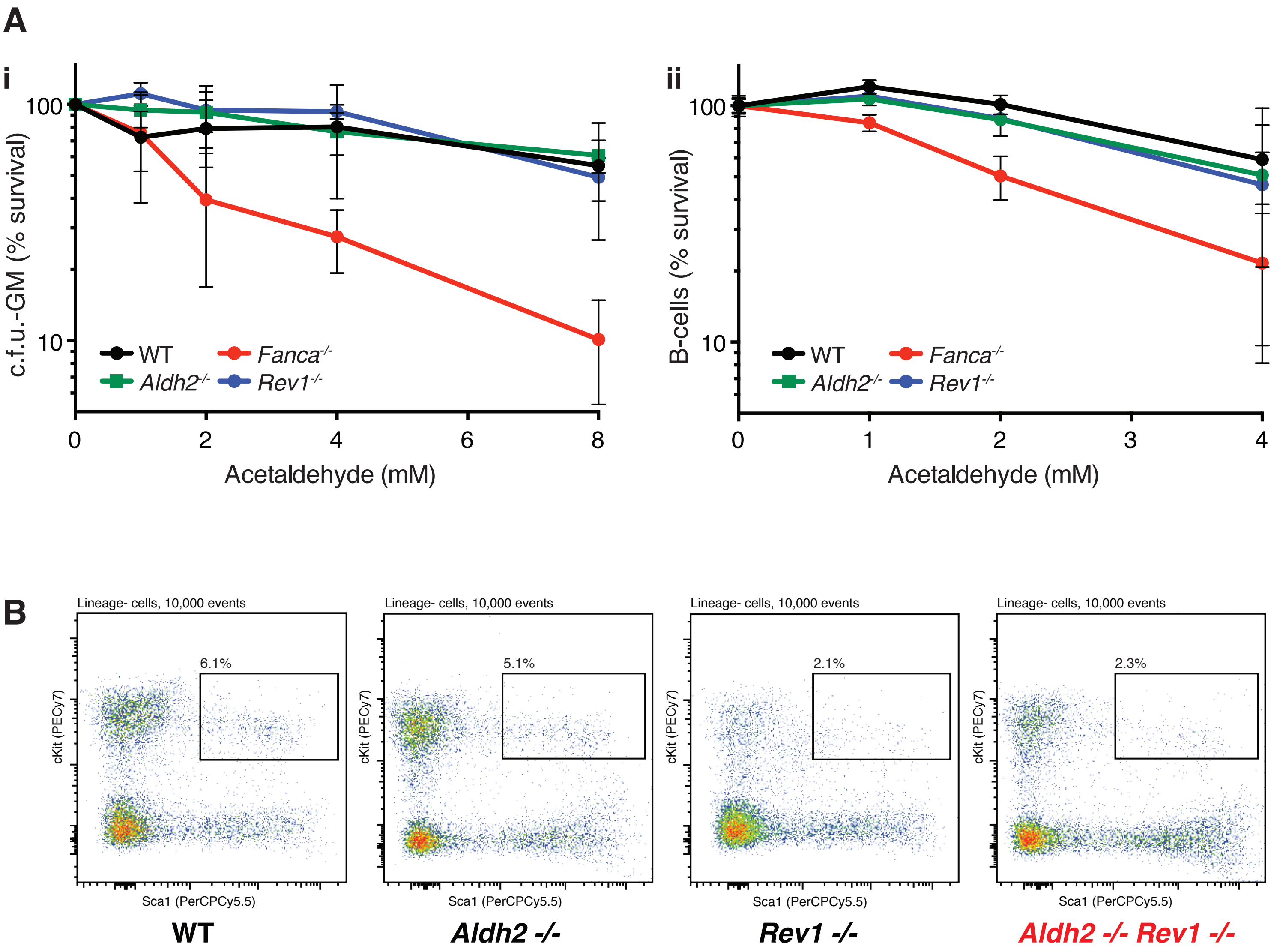
A
i
c.f.u.-GM (% survival)
100
10
WT
Fanca-/-
Aldh2-/-
Rev1-/-
0
2
4
6
8
Acetaldehyde (mM)
ii
B-cells (% survival)
100
10
WT
Fanca-/-
Aldh2-/-
Rev1-/-
0
1
2
3
4
Acetaldehyde (mM)
B
Lineage- cells, 10,000 events
6.1%
cKit (PECy7)
Sca1 (PerCPCy5.5)
WT
Lineage- cells, 10,000 events
5.1%
cKit (PECy7)
Sca1 (PerCPCy5.5)
Aldh2 -/-
Lineage- cells, 10,000 events
2.1%
cKit (PECy7)
Sca1 (PerCPCy5.5)
Rev1 -/-
Lineage- cells, 10,000 events
2.3%
cKit (PECy7)
Sca1 (PerCPCy5.5)
Aldh2 -/- Rev1 -/-

Supplemental Figure Legends

Figure S1, Related to Figure 1: *Aldh2$^{-/-}$Fanca$^{-/-}$* mice are embryonic lethal in the pure C57BL6/Jo1a genetic background

Observed and expected frequencies of all possible genotypes of offspring at **(A)** 3 weeks of age, **(B)** E16.5 and **(C)** E10.5-E12.5, obtained from the intercrosses displayed. The genotypes of interest: *Aldh2$^{+/-}$Fanca$^{+/-}$* and *Aldh2$^{-/-}$Fanca$^{-/-}$* are shaded in grey. Fisher's exact test, 5% confidence interval. For the data involving embryos, genotyped resorptions are not included in the data.

Figure S2, Related to Figure 2A and 2B: Description of the spontaneous developmental defects seen in E10.5-E12.5 embryos

(A) Bar graph displaying the proportion of developmentally compromised E10.5-E12.5 *Aldh2$^{+/-}$Fanca$^{-/-}$* and *Aldh2$^{-/-}$Fanca$^{-/-}$* embryos that have developmental defects only (purple), developmental delay only (orange), or have both developmental defects and delay (blue). Embryos were generated from either *Aldh2$^{-/-}$Fanca$^{+/-}$* (red mouse, left graph) or *Aldh2$^{+/-}$Fanca$^{+/-}$* (green mouse, right graph) females. The total number of embryos of each genotype is shown above each column.

(B) Pie chart displaying the percentage of developmentally compromised E10.5-E12.5 embryos of all genotypes that have developmental defects only (purple), developmental delay only (orange), or have both developmental defects and delay (blue). Bar graph adjacent to the pie chart shows the percentage of these embryos with eye, cranial or defects of other limbs, or that are pale. Embryos may, and often do, contain a combination of several of these defects. The embryos were generated from either *Aldh2$^{-/-}$Fanca$^{+/-}$* (red mouse, left charts) or *Aldh2$^{+/-}$Fanca$^{+/-}$* (green mouse, right charts) females.

(C) Representative images of developmentally compromised embryos at E10.5-E12.5 in comparison to littermate controls. This is to demonstrate the scoring of various developmental abnormalities. Red arrows denote eye defects. Black arrows denote cranial defects. Blue arrows denote limb defects.

Figure S3, Related to Figure 2B: Survival and eye defects in *Aldh2$^{+/-}$Fanca$^{-/-}$* mice

(A) Kaplan–Meier curve showing the survival of a cohort of *Aldh2$^{+/-}$Fanca$^{-/-}$* mice

born to Aldh2$^{+/+}$Fanca$^{+/-}$, *Aldh2$^{+/-}$Fanca$^{-/-}$* and *Aldh2$^{-/-}$Fanca$^{+/-}$* females, and congenic controls. *Aldh2$^{+/-}$Fanca$^{-/-}$* mice born to Aldh2$^{-/-}$*Fanca$^{+/-}$* mothers have severely reduced survival. The Gehan-Breslow-Wilcoxon test was used to compare survival curves.

(B) Bar graph showing the proportion of mice of each genotype which are normal (blue) or have eye defects (red). The total number of embryos of each genotype is shown above each column. Fisher's exact test was used to compare the proportion of embryos with eye defects between the different genotypes, 5% confidence interval.

Figure S4, Related to Figure 3: Protein alignment of the mouse Aldh2, Aldh1b1 and Aldh1a1 proteins

Three aldehyde dehydrogenases known to catabolize acetaldehyde are aligned using the T-Coffee alignment tool (EMBL-EBI). Dark blue denotes conserved residues. Aldh2 and Aldh1b1 contain a mitochondrial localisation signal.

Figure S5, Related to Figure 5

(A) Rescue of embryonic lethality when *Aldh2$^{-/-}$Fanca$^{-/-}$* mice are carried by *Aldh2$^{+/+}$* mothers

Observed and expected frequencies of P1 pups born from embryo transfer. Fisher's exact test, 5% confidence interval.

(B) A single *Aldh2$^{-/-}$Fanca$^{-/-}$* mouse was born to an *Aldh2$^{+/-}$Fanca$^{+/-}$* female and developed bone marrow failure at 7 weeks of age

(i) The genetic intercross which generated the single *Aldh2$^{-/-}$Fanca$^{-/-}$* mouse.

(ii) Bone marrow histology of a control and the *Aldh2$^{-/-}$Fanca$^{-/-}$* mouse, stained with haematoxylin and eosin (H&E; x20).

(iii) Flow cytometry profiles of bone marrow cells from the *Aldh2$^{-/-}$Fanca$^{-/-}$* mouse and appropriate controls, showing 100,000 lineage-negative events. The *Aldh2$^{-/-}$Fanca$^{-/-}$* mouse has a severely depleted Lin^{-}cKit^{+}Sca1^{+} (LKS) population.

(iv) Flow cytometry profiles of spleen cells from the *Aldh2$^{-/-}$Fanca$^{-/-}$* mouse and appropriate controls, showing 100,000 cells. Cells were stained with B and T cell markers. The *Aldh2$^{-/-}$Fanca$^{-/-}$* mouse has normal numbers of B and T cells in the spleen.

(v) Flow cytometry profiles of spleen cells from the *Aldh2$^{-/-}$Fanca$^{-/-}$* mouse and

appropriate controls, showing 100,000 cells. Cells were stained with markers of erythropoiesis. The *Aldh2*$^{-/-}$*Fanca*$^{-/-}$ mouse shows extramedullary erythropoiesis.

(vi) Flow cytometry profiles of thymic cells from the *Aldh2*$^{-/-}$*Fanca*$^{-/-}$ mouse and appropriate controls, showing 500,000 cells. Cells were stained with markers of T-cells. The *Aldh2*$^{-/-}$*Fanca*$^{-/-}$ mouse has a normal T-cell profile.

Figure S6, Related to Figure 1 and Discussion

(A) *Rev1*$^{-/-}$ B cells and granulocyte-macrophage progenitors are not sensitive to exogenous acetaldehyde

Survival of WT, *Aldh2*$^{-/-}$, *Rev1*$^{-/-}$ and *Fanca*$^{-/-}$ **(i)** granulocyte–macrophage (c.f.u.-GM) progenitors or **(ii)** B cells following exposure to acetaldehyde *in vitro*. Each data point represents the mean of two independent experiments, each carried out in triplicate or quadruplicate respectively. Error bars represent SD.

(B) *Aldh2*$^{-/-}$*Rev1*$^{-/-}$ pups at P1 have an intact haematopoietic stem and progenitor cell (HSPC) population

Representative flow cytometry profiles of HSPCs of P1 pups, showing 10,000 lineage-negative events. The box denotes the Lin^{-}cKit^{+}Sca1^{+} (LKS) population. *Aldh2*$^{-/-}$ and *Aldh2*$^{-/-}$*Rev1*$^{-/-}$ pups were generated from intercrossing *Aldh2*$^{-/-}$*Rev1*$^{+/-}$ females and males. Wild type (WT) and *Rev1*$^{-/-}$ pups were generated from intercrossing *Rev1*$^{+/-}$ females and males.

Supplemental Experimental Procedures

Flow cytometry

Flow cytometry was performed on bone marrow, spleen and thymic cells that were isolated from mutant mice and appropriate controls. Bone marrow cells were obtained either by flushing from the femora and tibiae (adult bones) or by crushing the femora, tibiae and humeri (P1 pups) and passing through a 40-μm filter. The following antibodies were used to stain for the $Lin^{-}cKit^{+}Sca1^{+}$ population of the bone marrow: FITC-conjugated lineage cocktail with antibodies anti-CD4 (clone H129.19, BD Pharmingen), CD3e (clone 145-2C11, eBioscience), Ly-6G/Gr-1 (clone RB6-8C5, eBioscience), CD11b/Mac-1 (clone M1/70, BD Pharmingen), CD45R/B220 (clone RA3-6B2, BD Pharmingen), Fcε R1α (clone MAR-1, eBioscience), CD8a (clone 53-6.7, BD Pharmingen), CD11c (clone N418, eBioscience) and TER-119 (clone Ter119, BD Pharmingen) and anti-c-Kit (PerCP-Cy5.5, clone 2B8, eBioscience) and Sca-1 (PE-Cy7, clone D7, eBioscience) antibodies. B and T cells in the spleen were assessed using anti-CD45R/B220 (APC, clone RA3-6B2, BD Pharmingen), CD3e (PerCPCy5.5, clone 145-2C11, eBioscience), CD4 (FITC, clone H129.19, BD Pharmingen) and CD8a (PE, clone 53-6.7, BD Pharmingen) antibodies. The maturation of the erythroid lineage in the spleen was analysed using antibodies anti-TER-119 (FITC, clone Ter-119, BD Pharmingen) and anti-CD71 (APC, clone C2, BD Pharmingen). Thymic T-cells were assessed using a PE-conjugated lineage cocktail with antibodies anti-Ly-6G/Gr-1 (clone RB6-8C5, eBioscience), CD11b/Mac-1 (clone M1/70, BD Pharmingen), CD45R/B220 (clone RA3-6B2, BD Pharmingen), CD11c (clone N418, eBioscience), CD3e (clone 145-2C11, eBioscience) and TER-119 (clone Ter119, BD Pharmingen), and anti-CD4 (BV421, clone H129.19, BD Pharmingen) and CD8a (APC, clone 53-6.7, BD Pharmingen) antibodies. All antibodies were used at 1:200 and the samples were incubated for 15 min at 4 °C in the dark. Samples were run on a LSRII flow cytometer (BD Pharmingen) and the data were analysed with FlowJo 10.0.7 (Tree Star).

Aldehyde dehydrogenase activity assay

Mouse livers, whole E13.5 embryos or placentas (0.5 g) were finely minced with scissors and homogenised with 500 μl of homogenisation buffer (210 mM mannitol,

70 mM sucrose, 1 mM EDTA, 5 mM MOPS pH 7.4 in H_20) using a tight-fitting Dounce homogeniser. The homogenate was centrifuged at 700 x g for 10 min, the supernatant removed, and centrifuged again at 700 x g for 10 min. The supernatant was removed and centrifuged at 7000 x g for 20 min. The pellet was washed in homogenisation buffer and centrifuged again at 7000 x g for 10 min. The pellet was re-suspended in 300 µl of enzyme assay buffer (10 mM DTT, 20 % glycerol, 0.1 % Triton X-100 and 0.1 M Tris-HCl pH 8.0 in H_20) and centrifuged at 100,000 x g for 30 min at 4 °C to obtain a clear supernatant. Protein concentration was measured using a NanoDrop. To perform the Aldh enzymatic activity assay, a 2 ml reaction was set-up in a cuvette containing 50 mM NaPPi buffer pH 9.0, 2.5 mM NAD^+, 10 mM acetaldehyde and 0.5 mg protein preparation in H_20. The absorbance at 340 nm was recorded using a Cary 5000 UV-Vis-NIR spectrophotometer, at room temperature (RT) for 300 s without the addition of the acetaldehyde substrate, in order to quench the reaction of endogenous aldehydes. After 3 min, the acetaldehyde was added, and the absorbance at 340 nm was recorded for a further 350 s. To calculate the [NADH] production in mols/min/mg total protein, we used: Absorbance = ε x c x L, where ε = 6220 M^{-1}, L = path length (1 cm) and c = [NADH] in mols. The assay was adapted from a protocol by D Mochley-Rosen, Stanford University.

Survival assays of primary mouse B cells

The primary mouse B cells survival assay was performed with lymphocytes purified from the spleen using Lympholyte M (Cederlane). Lymphocytes were stimulated with LPS (Sigma L4391) at a final concentration of 40 µg ml^{-1}. A total of 4×10^5 cells were plated with acetaldehyde at various concentrations in one well of a 24-well plate. After 7 days, the total cells were enumerated, counting 100 images using a ViCell XR (Beckman Coulter). Each data point represents the mean of two independent experiments, each carried out in quadruplicate.

Methylcellulose c.f.u.-GM survival assay

The methylcellulose c.f.u.-GM survival assay was carried out as described previously (Meagher et al., 1982). Briefly, total bone marrow was flushed in IMDM (GIBCO) from the femora and tibiae of mice. Equal numbers of total bone marrow cells were exposed to various concentrations of acetaldehyde *in vitro* for 4 h in a sealed CryoVial. After treatment, two ten-fold serial dilutions of the bone marrow were

made, and these cells were plated in duplicate into 6-well plates with methylcellulose medium with recombinant cytokines (without EPO; MethoCult™ GF M3534). Colonies were counted after 7 days.

Supplemental References

Meagher, R.C., Sieber, F., and Spivak, J.L. (1982). Suppression of Hematopoietic-Progenitor-Cell Proliferation by Ethanol and Acetaldehyde. N. Engl. J. Med. *307*, 845-849.
